# Supplementary material for: cGAS-STING are responsible for premature aging of telomerase-deficient zebrafish
Source: EMBO J. 2025 Jun 9;44(17):4666–80. doi: 10.1038/s44318-025-00482-5 (PMC12402478; doi:10.1038/s44318-025-00482-5)
Supplement: Supplementary file 1 — Appendix [file 44318_2025_482_MOESM1_ESM.pdf]

cGAS-STING are responsible for premature aging of telomerase-deficient zebrafish

Naz Şerifoğlu[1], Giulia Allavena[1], Bruno Lopes-Bastos[1], Marta Marzullo[2,*], Andreia Marques[1,3], Pauline Colibert[1], Pavlos Bousounis[4,5], Eirini Trompouki[1] and Miguel Godinho Ferreira[1]

1- Institute for Research on Cancer and Aging of Nice (IRCAN), CNRS UMR7284, INSERM U1081, Université Cote d'Azur, 06107 Nice, France.

2- Instituto Gulbenkian de Ciência, Oeiras, Portugal

3- Faculty of Medicine, University of Coimbra, Coimbra, Portugal

4- Department of Cellular and Molecular Immunology, Max Planck Institute of Immunobiology and Epigenetics, Freiburg, Germany

5- Faculty of Biology, University of Freiburg, Freiburg, Germany

*Current address: Department of Biology and Biotechnologies, Sapienza University of Rome, Rome, Italy.

Corresponding Author: Miguel-Godinho.FERREIRA@unice.fr

Keywords: Telomerase, cGAS-STING, inflammation, aging, zebrafish

Table of contents

Page 2: Appendix Figure S1: Southern blots for TRF assay

Page 3: Appendix Figure S2: G2 *tert-/- sting-/-* fish recapitulate feature of G1 fish

Page 5: Appendix Figure S3: Expression levels of TEs and RNA sensor pathway

Page 7: Appendix Figure S4: Histology and expression of YAP-TAZ targets.

Page 8: Appendix Figure S5: Male fertility.

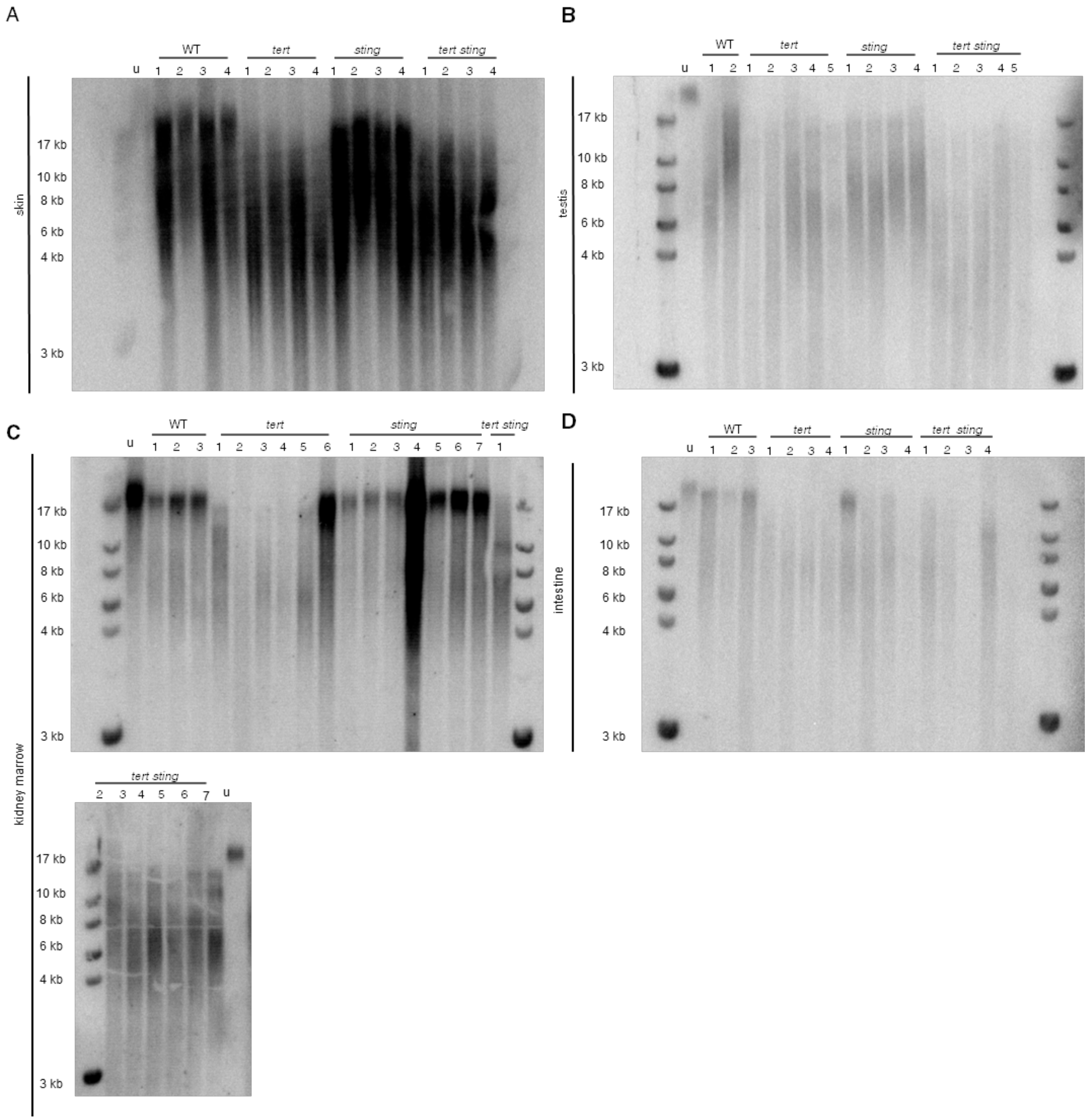

Appendix Figure S1: Southern blots for TRF assay. **a**, representative image for mean telomere length measured by TRF analysis in the skin (n_{WT}= 5, $n_{tert-/-}$=5, $n_{sting-/-}$=5, $n_{tert-/-\ sting-/-}$=4). **b**, representative image for mean telomere length measured by TRF analysis in the testis (n_{WT}= 4, $n_{tert-/-}$=6, $n_{sting-/-}$=5, $n_{tert-/-\ sting-/-}$=6). **c**, representative image for mean telomere length measured by TRF analysis in the kidney marrow (n_{WT}= 3, $n_{tert-/-}$=4, $n_{sting-/-}$=7, $n_{tert-/-\ sting-/-}$=7) **d**, representative image for mean telomere length measured by TRF analysis in the intestine (n_{WT}= 6, $n_{tert-/-}$=6, $n_{sting-/-}$=6, $n_{tert-/-\ sting-/-}$=6).

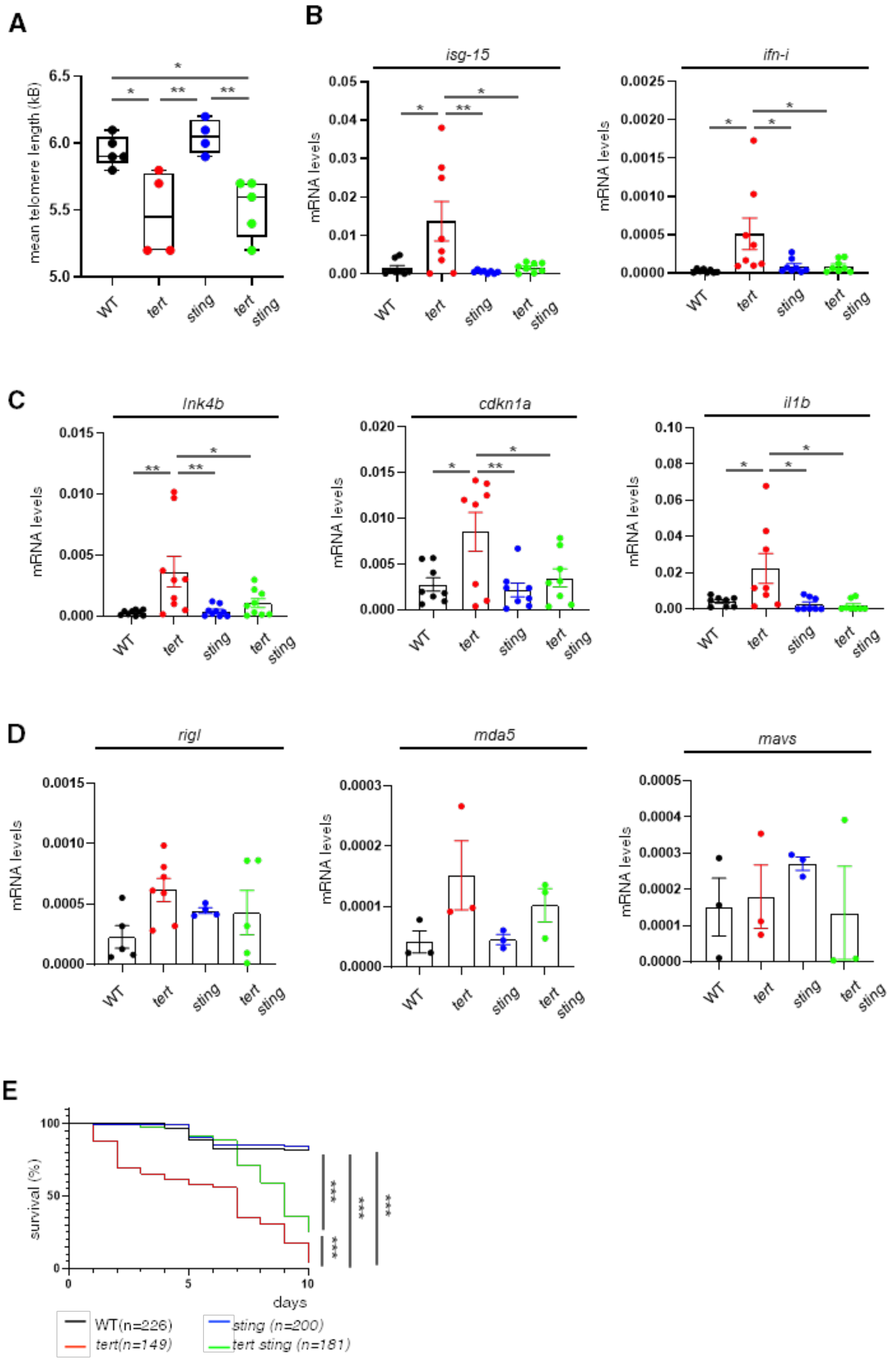

Appendix Figure S2: G2 tert-/- sting-/- fish recapitulate feature of G1 fish

a, quantification of mean telomere length measured by TRF analysis in G2 tert-/- larvae (n_{WT}= 5, $n_{tert-/-}$=4, $n_{sting-/-}$=4, $n_{tert-/-\ sting-/-}$=5, WT vs tert-/- p= 0.022, tert-/- vs *sting-/-* p= 0.007, sting-/- *vs tert-/- sting-/-* p=0.009, WT vs tert-/- sting-/- p= 0.028). The boxes represent the interquartile range (IQR; 25th to 75th percentile). Inside each box, the horizontal line indicates the median. The whiskers extend to the most extreme data points (minima and maxima) within 1.5×IQR from the quartiles. **b,** RT-PCR analysis of isg-15 gene expression (n_{WT}= 8, $n_{tert-/-}$=8, $n_{sting-/-}$=8, $n_{tert-/-sting-/-}$=8, WT vs tert-/- p= 0.012, tert-/- vs *sting-/-* p= 0.006, tert-/- *vs tert-/- sting-/-* p=0.013) and *ifn-i* gene expression (n_{WT}= 8, $n_{tert-/-}$=8, $n_{sting-/-}$=8, $n_{tert-/-sting-/-}$=8, WT vs tert-/- p= 0.015, tert-/- vs sting-/- p= 0.041, tert-/- vs tert-/- sting-/- p=0.041). **c,** RT-PCR analysis of ink4b gene expression (n_{WT}= 9, $n_{tert-/-}$=9, $n_{sting-/-}$=9, $n_{tert-/-sting-/-}$=9, WT vs tert-/- p= 0.005, tert-/- vs *sting-/-* p= 0.008, tert-/- *vs tert-/- sting-/-* p=0.044), cdkn1a gene expression (n_{WT}= 8, $n_{tert-/-}$=8, $n_{sting-/-}$=8, $n_{tert-/-sting-/-}$=8, WT vs tert-/- p= 0.018, tert-/- vs sting-/- p= 0.008, tert-/- vs tert-/- sting-/- p=0.045) and il1b gene expression (n_{WT}= 8, $n_{tert-/-}$=8, $n_{sting-/-}$=8, $n_{tert-/-sting-/-}$=8, WT vs tert-/- p= 0.026, tert-/- vs *sting-/-* p= 0.014, tert-/- *vs tert-/- sting-/-* p=0.011). **d,** RT-PCR analysis of *rigI* gene expression (n_{WT}= 5, $n_{tert-/-}$=7, $n_{sting-/-}$=4, $n_{tert-/-sting-/-}$=5), mda5 gene expression (n_{WT}= 3, $n_{tert-/-}$=3, $n_{sting-/-}$=3, $n_{tert-/-sting-/-}$=3) and mavs gene expression (n_{WT}= 3 , $n_{tert-/-}$=3, $n_{sting-/-}$=3, $n_{tert-/-sting-/-}$=3). **e,**

quantification of survival (n_{WT}= 226, $n_{tert\text{-}/\text{-}}$=149, $n_{sting\text{-}/\text{-}}$=200, $n_{tert\text{-}/\text{-}\ sting\text{-}/\text{-}}$=181, WT vs *tert-/-* p=0,000001*, sting-/- vs tert-/- p=0.000001, tert-/-* vs *tert-/- sting-/-* p=0,000001, WT vs *tert-/- sting-/-* p=0,000001). Data are presented as the mean ± s.e.m. *p<0.05; **p<0.01, ***p<0.001, using a one-way ANOVA and post hoc Tukey test. Survival data were analyzed using Log-rank tests, ***p<0.001.

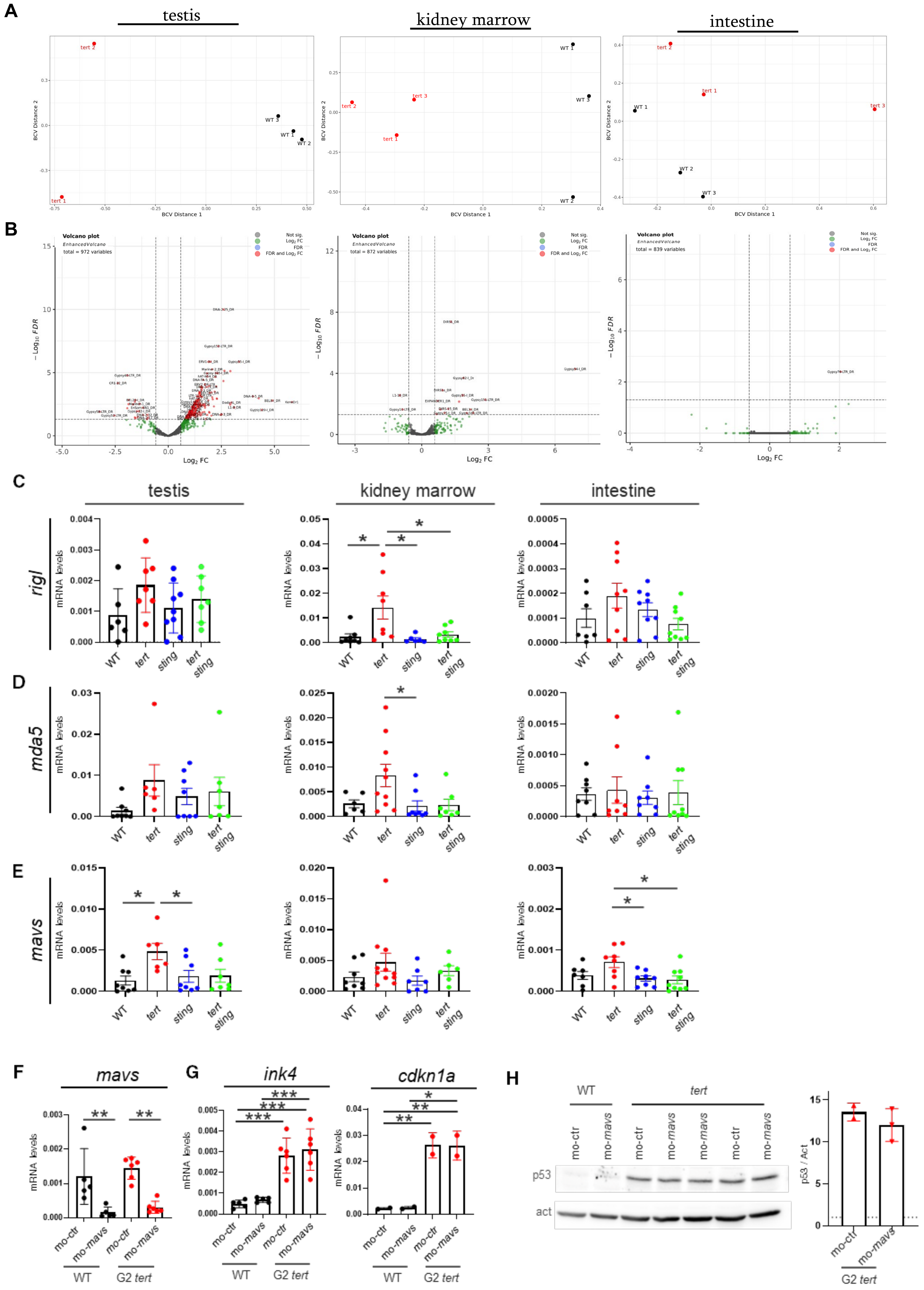

52 **Appendix Figure S3: Expression levels of TEs and RNA sensor pathway. a,** Multidimensional scaling (MDS)

plots of TE expression in WT and *tert* mutant fish. **b**, Vulcano plot of TE expressed in WT and *tert* mutant. **c**, RT-PCR analysis of *rigI* gene expression in testis (n_{WT}= 6, $n_{tert-/-}$=9, $n_{sting-/-}$=9, $n_{tert-/-sting-/-}$=8), in kidney marrow (n_{WT}= 8, $n_{tert-/-}$=8, p=0.038, $n_{sting-/-}$=6, $n_{tert-/-sting-/-}$=8, WT *vs tert-/-* p= 0.015, *tert-/- vs sting-/-* p= 0.015, *tert-/- vs tert-/- sting-/-* p=0.028) and in intestine (n_{WT}= 7, $n_{tert-/-}$=9, $n_{sting-/-}$=9, $n_{tert-/-sting-/-}$=9). **d**, RT-PCR analysis of *mda5* gene expression in testis (n_{WT}= 8, $n_{tert-/-}$=6, $n_{sting-/-}$=8, $n_{tert-/-sting-/-}$=7), in kidney marrow (n_{WT}= 6, $n_{tert-/-}$=10, $n_{sting-/-}$=8, $n_{tert-/-sting-/-}$=7, *tert-/- vs sting-/-* p= 0.047) and in intestine (n_{WT}= 8, $n_{tert-/-}$=8, $n_{sting-/-}$=8, $n_{tert-/-sting-/-}$=9). **e**, RT-PCR analysis of *mavs* gene expression in testis (n_{WT}= 8, $n_{tert-/-}$=6, $n_{sting-/-}$=8, $n_{tert-/-sting-/-}$=7, WT *vs tert-/-* p= 0.016, *tert-/- vs sting-/-* p= 0.046), in kidney marrow (n_{WT}= 8, $n_{tert-/-}$=11, $n_{sting-/-}$=7, $n_{tert-/-sting-/-}$=6) and in intestine (n_{WT}= 7, $n_{tert-/-}$=8, $n_{sting-/-}$=8, $n_{tert-/-sting-/-}$=9, *tert-/- vs sting-/-* p= 0.040, *tert-/- vs tert-/- sting-/-* p=0.019). **f,** RT-PCR analysis of *mavs* gene expression in G2 fish injected with morpholino control (mo-ctr) or against *mavs* (mo-*mavs*) ($n_{WT\ mo-ctr}$= 5, $n_{WT\ mo-mavs}$= 5 $n_{tert-/-\ mo-ctr}$=6, $n_{tert-/-\ mo-mavs}$=6, WT mo-ctr *vs* WT mo-*mavs* p= 0.007, G2 *tert-/-* mo-ctr *vs* G2 *tert-/-* mo-*mavs* p= 0.001). **g,** RT-PCR analysis of *ink4* gene expression in morpholino injected fish ($n_{WTmo-\ ctr}$= 5, $n_{WTmo-\ mavs}$= 5 $n_{tert-/-mo-\ ctr}$=6, $n_{tert-/-mo-\ mavs}$=6, WT mo-ctr *vs* G2 *tert-/-* mo-ctr p=0,0002, WT mo-ctr *vs* G2 *tert-/-* mo-*mavs* p=0,00004, WT mo-*mavs vs* G2 *tert-/-* mo-*mavs* p=0,0001) and *cdkn1a* ($n_{WT\ mo-ctr}$=2, $n_{WT\ mo-mavs}$=2 $n_{tert-/-\ mo-ctr}$=2, $n_{tert-/-\ mo-mavs}$=2, WT mo-ctr *vs* G2 *tert-/-* mo-ctr p=0,01, WT mo-ctr *vs* G2 *tert-/-* mo-*mavs* p=0,01, WT mo-*mavs vs* G2 *tert-/-* mo-*mavs* p=0,01). **h,** western blot images and quantification of p53 in G2 *tert-/-* fish injected with mo-ctr or mo-*mavs* ($n_{tert-/-\ mo-ctr}$=2, $n_{tert-/-\ mo-mavs}$=3). RNA expression data are presented as the mean ± s.e.m. *p<0.05; **p<0.01, ***p<0.001, using a one-way ANOVA and post hoc Tukey test.

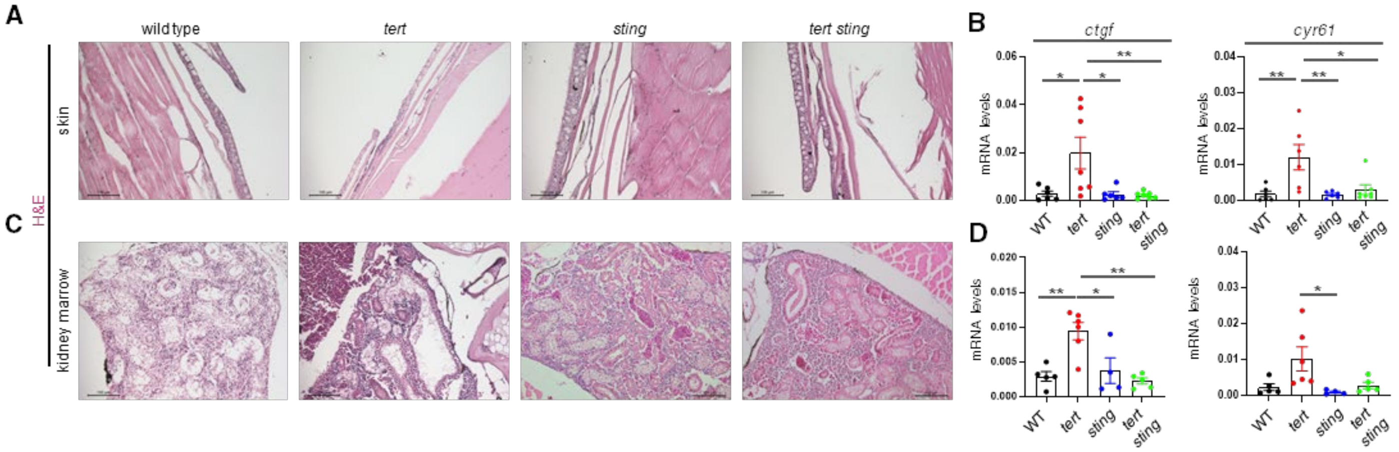

Appendix Figure S4: Histology and expression of YAP-TAZ targets. a Representative hematoxylin eosin staining of skin (n_{WT}= 4, $n_{tert-/-}$=4, $n_{sting-/-}$=4, $n_{tert-/-\ sting-/-}$=4). **b**, RT-qPCR analysis of YAP-TAZ pathway targets in the skin (n_{WT}= 6, $n_{tert-/-}$=6-7, $n_{sting-/-}$=6, $n_{tert-/-\ sting-/-}$=7, *ctfg:* WT vs *tert-/- p=0.018, sting-/-* vs *tert-/- p=0.016, tert-/-* vs *tert-/- sting-/- p=0.010; cyr61:* WT vs *tert-/- p=0.007, sting-/-* vs *tert-/- p=0.006, tert-/-* vs *tert-/- sting-/- p=0.001*). **c**, representative hematoxylin eosin staining of kidney marrow (n_{WT}= 4, $n_{tert-/-}$=4, $n_{sting-/-}$=4, $n_{tert-/-\ sting-/-}$=4). **d**, RT-qPCR analysis of YAP-TAZ pathway targets in the kidney marrow (n_{WT}= 5, $n_{tert-/-}$=6-7, $n_{sting-/-}$=4, $n_{tert-/-\ sting-/-}$=5, *ctgf:* WT vs *tert-/- p=0.003, sting-/-* vs *tert-/- p=0.014, tert-/-* vs *tert-/- sting-/- p=0,001; cyr61: sting-/-* vs *tert-/- p=0.039*). Data are presented as the mean ± s.e.m. *p<0.05; **p<0.01, ***p<0.001, using a one-way ANOVA and post hoc Tukey test.

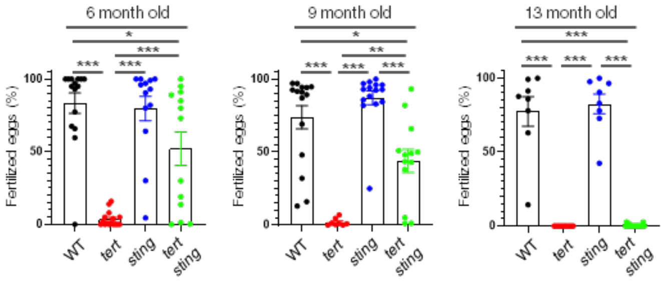

Appendix Figure S5: Male fertility. quantification of male fertile capacity at 6 months of age(n_{WT}= 14, $n_{tert\text{-}/\text{-}}$ =16, $n_{sting\text{-}/\text{-}}$=13, $n_{tert\text{-}/\text{-}\ sting\text{-}/\text{-}}$=13), WT vs *tert-/- p=0.000001, sting-/- vs tert-/- p=0.000001,* WT vs *tert-/- sting-/- p=0.025, tert-/-* vs *tert-/- sting-/- p=0,0001)*, at 9 months of age (n_{WT}= 15, $n_{tert\text{-}/\text{-}}$=10, $n_{sting\text{-}/\text{-}}$=14, $n_{tert\text{-}/\text{-}sting\text{-}/\text{-}}$ =13), WT vs *tert-/- p=0.000001, sting-/- vs tert-/- p=0.000001,,* WT vs *tert-/- sting-/- p=0.011, sting-/- vs tert-/- sting-/- p=0.0001, tert-/-* vs *tert-/- sting-/- p=0.002)*, and at 13 months of age (n_{WT}= 8, $n_{tert\text{-}/\text{-}}$=11, $n_{sting\text{-}/\text{-}}$=8, $n_{tert\text{-}/\text{-}\ sting\text{-}/\text{-}}$=17), WT vs *tert-/- p=0.000001, sting-/- vs tert-/- p=0.000001,* WT vs *tert-/- sting-/- p=0,000001, sting-/- vs tert-/- sting-/- p=0.000001)*. Data are presented as the mean ± s.e.m. *p<0.05; **p<0.01, ***p<0.001, using a one-way ANOVA and post hoc Tukey test.
