# Supplementary material for: cGAS-STING are responsible for premature aging of telomerase-deficient zebrafish
Source: EMBO J. 2025 Jun 9;44(17):4666–80. doi: 10.1038/s44318-025-00482-5 (PMC12402478; doi:10.1038/s44318-025-00482-5)
Supplement: Supplementary file 3 — Source data Fig. 1 [file 44318_2025_482_MOESM3_ESM.zip › Fig1 new 1 and 2/Fig 1F new 2A/tbk1 irf3 wb.pptx]

## Slide 1
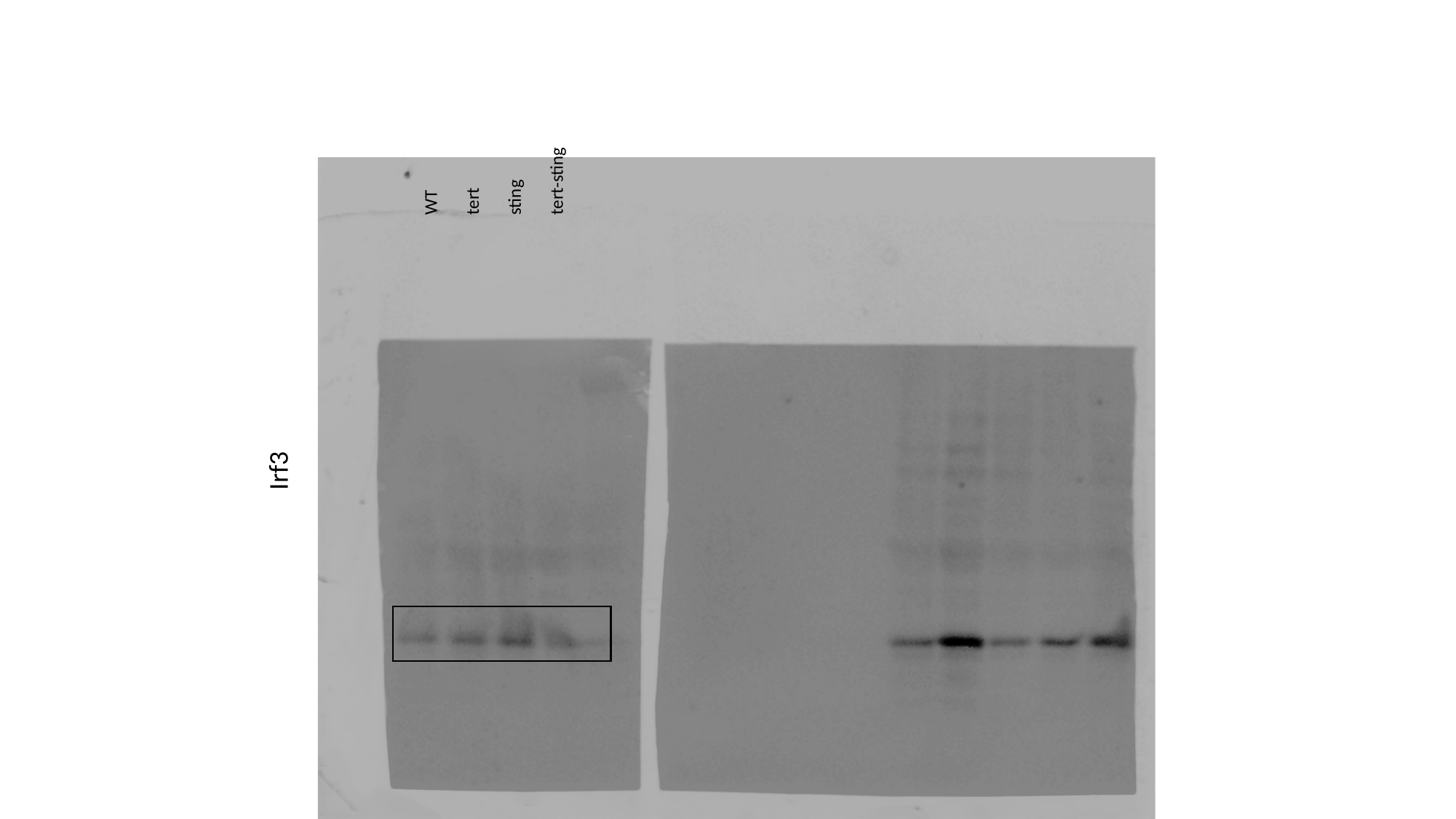

| WT | tert | sting | tert-sting |
| --- | --- | --- | --- |
Irf3

## Slide 2
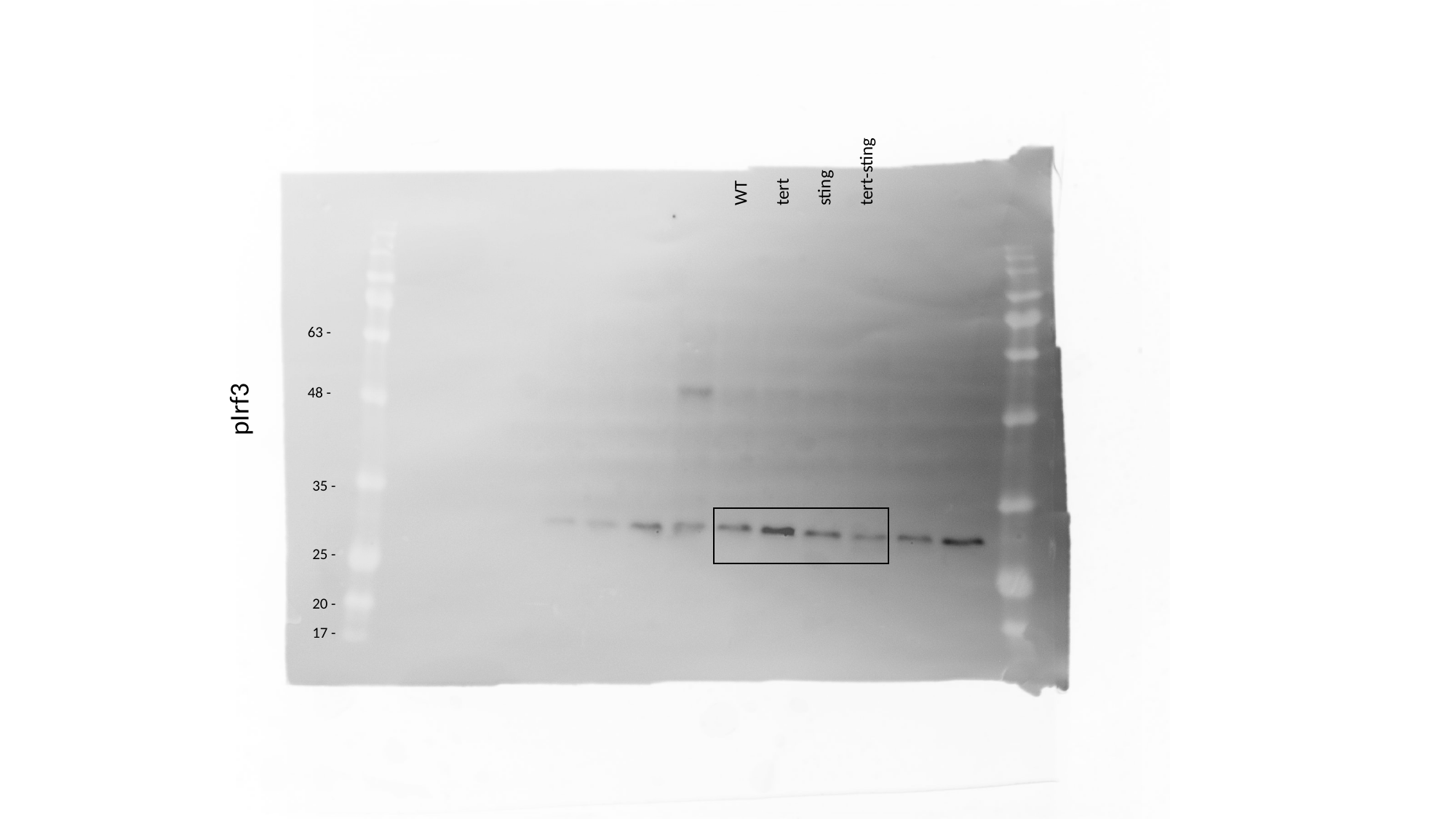

| WT | tert | sting | tert-sting |
| --- | --- | --- | --- |
63 -
48 -
pIrf3
35 -
25 -
20 -
17 -

## Slide 3
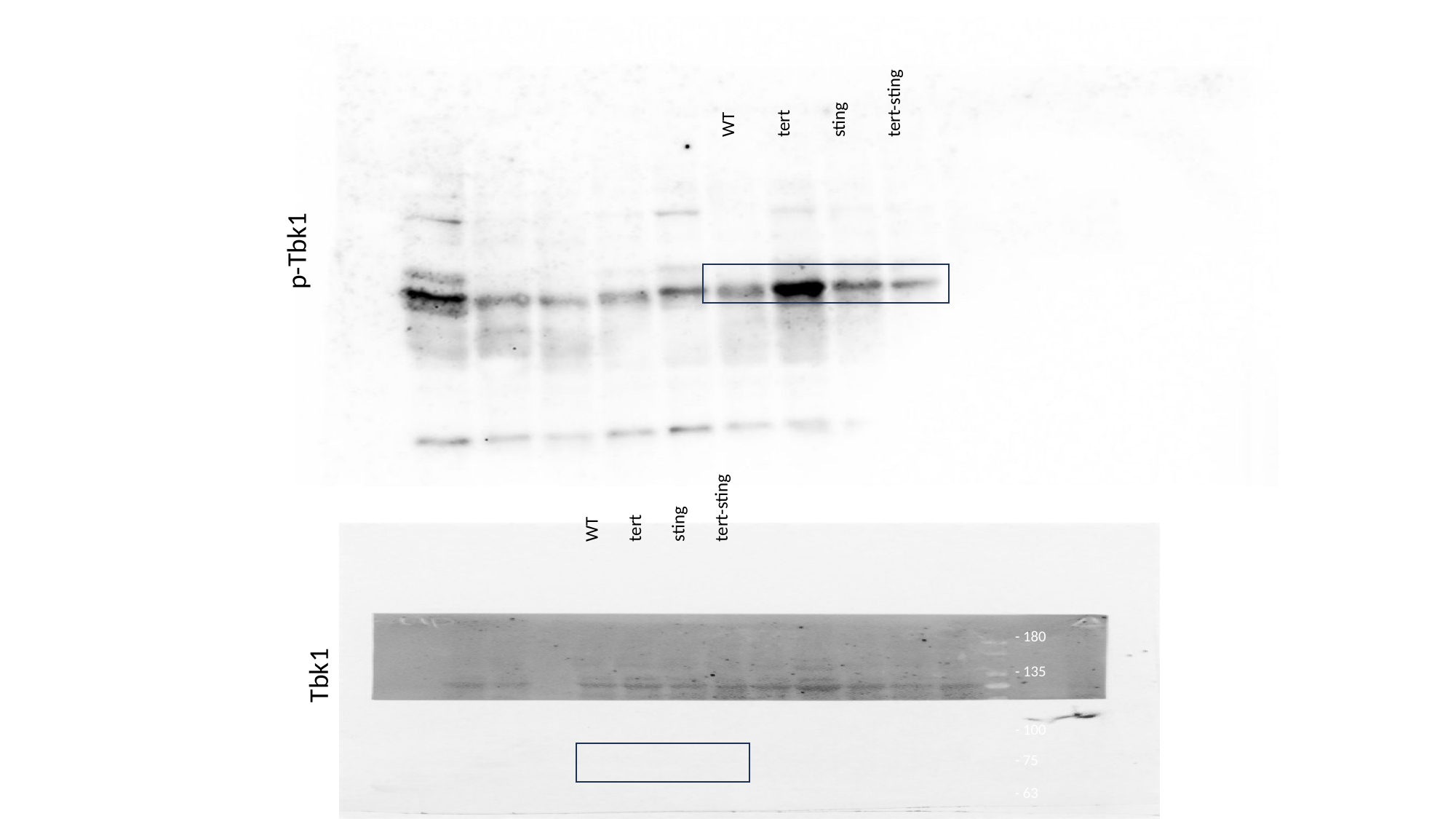

| WT | tert | sting | tert-sting |
| --- | --- | --- | --- |
p-Tbk1
| WT | tert | sting | tert-sting |
| --- | --- | --- | --- |
- 180
Tbk1
- 135
- 100
- 75
- 63
