# Supplementary figures and images for: cGAS-STING are responsible for premature aging of telomerase-deficient zebrafish
Source: EMBO J. 2025 Jun 9;44(17):4666–80. doi: 10.1038/s44318-025-00482-5 (PMC12402478; doi:10.1038/s44318-025-00482-5)

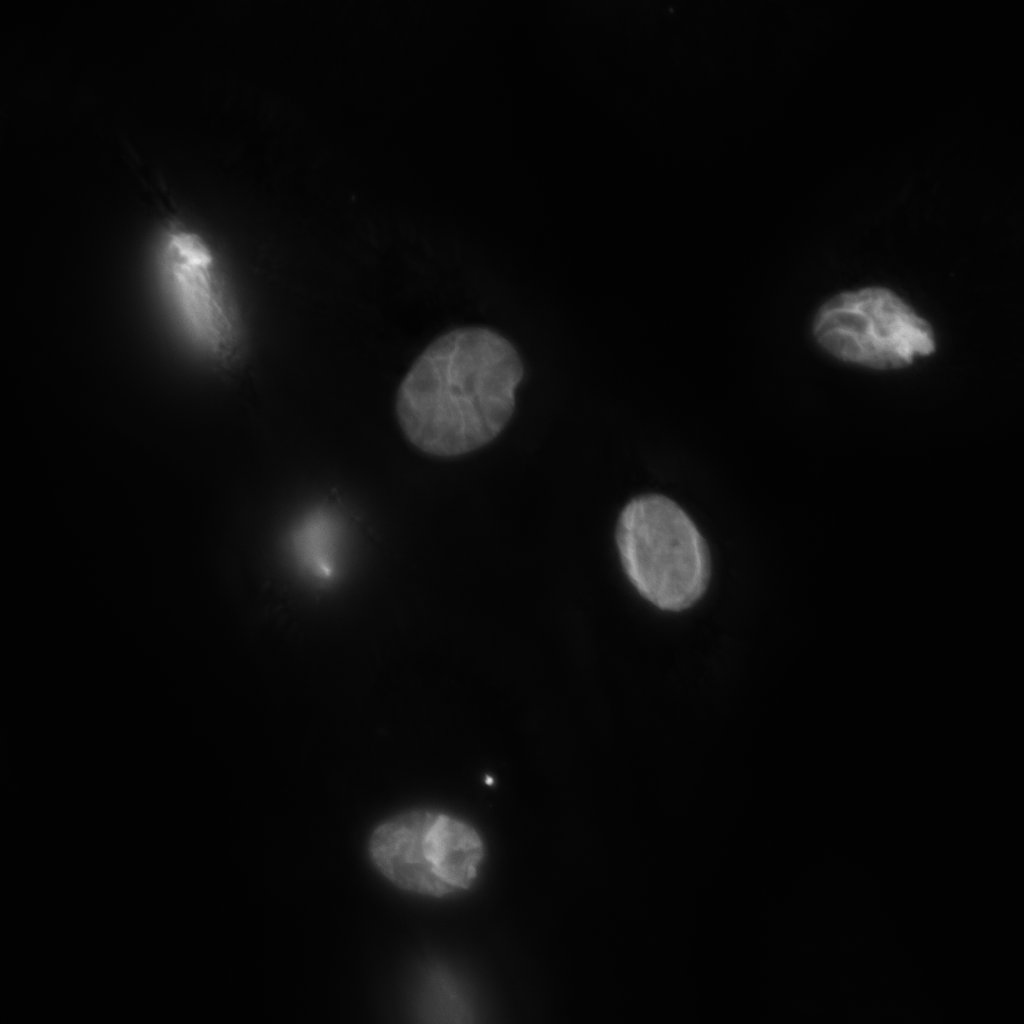

Supplement: Supplementary file 3 — Source data Fig. 1 [file 44318_2025_482_MOESM3_ESM.zip › Fig1 new 1 and 2/Fig 1E/1E/STING.jpg]

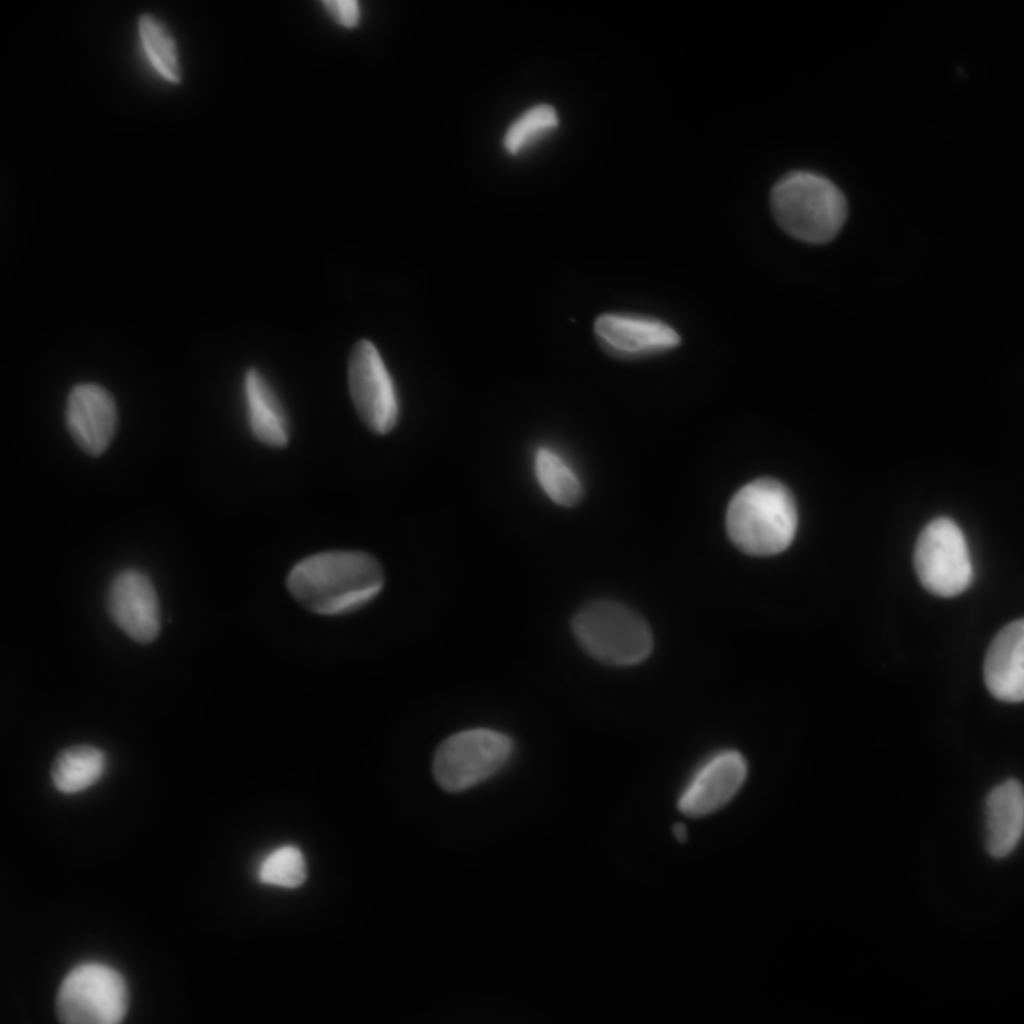

Supplement: Supplementary file 3 — Source data Fig. 1 [file 44318_2025_482_MOESM3_ESM.zip › Fig1 new 1 and 2/Fig 1E/1E/Tert.jpg]

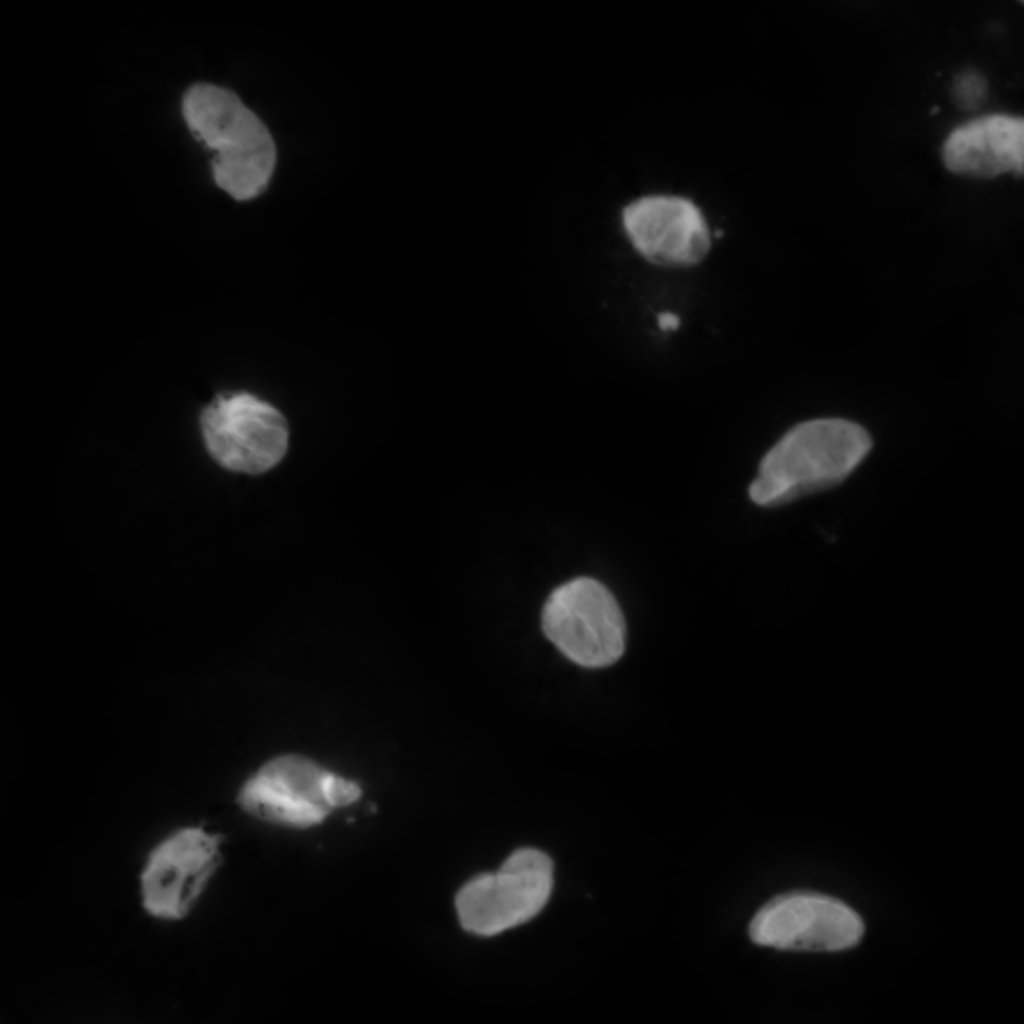

Supplement: Supplementary file 3 — Source data Fig. 1 [file 44318_2025_482_MOESM3_ESM.zip › Fig1 new 1 and 2/Fig 1E/1E/TERT_STING.jpg]

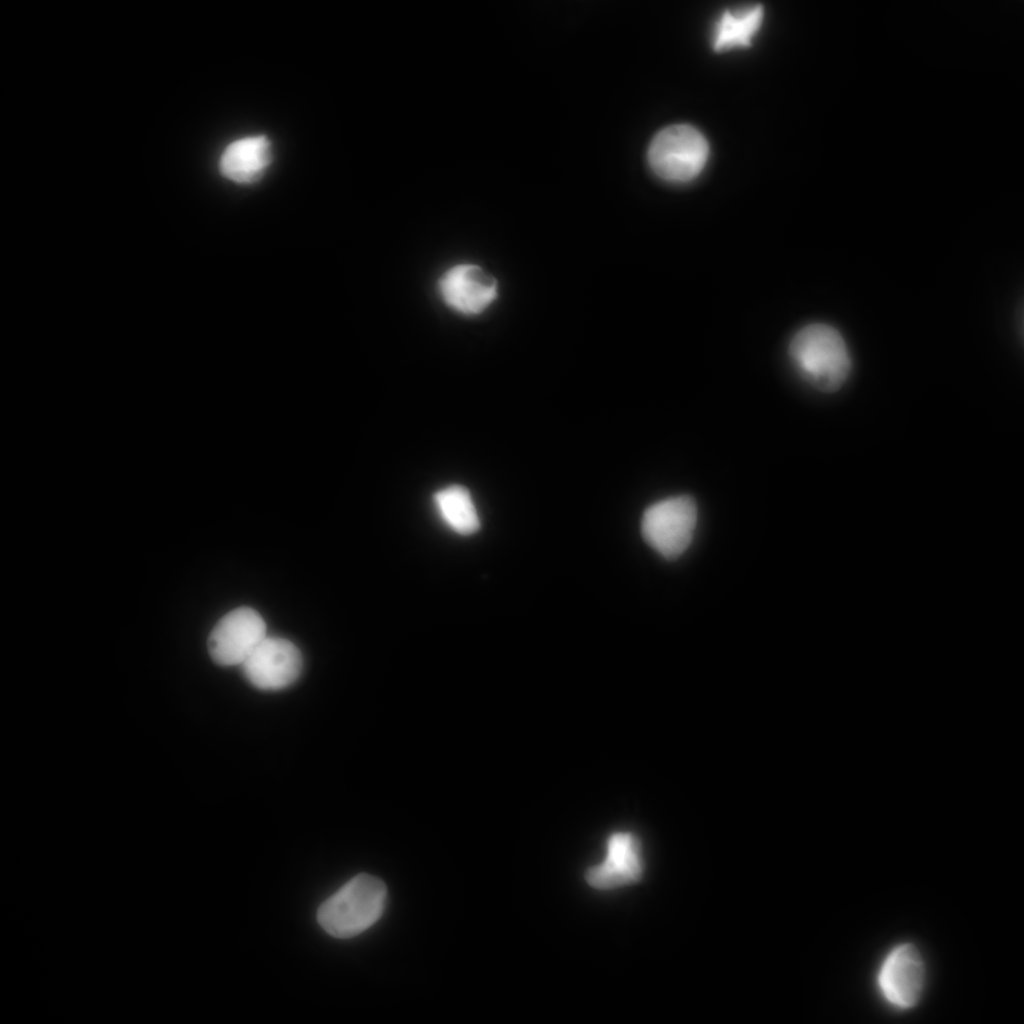

Supplement: Supplementary file 3 — Source data Fig. 1 [file 44318_2025_482_MOESM3_ESM.zip › Fig1 new 1 and 2/Fig 1E/1E/WT.jpg]

## Slide 1
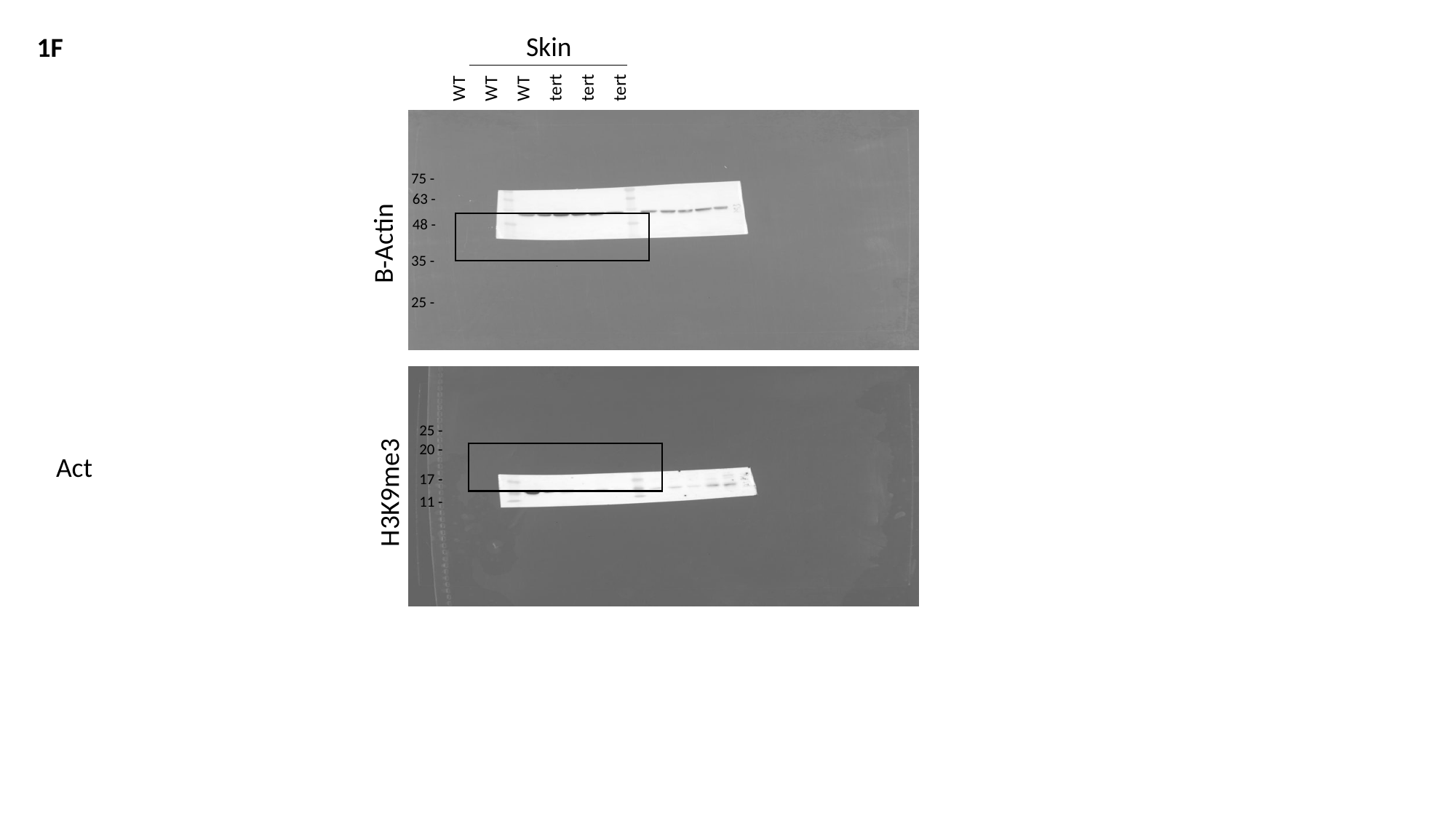

Skin
1F
| WT | WT | WT | tert | tert | tert |
| --- | --- | --- | --- | --- | --- |
75 -
63 -
48 -
B-Actin
35 -
25 -
25 -
20 -
Act
17 -
H3K9me3
11 -

Supplement: Supplementary file 3 — Source data Fig. 1 [file 44318_2025_482_MOESM3_ESM.zip › Fig1 new 1 and 2/Fig 1F/1F blot.pptx]

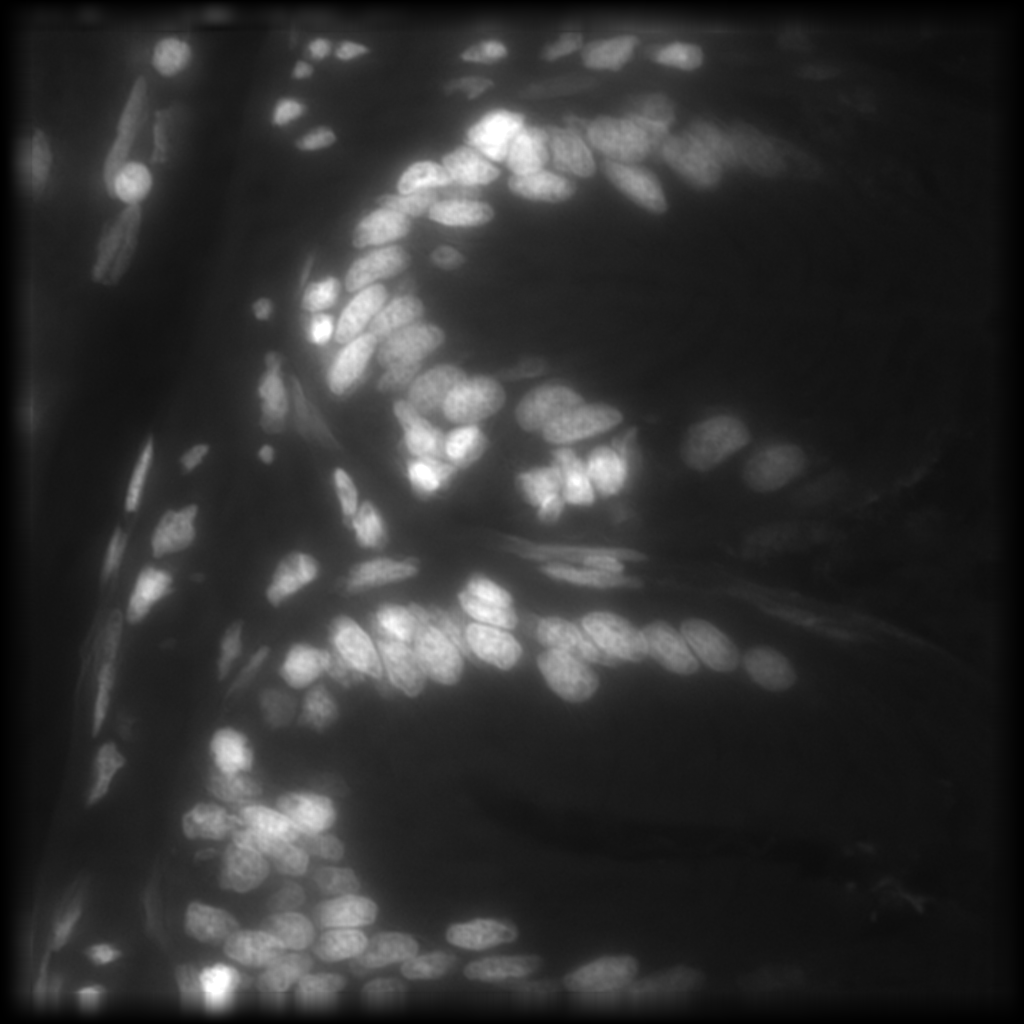

Supplement: Supplementary file 4 — Source data Fig. 2 [file 44318_2025_482_MOESM4_ESM.zip › Fig2 new 3/Fig 2A new 3A/H2AX intestine/sting-_- gH2AX 60x intestine.tif]

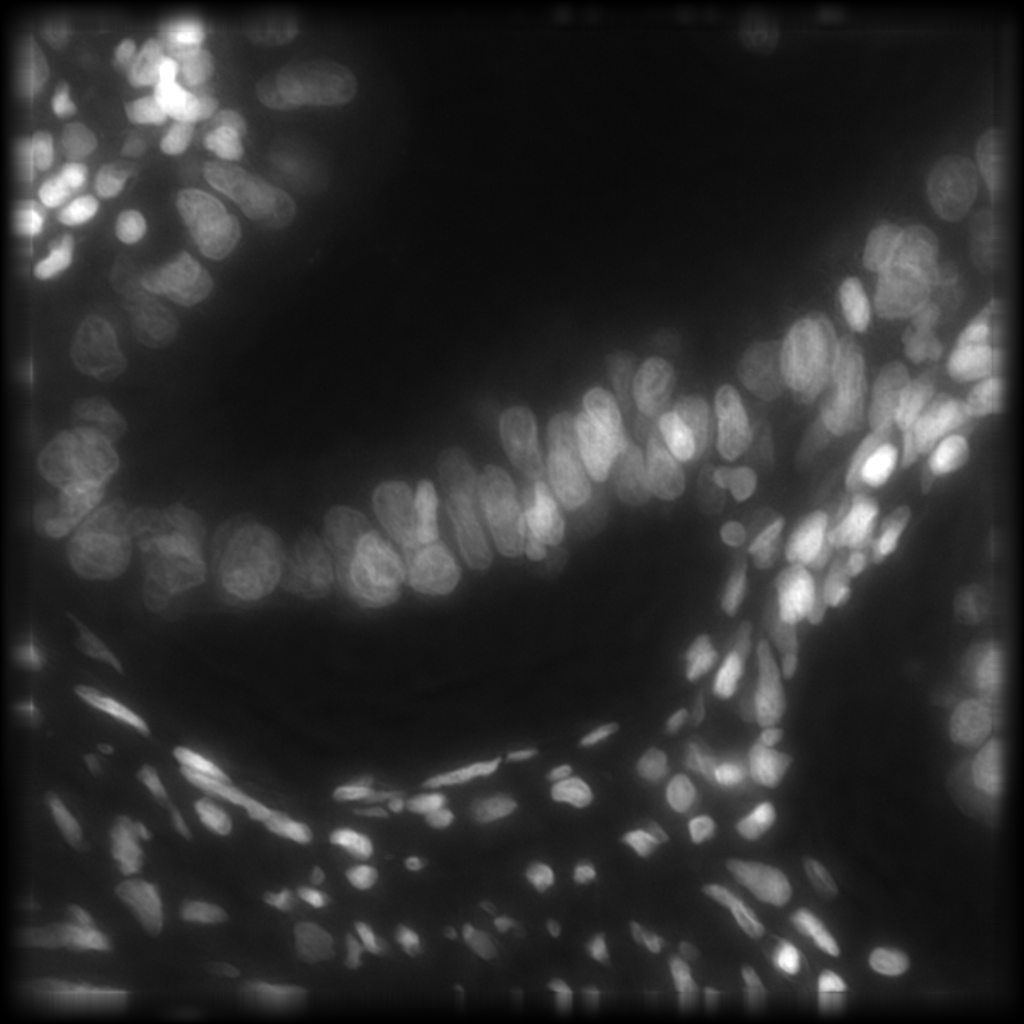

Supplement: Supplementary file 4 — Source data Fig. 2 [file 44318_2025_482_MOESM4_ESM.zip › Fig2 new 3/Fig 2A new 3A/H2AX intestine/tert - sting -_- 60x gH2AXintestine.tif]

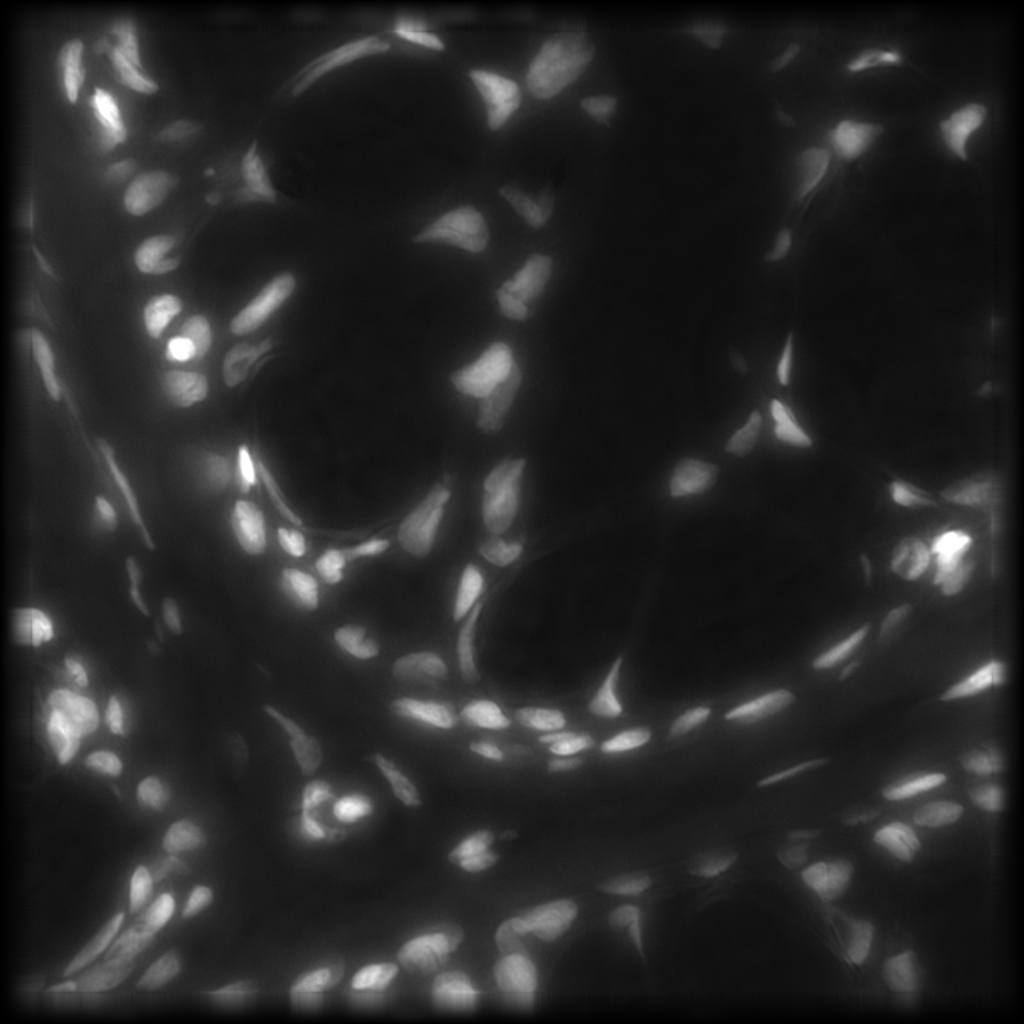

Supplement: Supplementary file 4 — Source data Fig. 2 [file 44318_2025_482_MOESM4_ESM.zip › Fig2 new 3/Fig 2A new 3A/H2AX intestine/tert -_- gH2AX 60x intestine.tif]

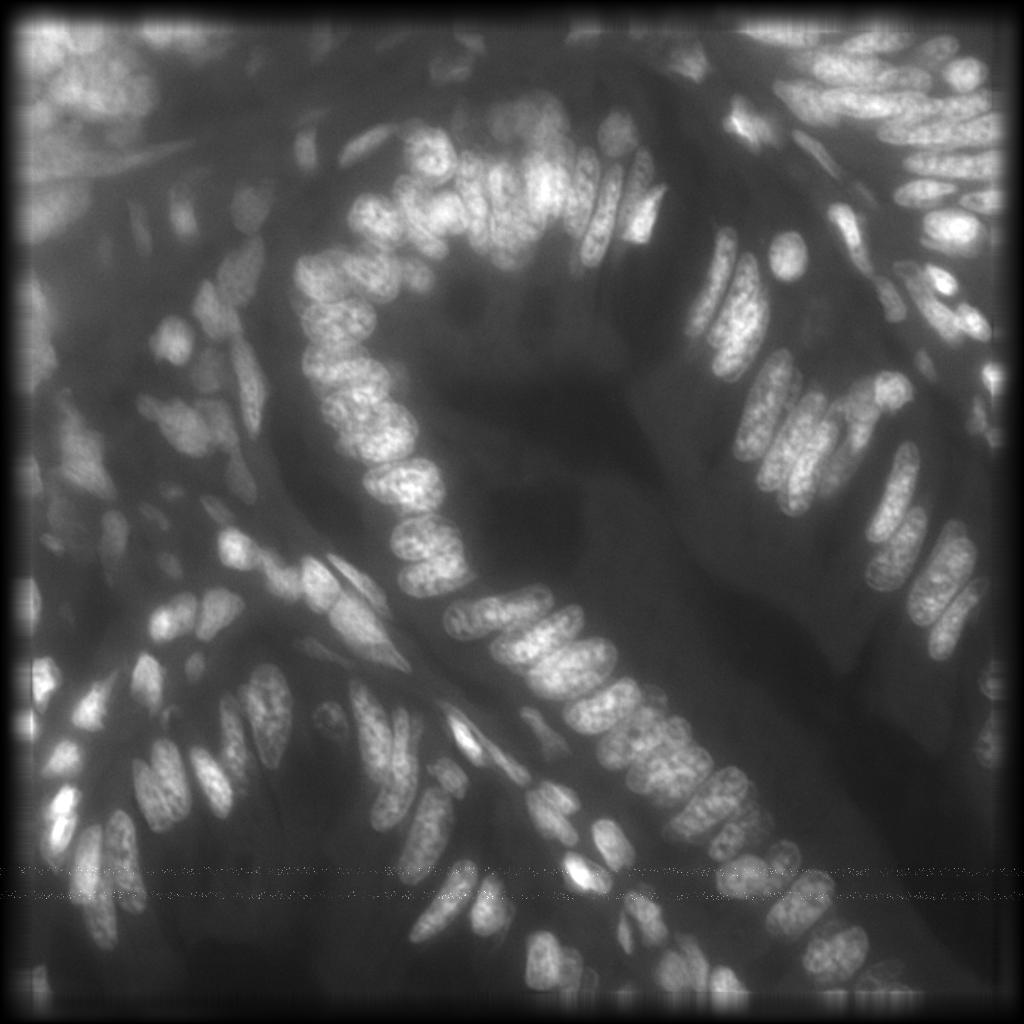

Supplement: Supplementary file 4 — Source data Fig. 2 [file 44318_2025_482_MOESM4_ESM.zip › Fig2 new 3/Fig 2A new 3A/H2AX intestine/wt 60x gH2AX intestine.tif]

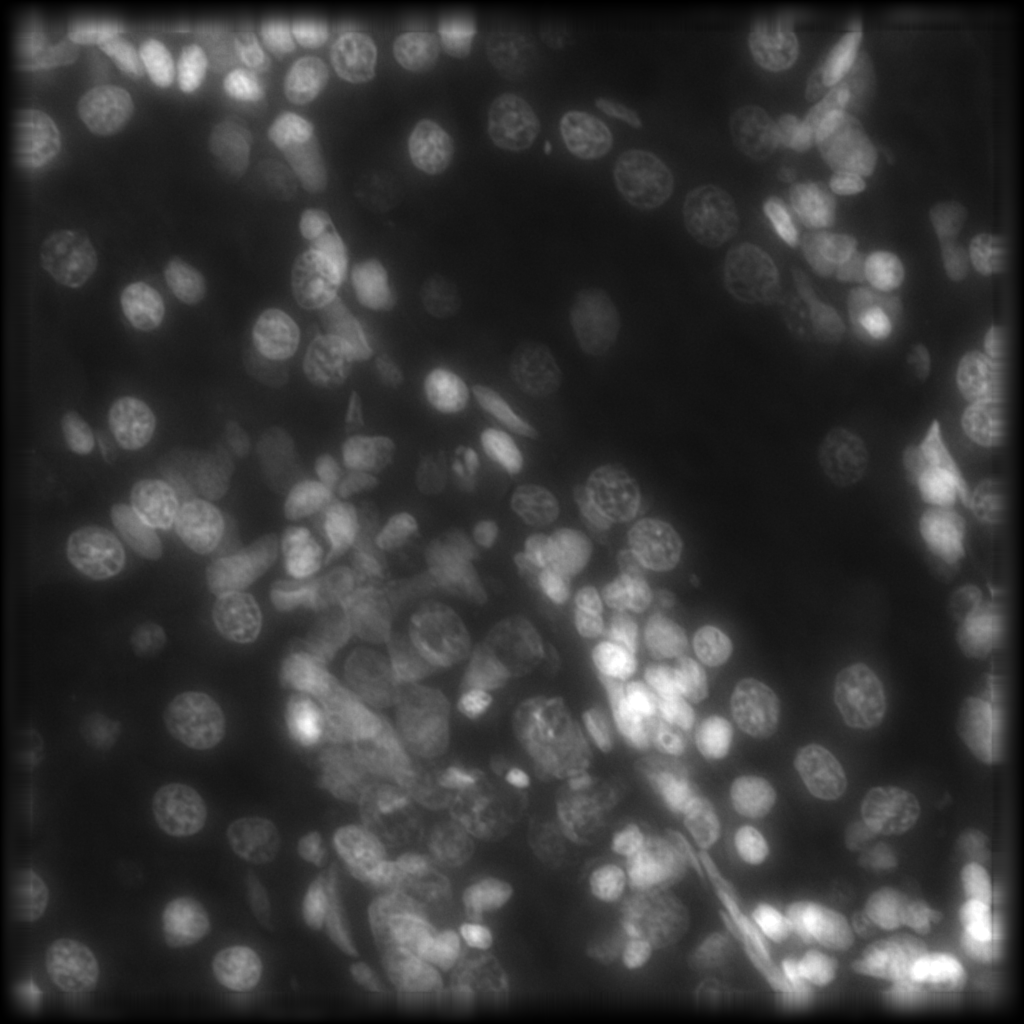

Supplement: Supplementary file 4 — Source data Fig. 2 [file 44318_2025_482_MOESM4_ESM.zip › Fig2 new 3/Fig 2A new 3A/H2AX km/sting -_- 60x gH2AX kidney marrow.tif]

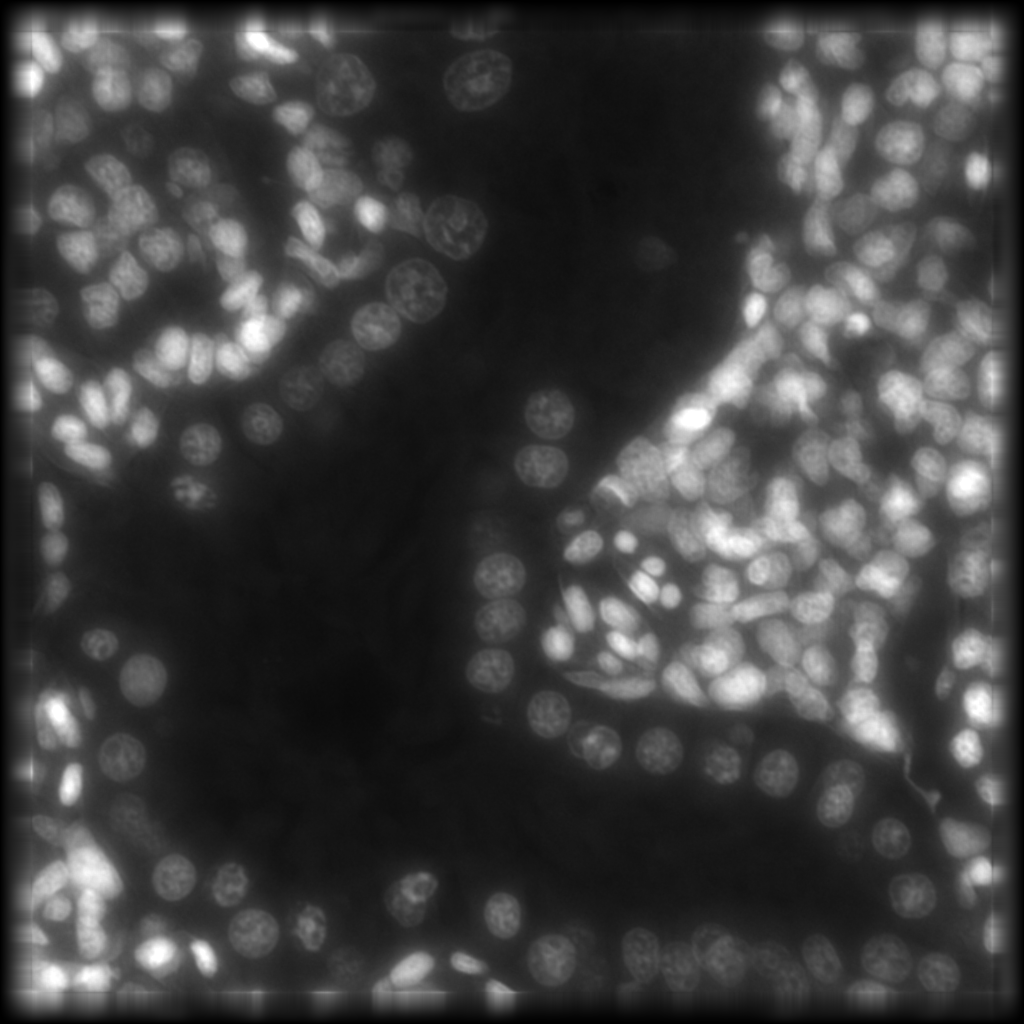

Supplement: Supplementary file 4 — Source data Fig. 2 [file 44318_2025_482_MOESM4_ESM.zip › Fig2 new 3/Fig 2A new 3A/H2AX km/tert -_- sting -_- 60x gH2AX kidney marrow.tif]

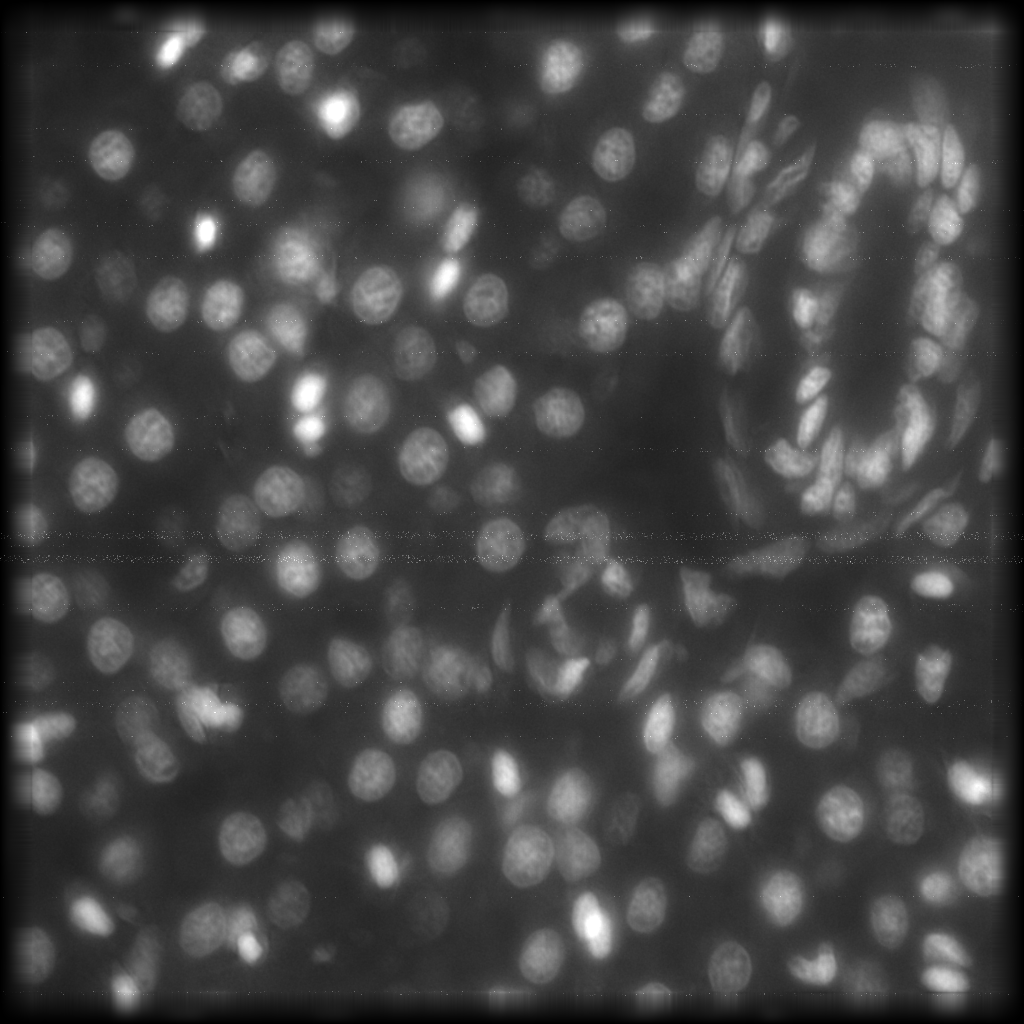

Supplement: Supplementary file 4 — Source data Fig. 2 [file 44318_2025_482_MOESM4_ESM.zip › Fig2 new 3/Fig 2A new 3A/H2AX km/wt 60x gH2AX kidney marrow.tif]

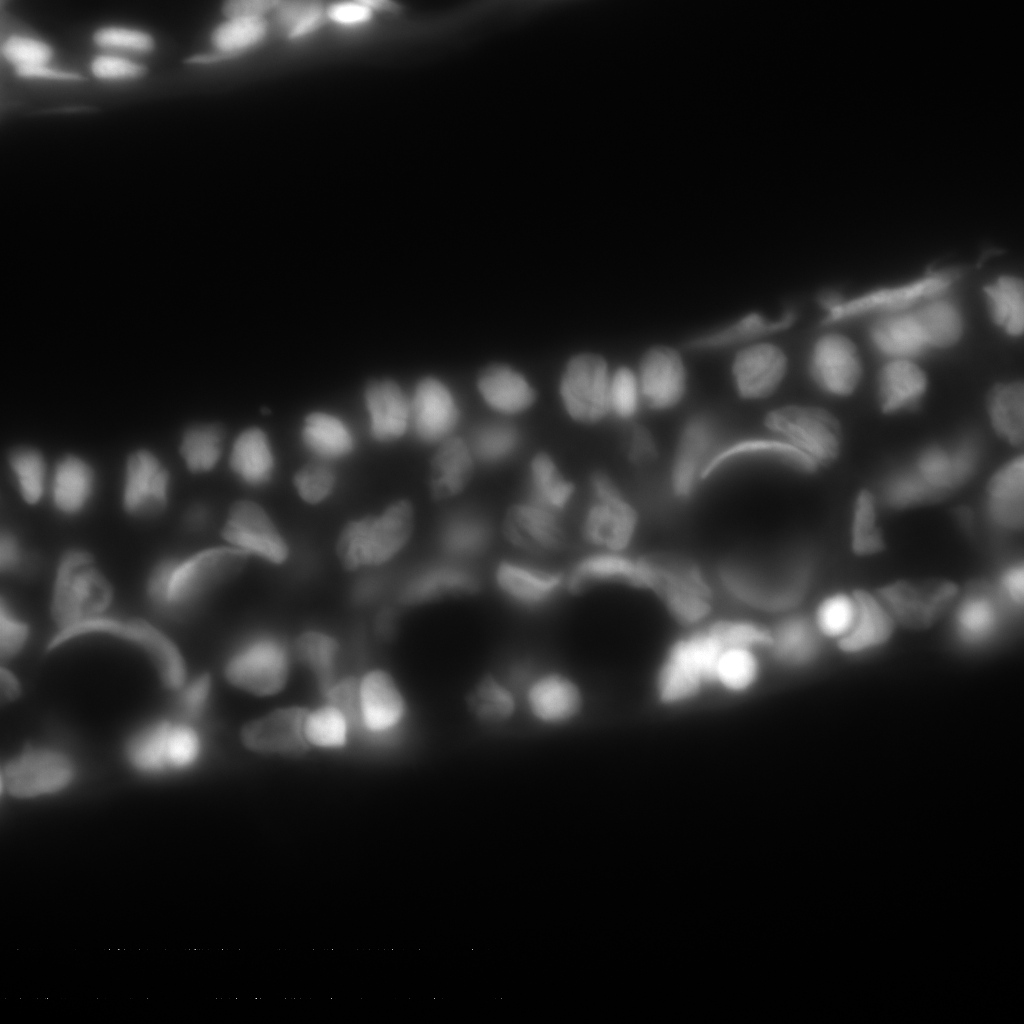

Supplement: Supplementary file 4 — Source data Fig. 2 [file 44318_2025_482_MOESM4_ESM.zip › Fig2 new 3/Fig 2A new 3A/H2AX skin/sting -_- 60x gH2AX skin (updated).tif]

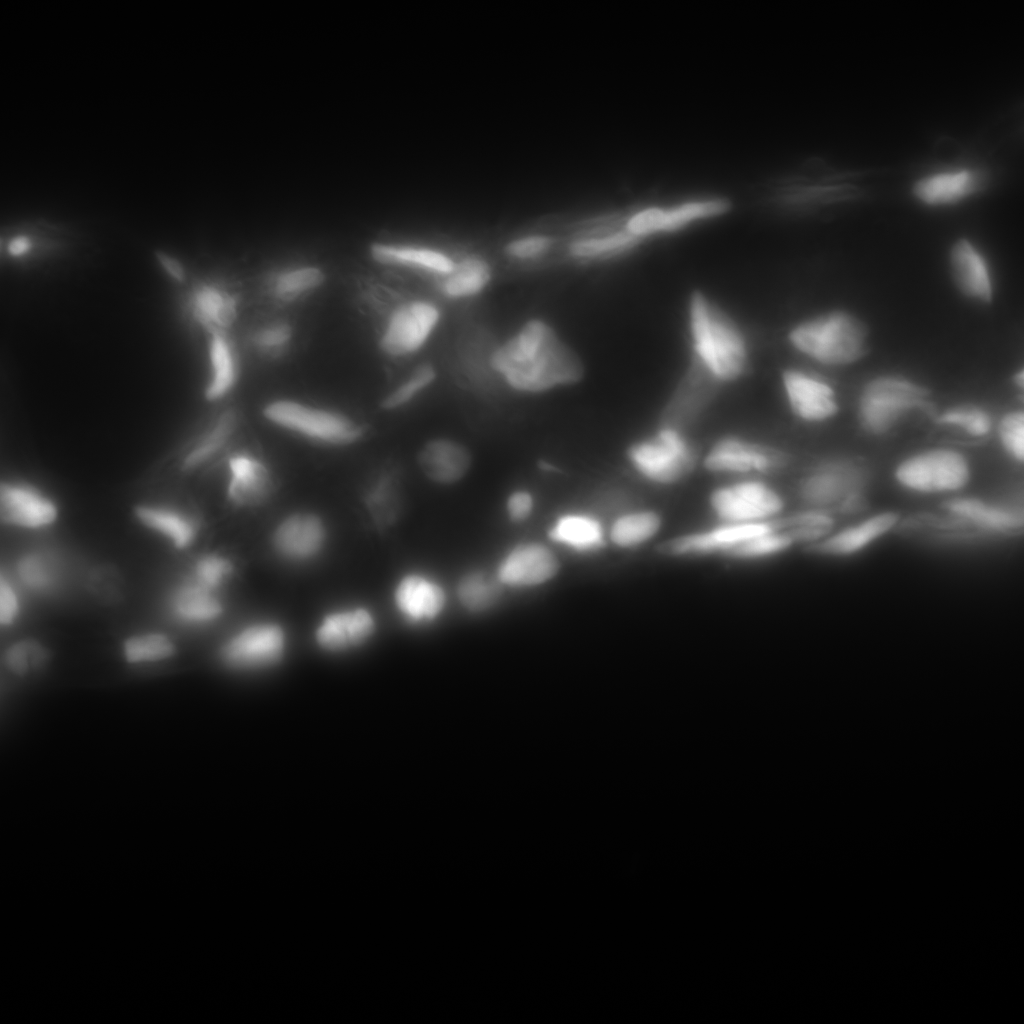

Supplement: Supplementary file 4 — Source data Fig. 2 [file 44318_2025_482_MOESM4_ESM.zip › Fig2 new 3/Fig 2A new 3A/H2AX skin/tert -_- 60x gH2AX skin.tif]

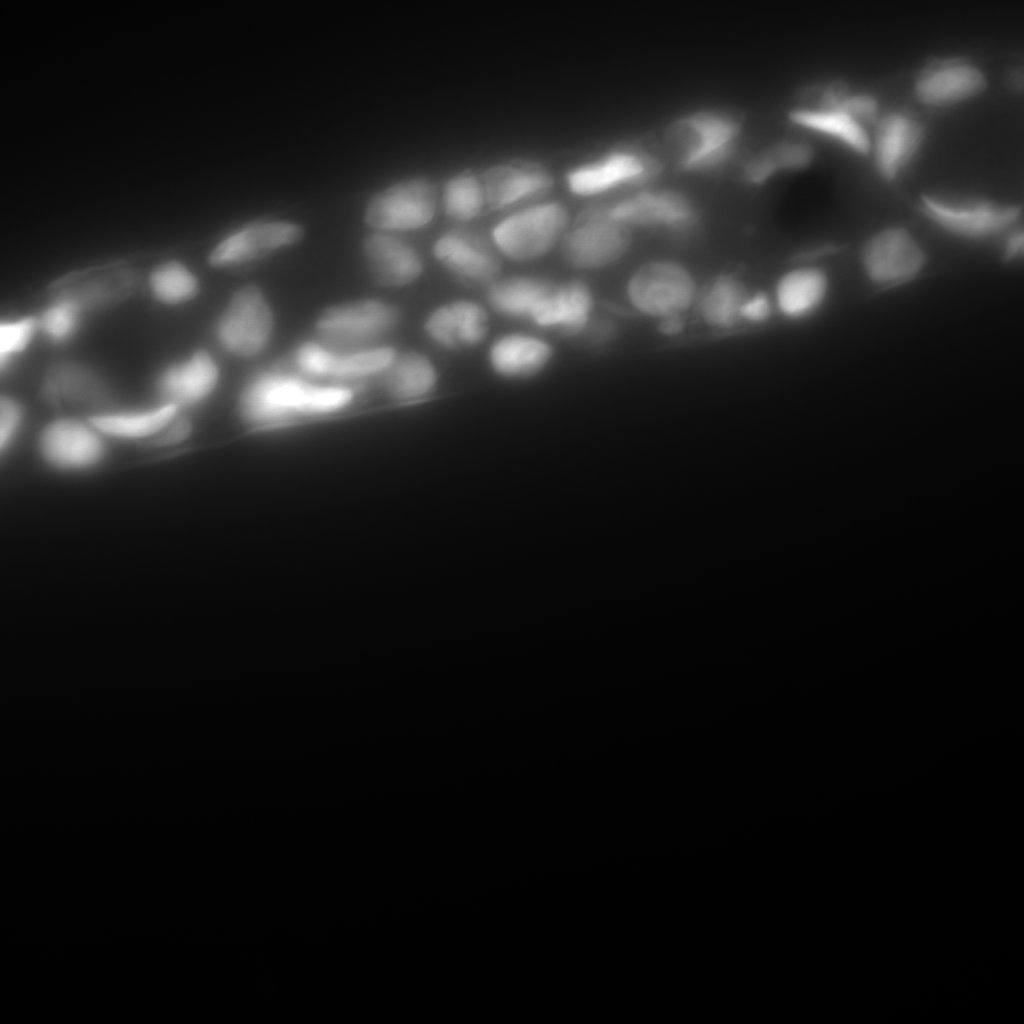

Supplement: Supplementary file 4 — Source data Fig. 2 [file 44318_2025_482_MOESM4_ESM.zip › Fig2 new 3/Fig 2A new 3A/H2AX skin/tert -_- sting-_- 60x gH2AX skin.tif]

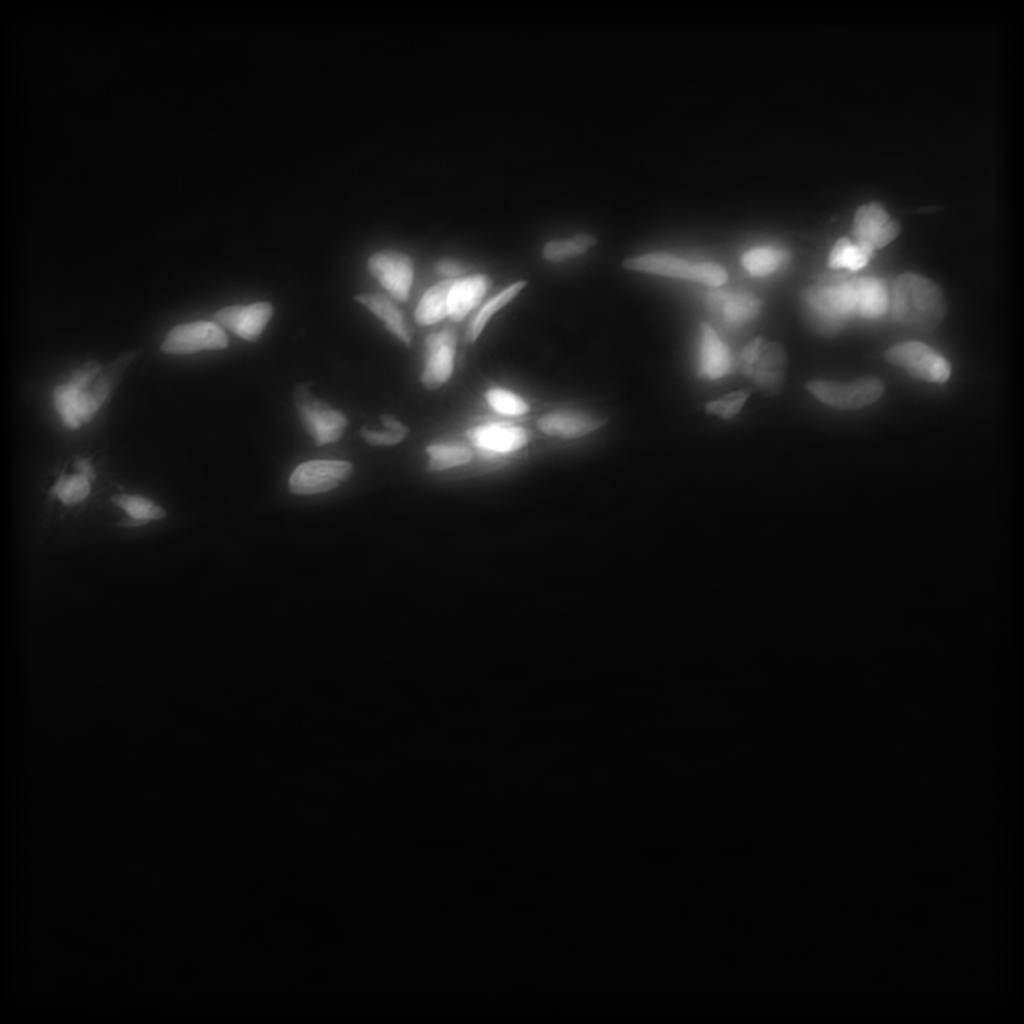

Supplement: Supplementary file 4 — Source data Fig. 2 [file 44318_2025_482_MOESM4_ESM.zip › Fig2 new 3/Fig 2A new 3A/H2AX skin/wt 60X gH2AX skin.tif]

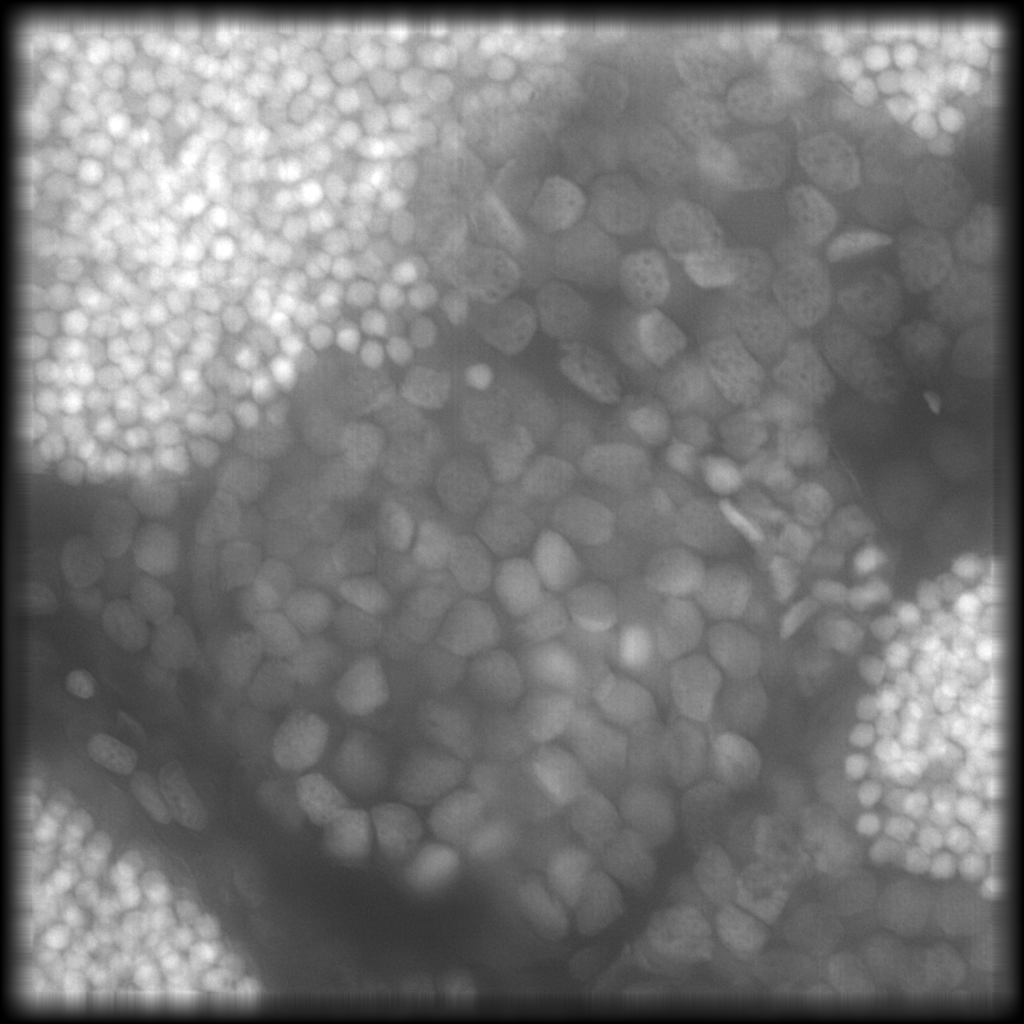

Supplement: Supplementary file 4 — Source data Fig. 2 [file 44318_2025_482_MOESM4_ESM.zip › Fig2 new 3/Fig 2A new 3A/H2AX testis/sting -_- 60x gH2AX testis.tif]

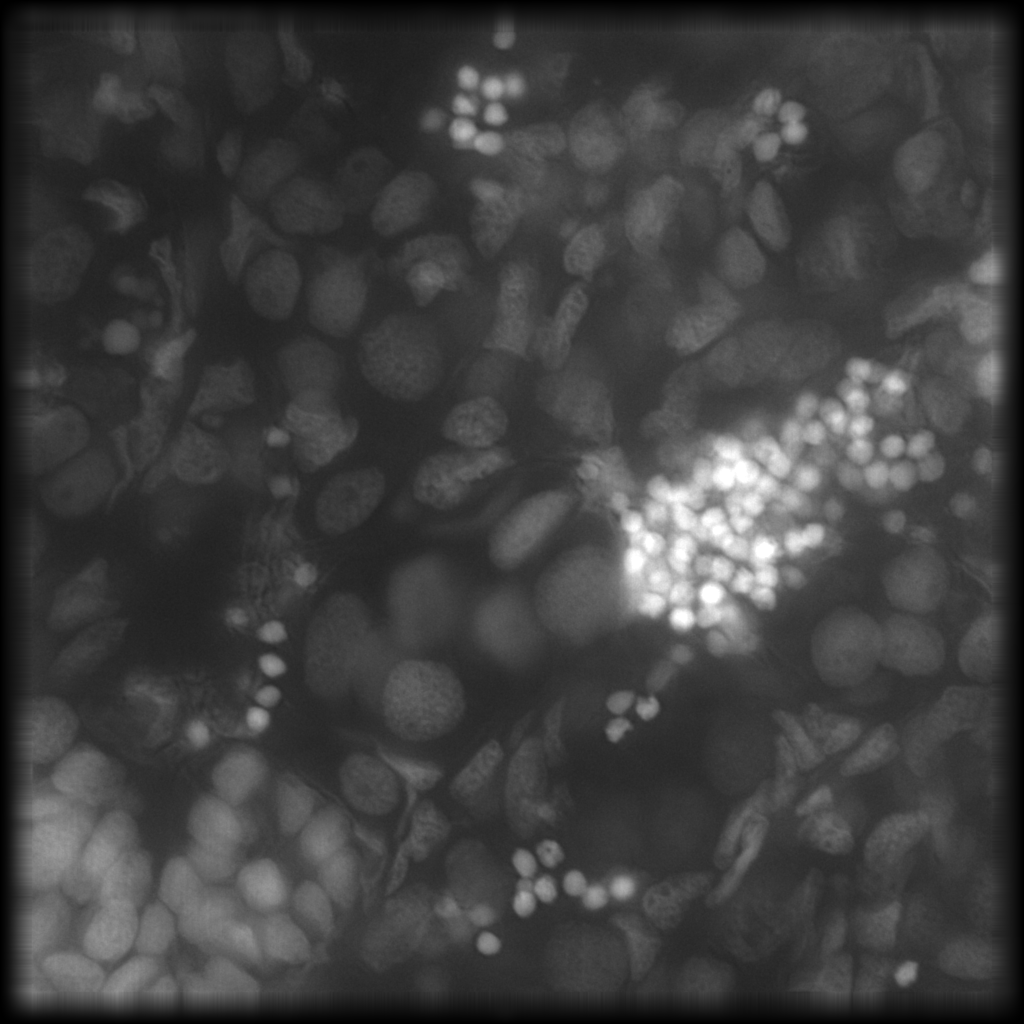

Supplement: Supplementary file 4 — Source data Fig. 2 [file 44318_2025_482_MOESM4_ESM.zip › Fig2 new 3/Fig 2A new 3A/H2AX testis/tert -_- sting-_- 60x gH2AX testis.tif]

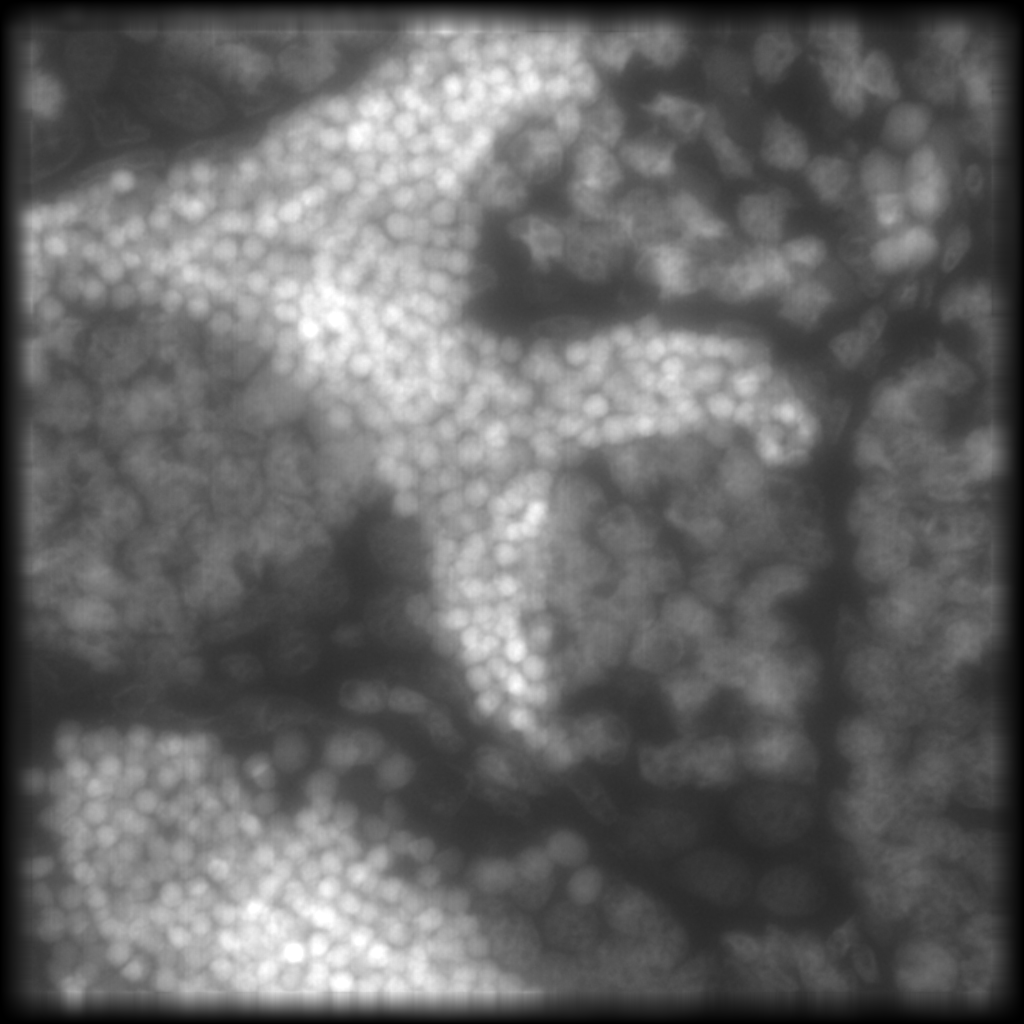

Supplement: Supplementary file 4 — Source data Fig. 2 [file 44318_2025_482_MOESM4_ESM.zip › Fig2 new 3/Fig 2A new 3A/H2AX testis/tert-_- 60x gH2AX testis.tif]

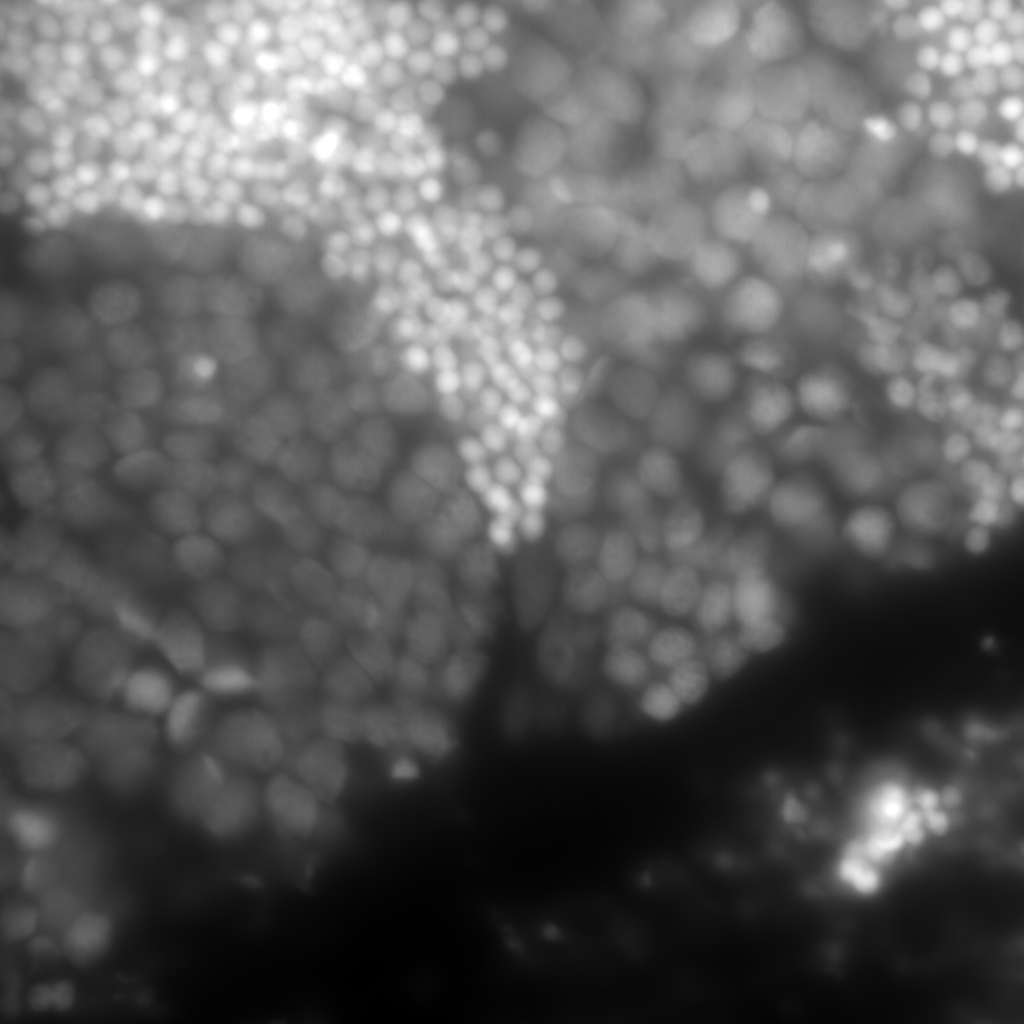

Supplement: Supplementary file 4 — Source data Fig. 2 [file 44318_2025_482_MOESM4_ESM.zip › Fig2 new 3/Fig 2A new 3A/H2AX testis/WT 60x gH2AX testis.tif]

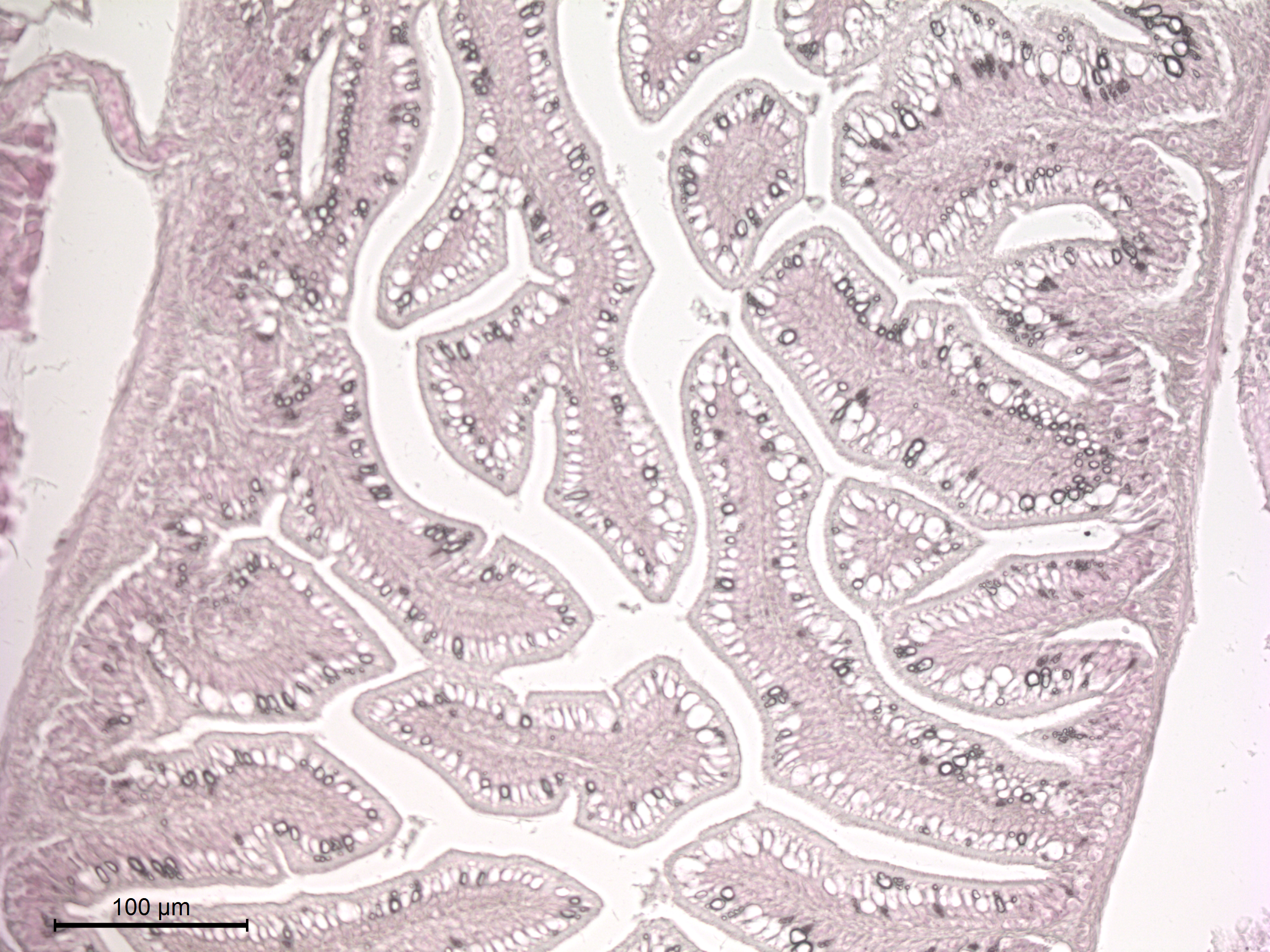

Supplement: Supplementary file 5 — Source data Fig. 3 [file 44318_2025_482_MOESM5_ESM.zip › Fig3 new 4/Fig 3A new 4A/intestine/20x intestine STING.tif]

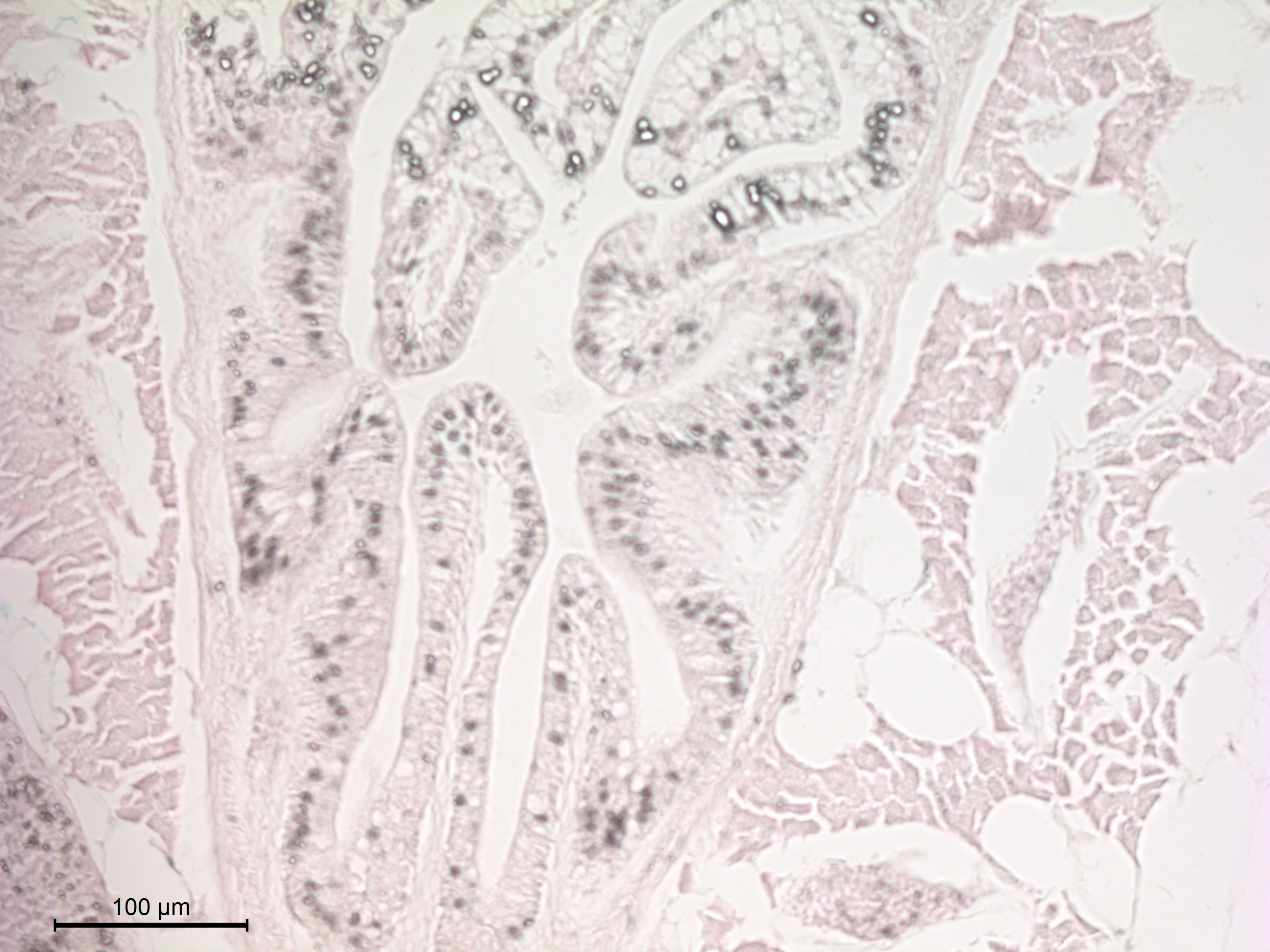

Supplement: Supplementary file 5 — Source data Fig. 3 [file 44318_2025_482_MOESM5_ESM.zip › Fig3 new 4/Fig 3A new 4A/intestine/20x intestine TERT STING.tif]

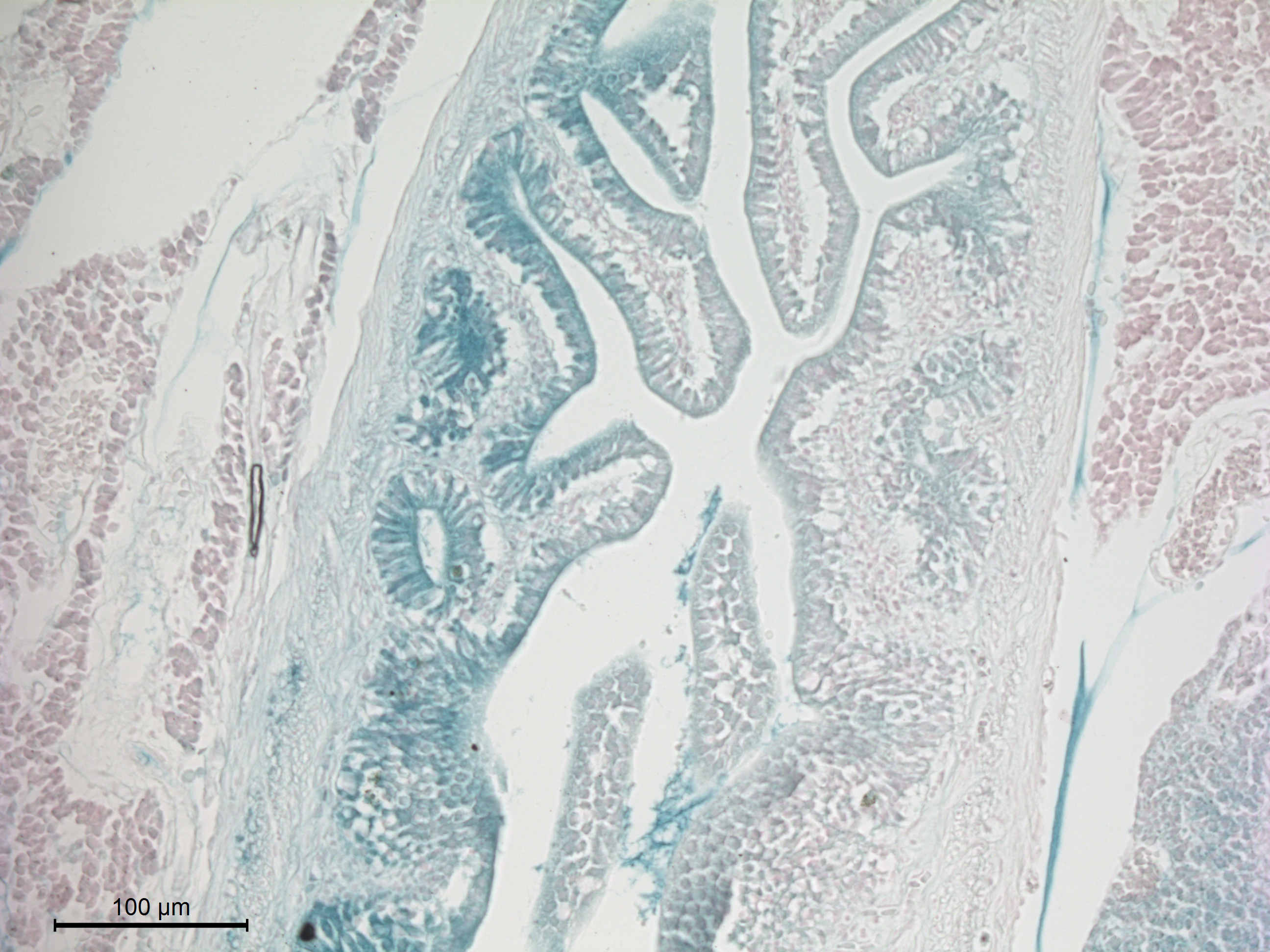

Supplement: Supplementary file 5 — Source data Fig. 3 [file 44318_2025_482_MOESM5_ESM.zip › Fig3 new 4/Fig 3A new 4A/intestine/20x intestine TERT.tif]

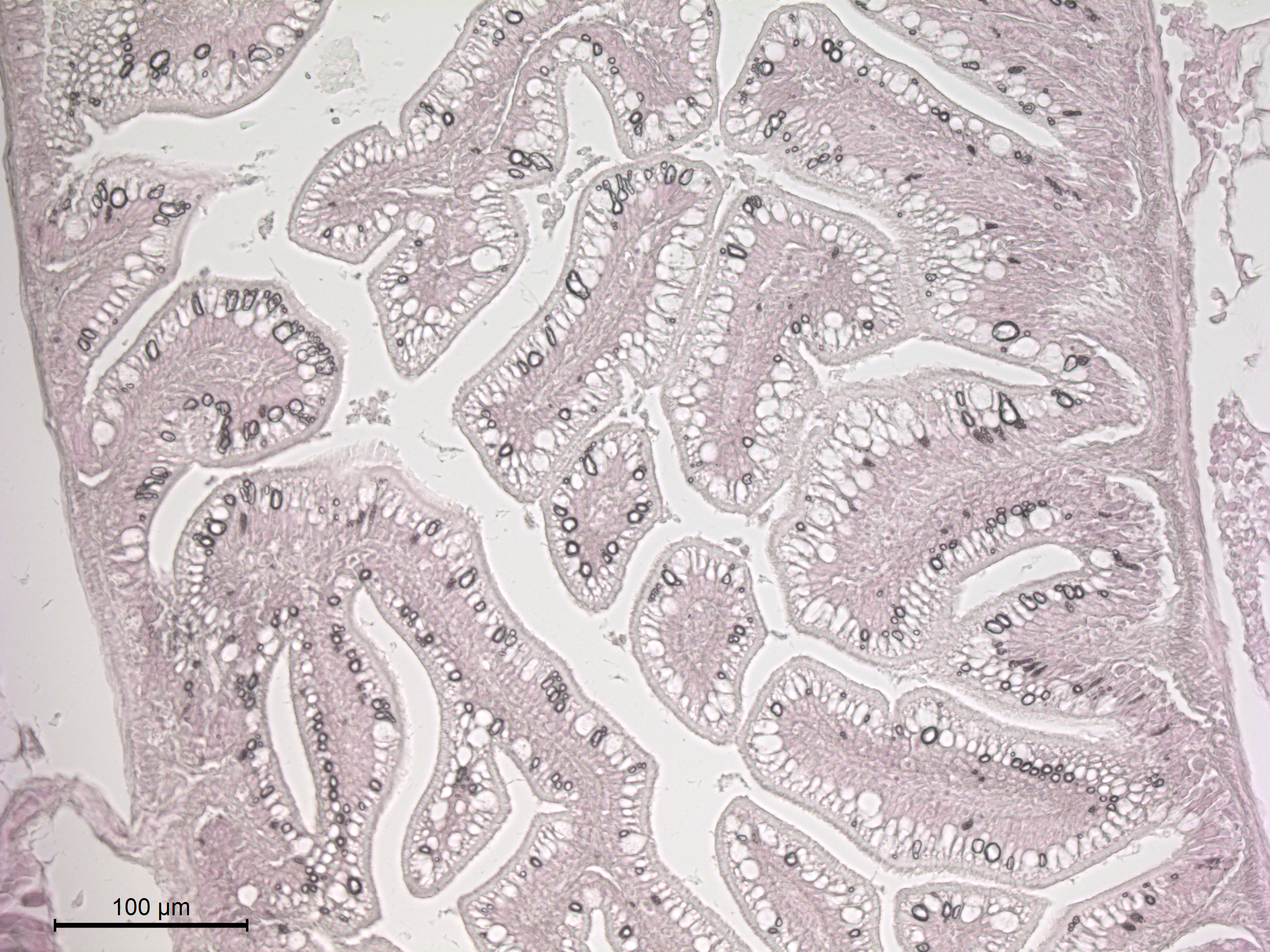

Supplement: Supplementary file 5 — Source data Fig. 3 [file 44318_2025_482_MOESM5_ESM.zip › Fig3 new 4/Fig 3A new 4A/intestine/20x intestine WT.tif]

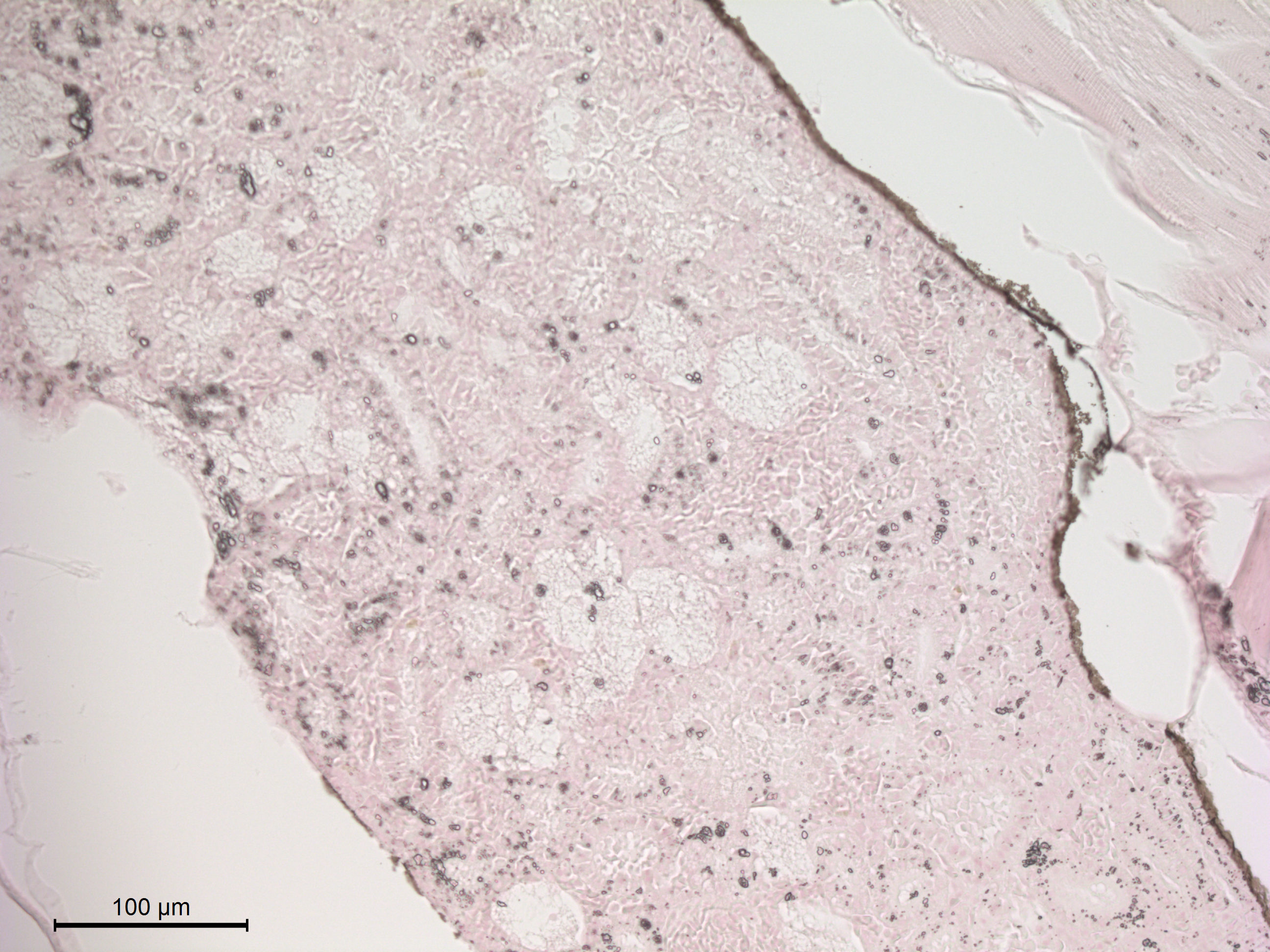

Supplement: Supplementary file 5 — Source data Fig. 3 [file 44318_2025_482_MOESM5_ESM.zip › Fig3 new 4/Fig 3A new 4A/Kidney marrow/20x kidney marrow STING.tif]

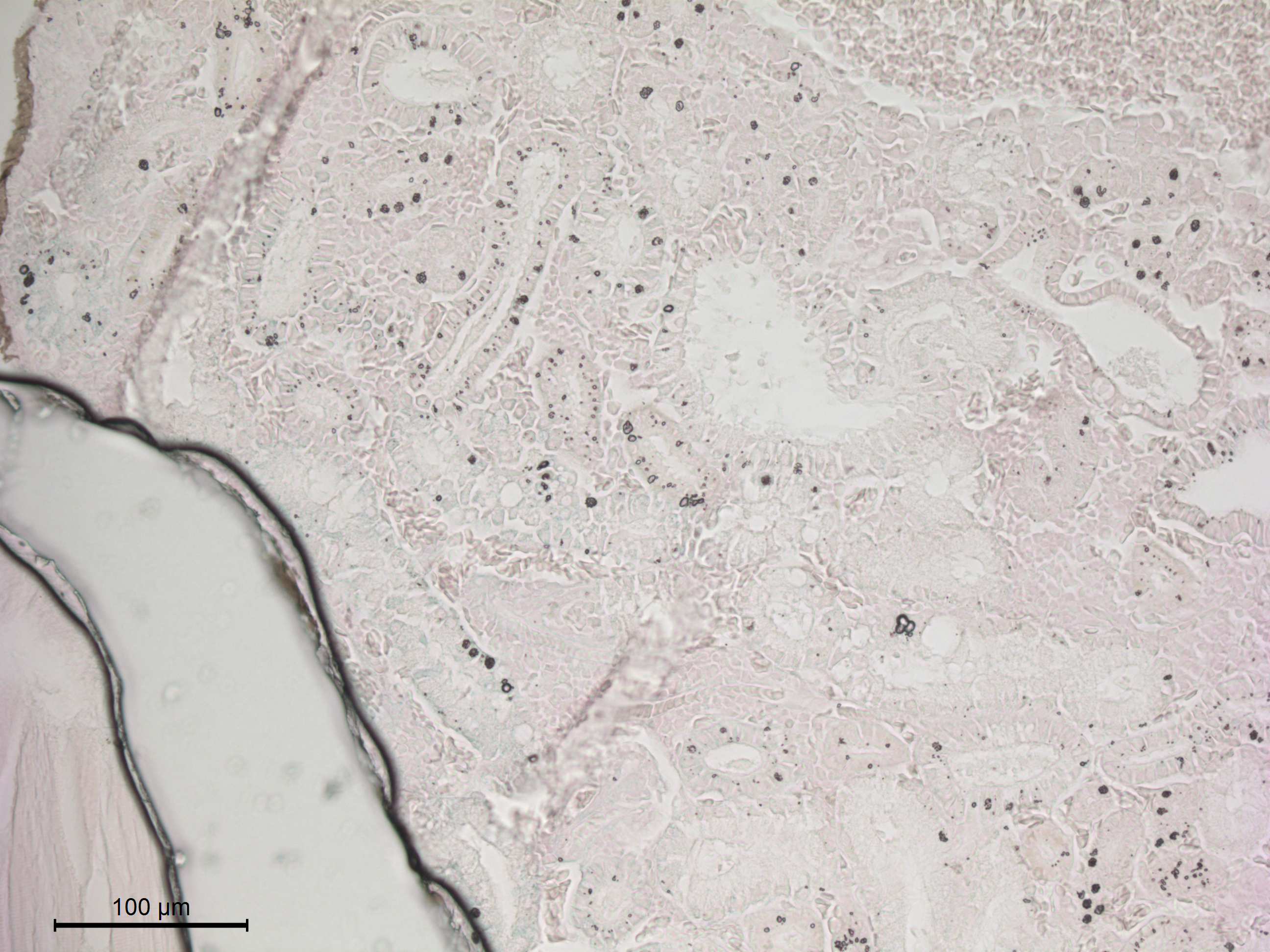

Supplement: Supplementary file 5 — Source data Fig. 3 [file 44318_2025_482_MOESM5_ESM.zip › Fig3 new 4/Fig 3A new 4A/Kidney marrow/20x kidney marrow TERT STING.tif]

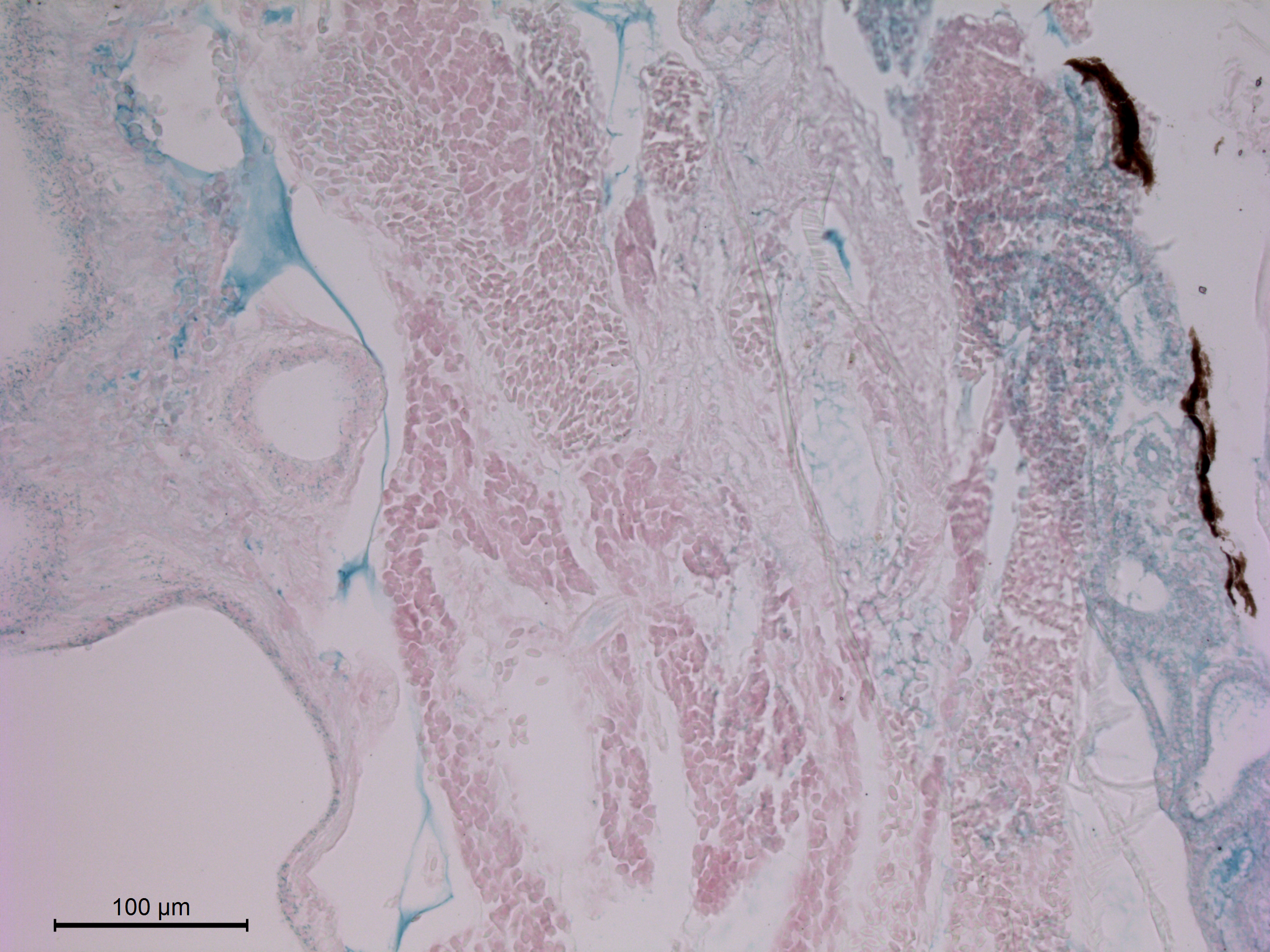

Supplement: Supplementary file 5 — Source data Fig. 3 [file 44318_2025_482_MOESM5_ESM.zip › Fig3 new 4/Fig 3A new 4A/Kidney marrow/20X kidney marrow TERT.tif]

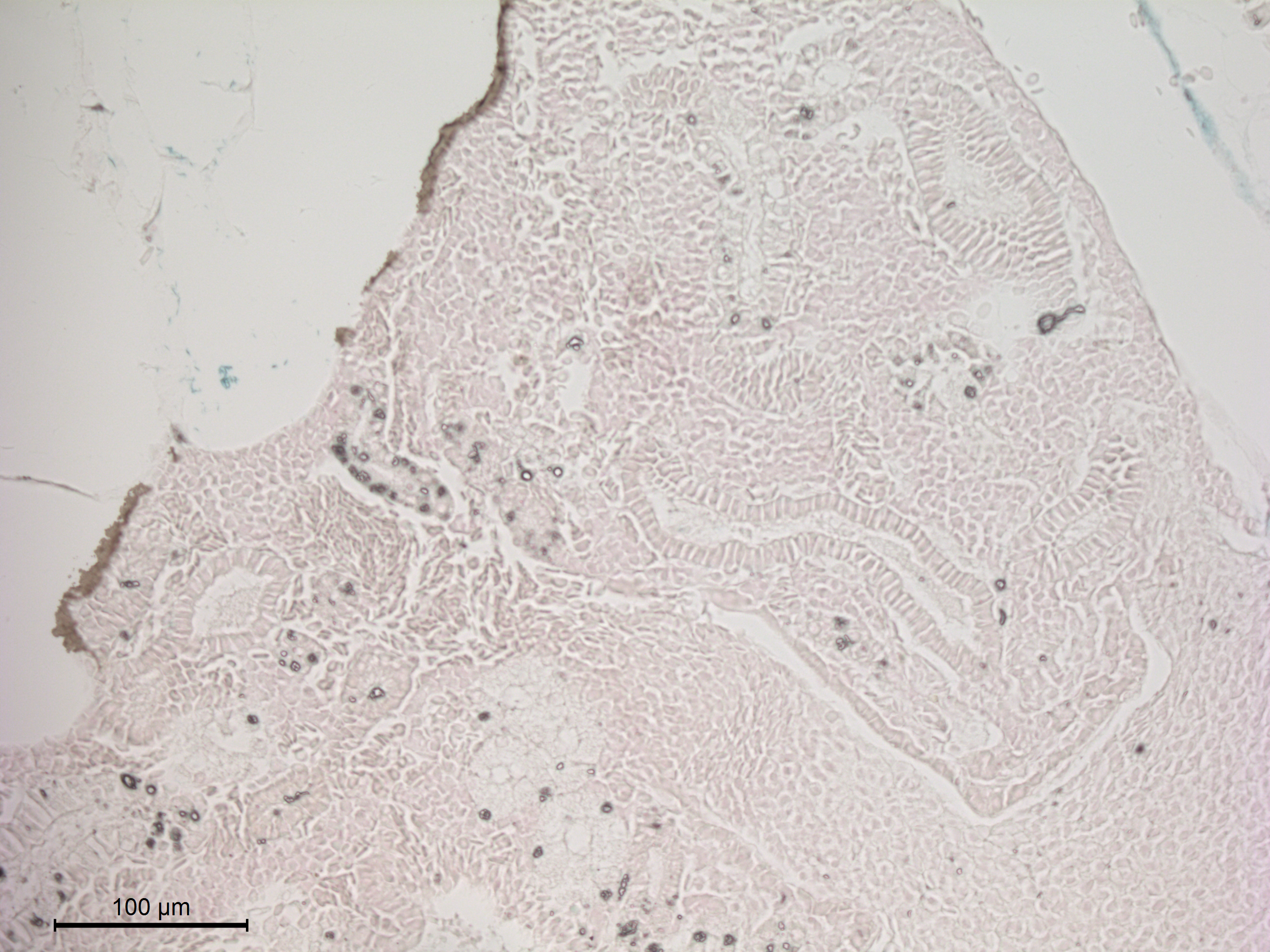

Supplement: Supplementary file 5 — Source data Fig. 3 [file 44318_2025_482_MOESM5_ESM.zip › Fig3 new 4/Fig 3A new 4A/Kidney marrow/20x kidney marrow WT.tif]

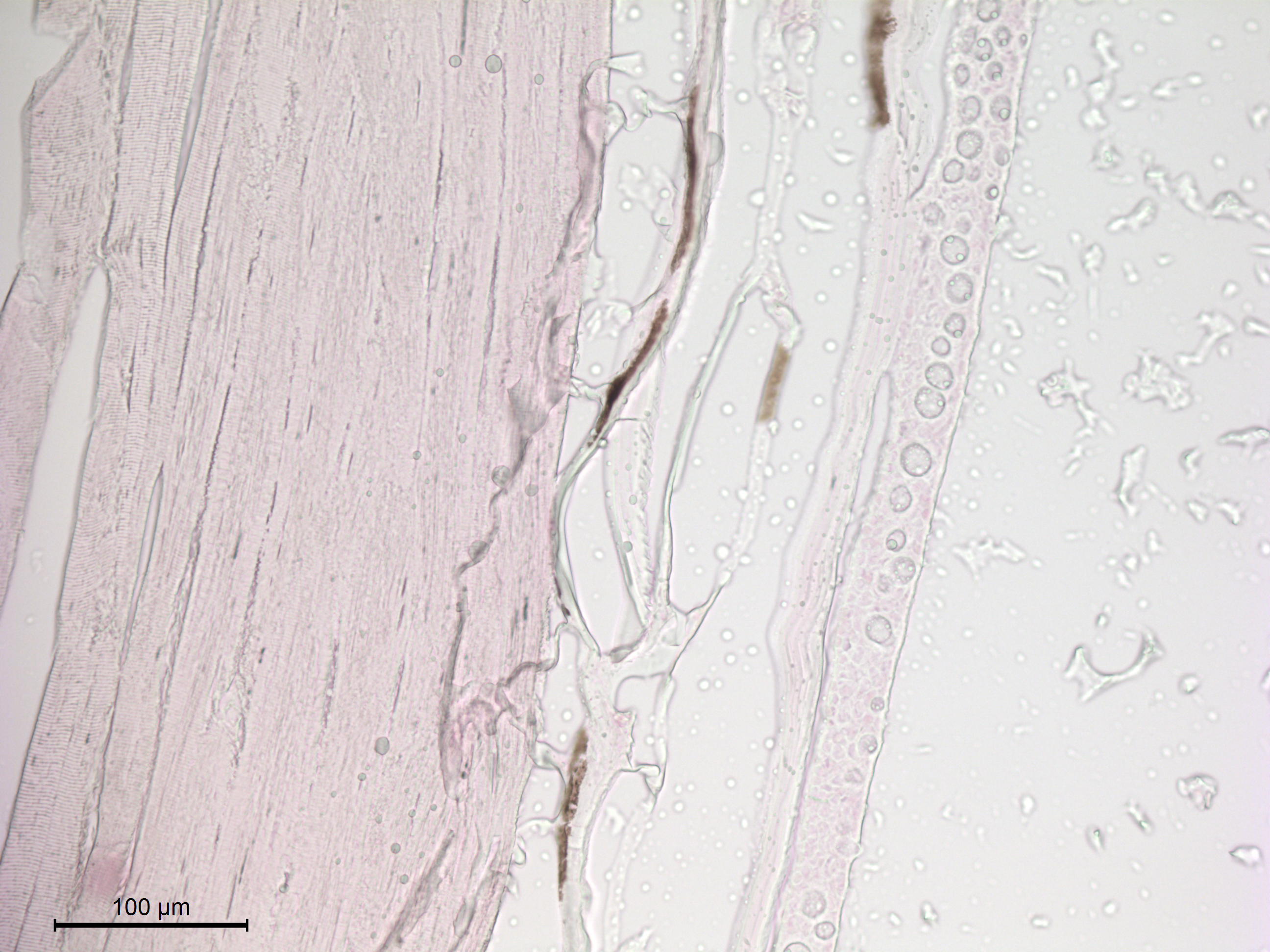

Supplement: Supplementary file 5 — Source data Fig. 3 [file 44318_2025_482_MOESM5_ESM.zip › Fig3 new 4/Fig 3A new 4A/skin/20x skin STING.tif]

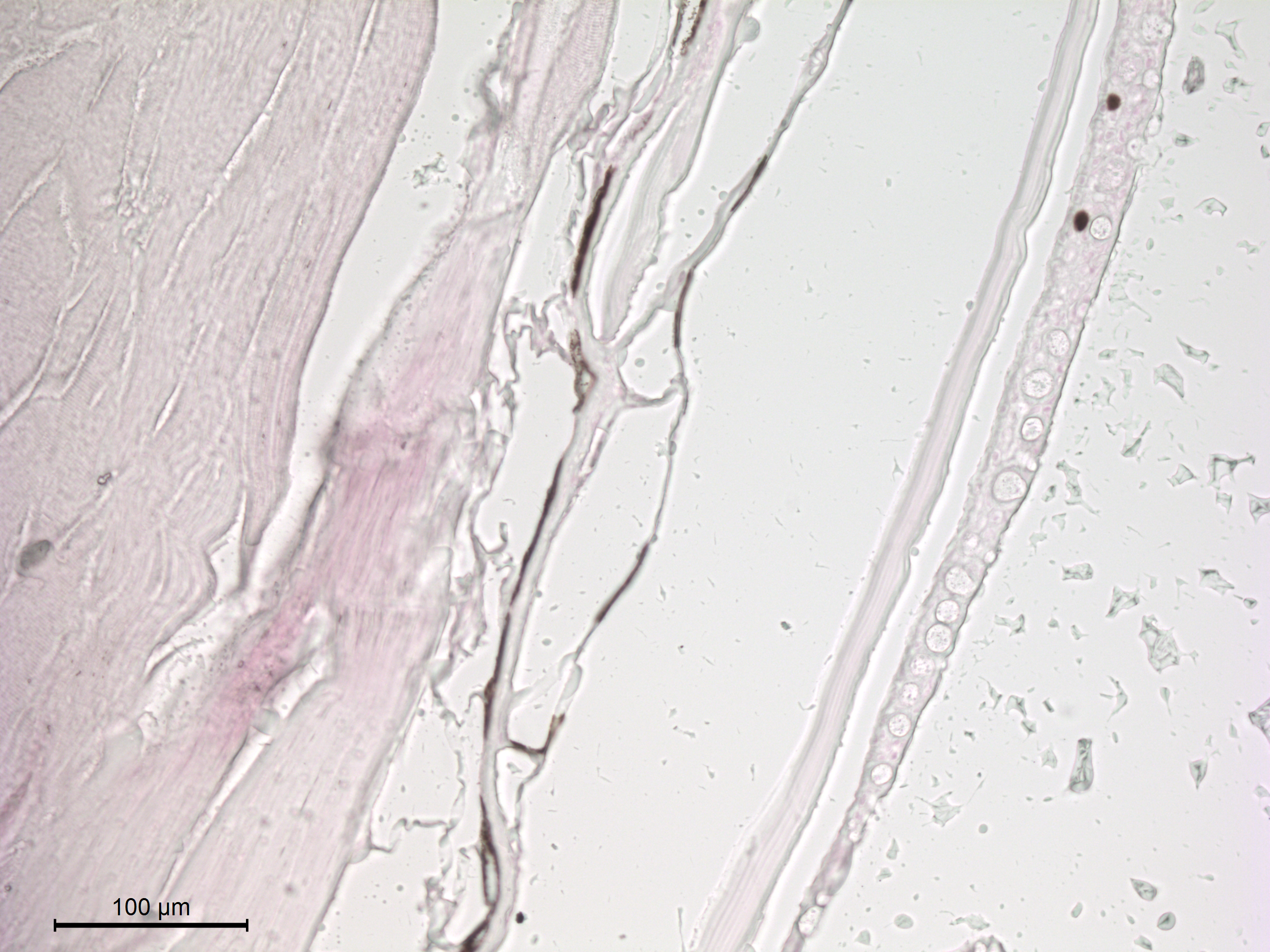

Supplement: Supplementary file 5 — Source data Fig. 3 [file 44318_2025_482_MOESM5_ESM.zip › Fig3 new 4/Fig 3A new 4A/skin/20x skin TERT STING.tif]

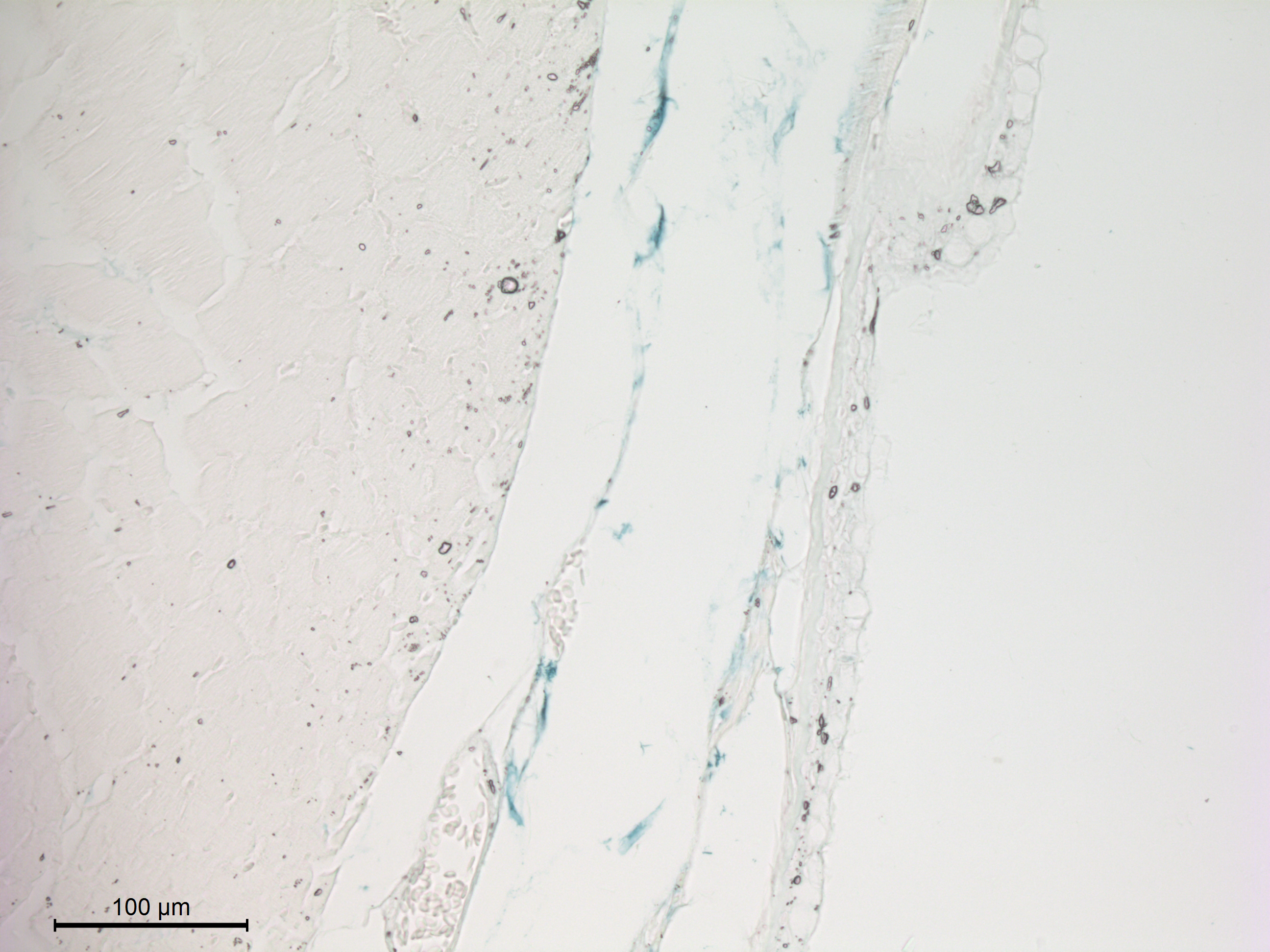

Supplement: Supplementary file 5 — Source data Fig. 3 [file 44318_2025_482_MOESM5_ESM.zip › Fig3 new 4/Fig 3A new 4A/skin/20x skin TERT.tif]

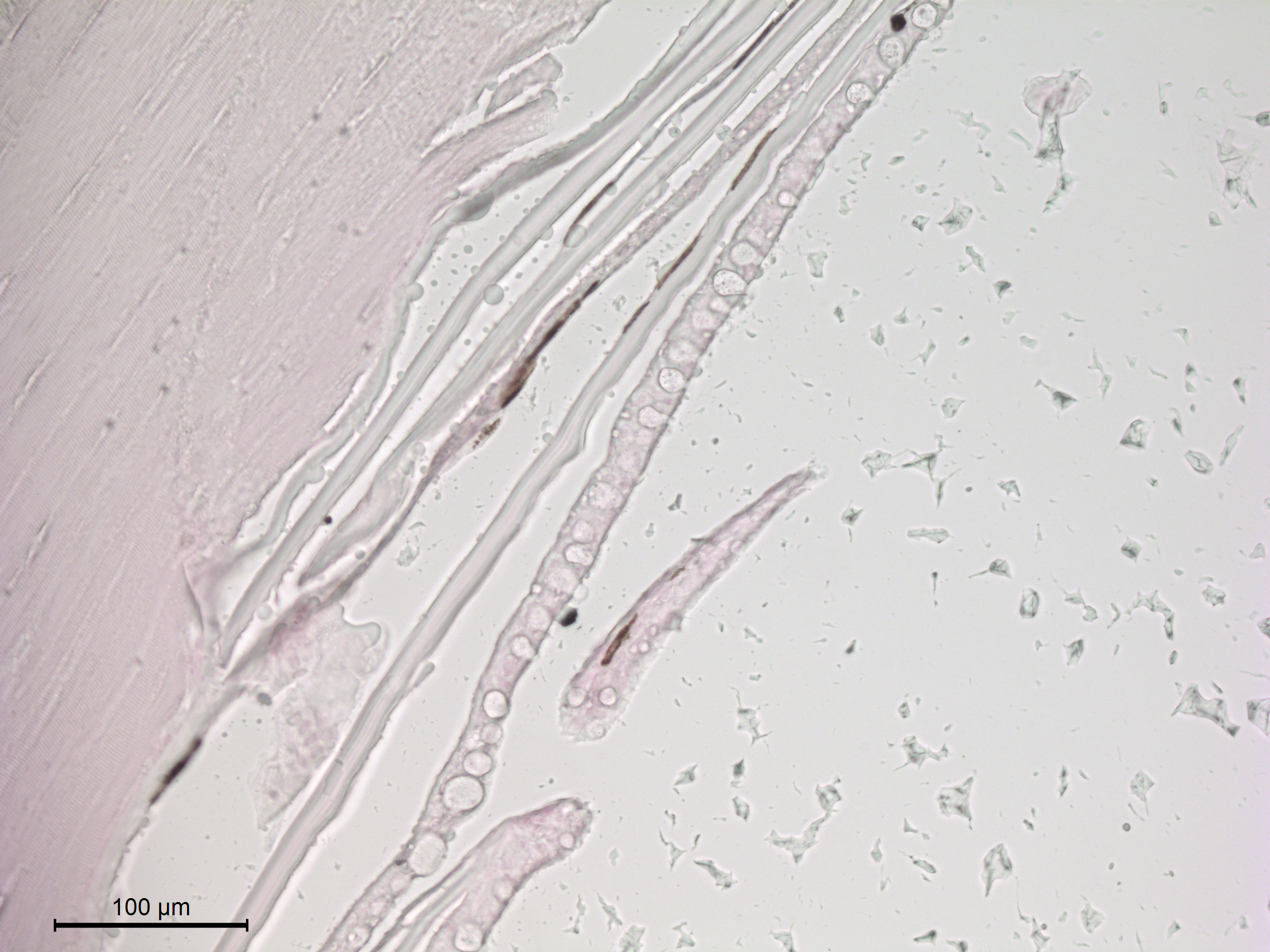

Supplement: Supplementary file 5 — Source data Fig. 3 [file 44318_2025_482_MOESM5_ESM.zip › Fig3 new 4/Fig 3A new 4A/skin/20x skin WT.tif]

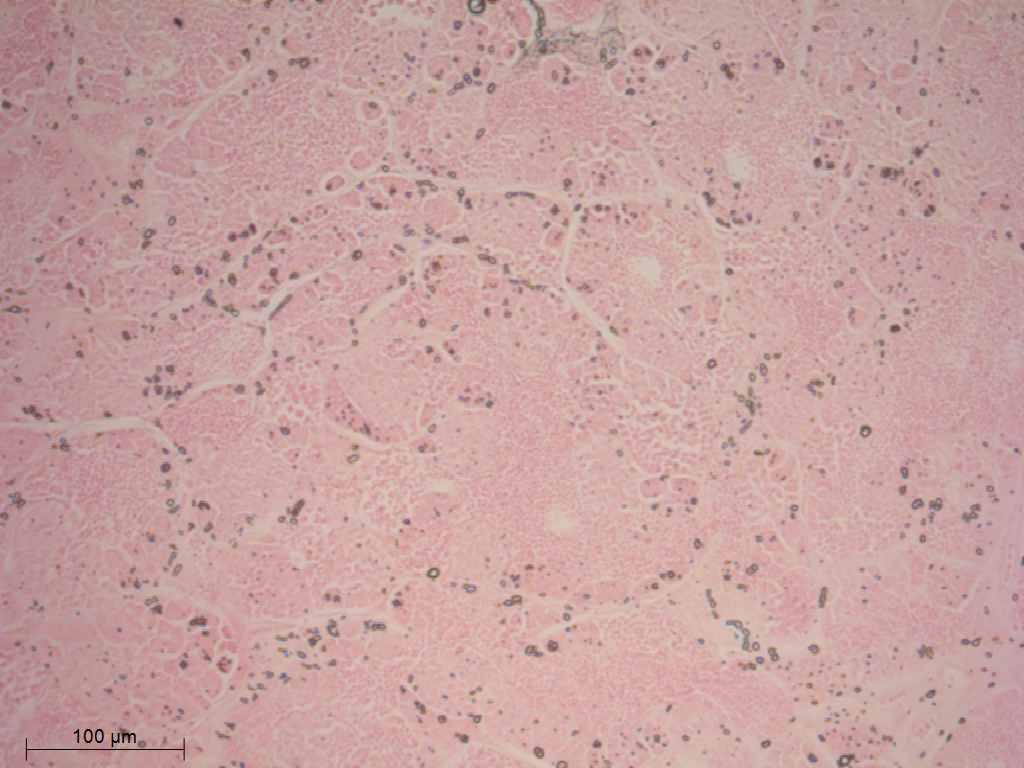

Supplement: Supplementary file 5 — Source data Fig. 3 [file 44318_2025_482_MOESM5_ESM.zip › Fig3 new 4/Fig 3A new 4A/testis/testis sting.tif]

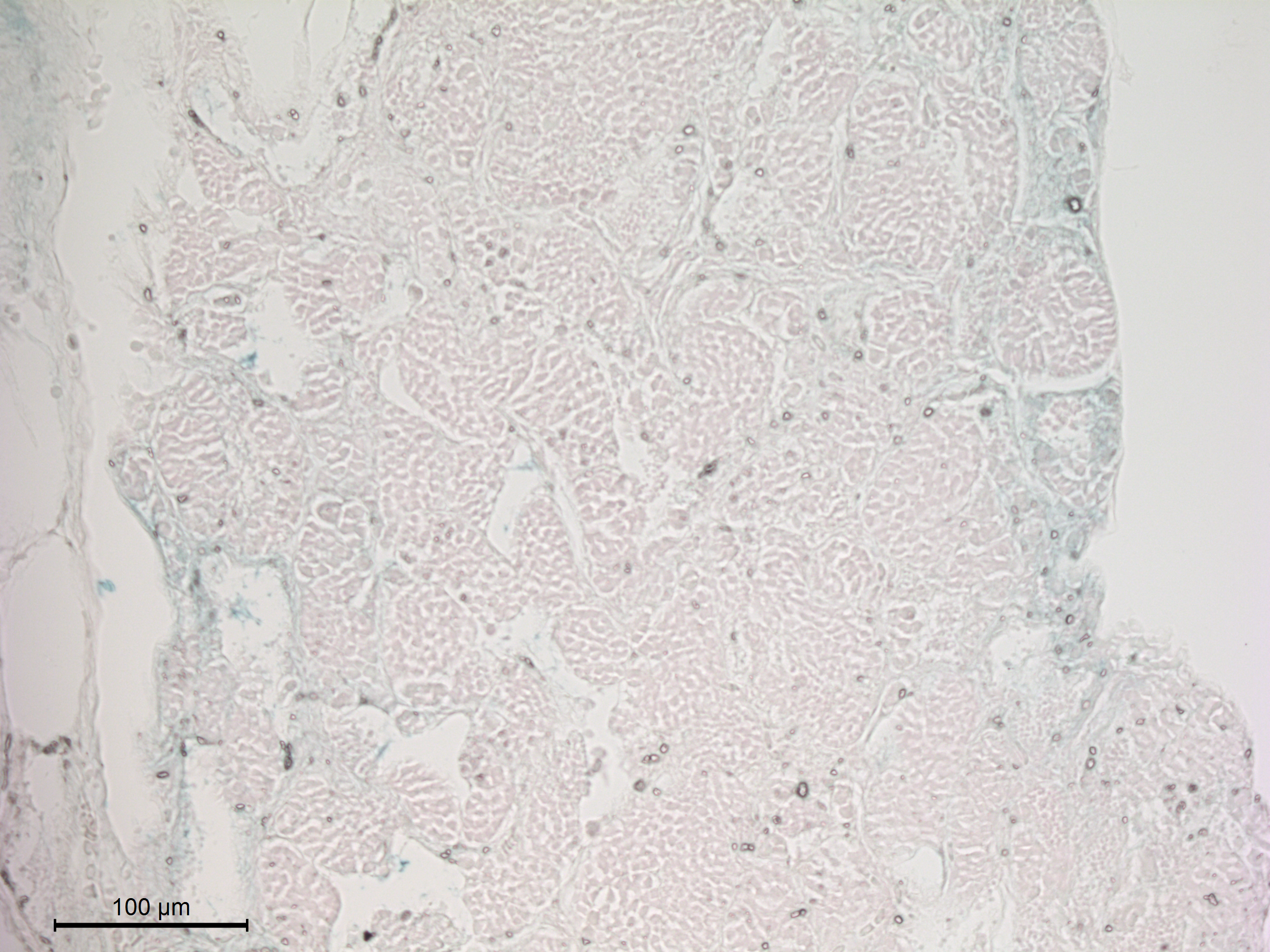

Supplement: Supplementary file 5 — Source data Fig. 3 [file 44318_2025_482_MOESM5_ESM.zip › Fig3 new 4/Fig 3A new 4A/testis/testis TERT.tif]

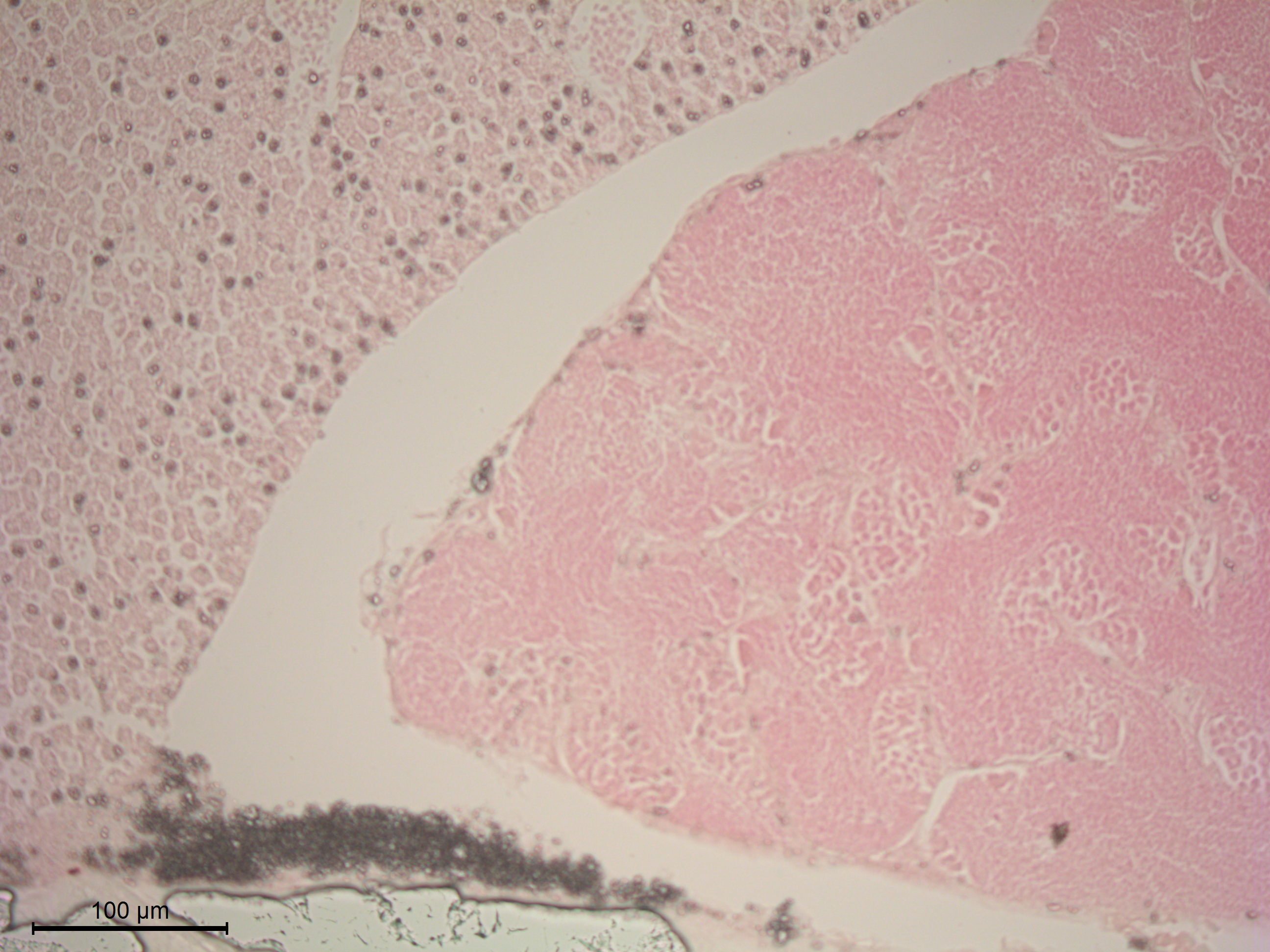

Supplement: Supplementary file 5 — Source data Fig. 3 [file 44318_2025_482_MOESM5_ESM.zip › Fig3 new 4/Fig 3A new 4A/testis/testis tertsting.tif]

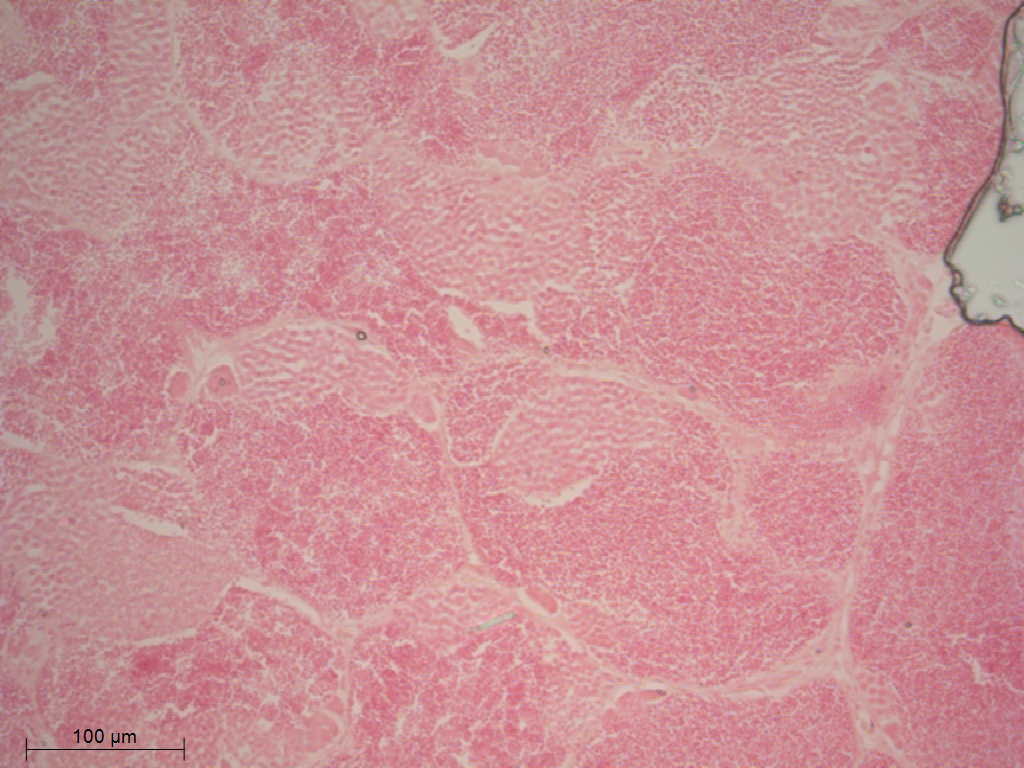

Supplement: Supplementary file 5 — Source data Fig. 3 [file 44318_2025_482_MOESM5_ESM.zip › Fig3 new 4/Fig 3A new 4A/testis/testis WT.tif]

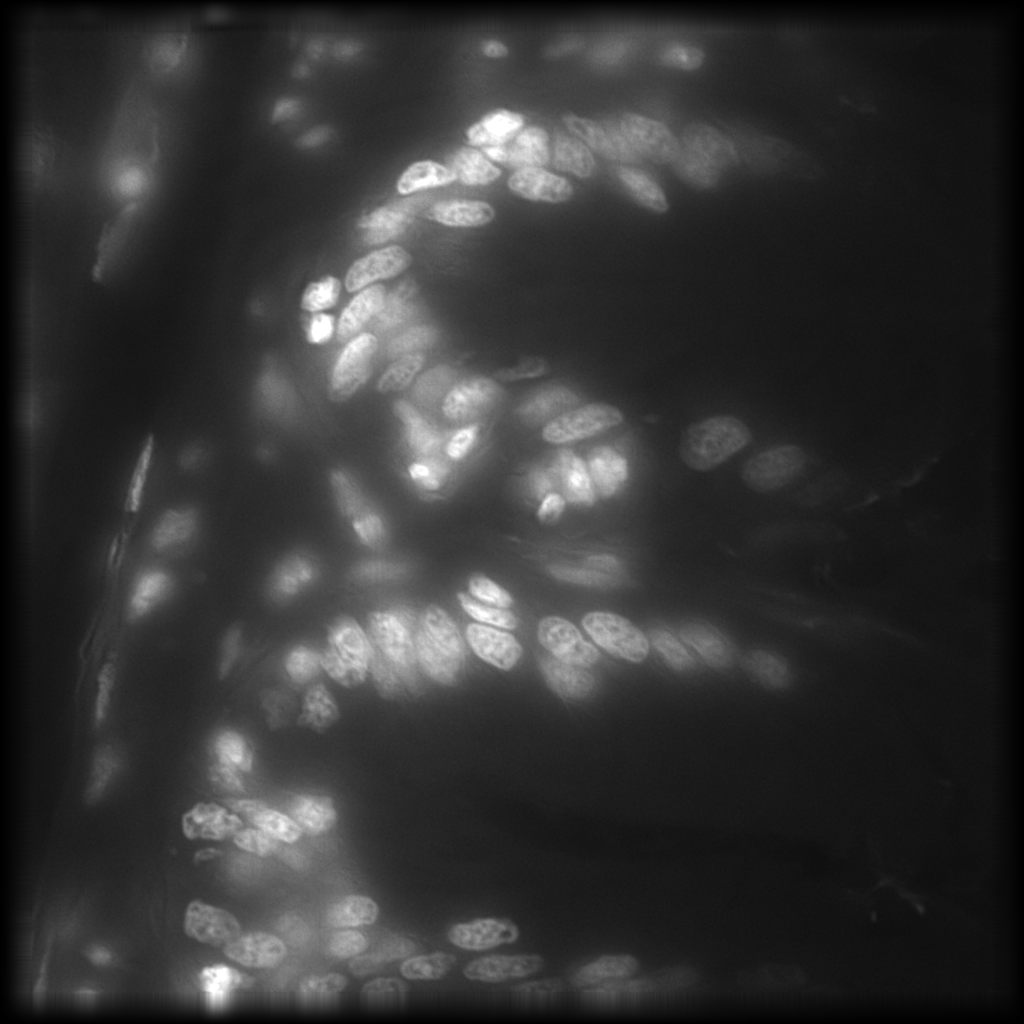

Supplement: Supplementary file 6 — Source data Fig. 4 [file 44318_2025_482_MOESM6_ESM.zip › Fig4 new 5/Fig 4A new 5A/PCNA intestine/sting -_- 60x PCNA intestine.tif]

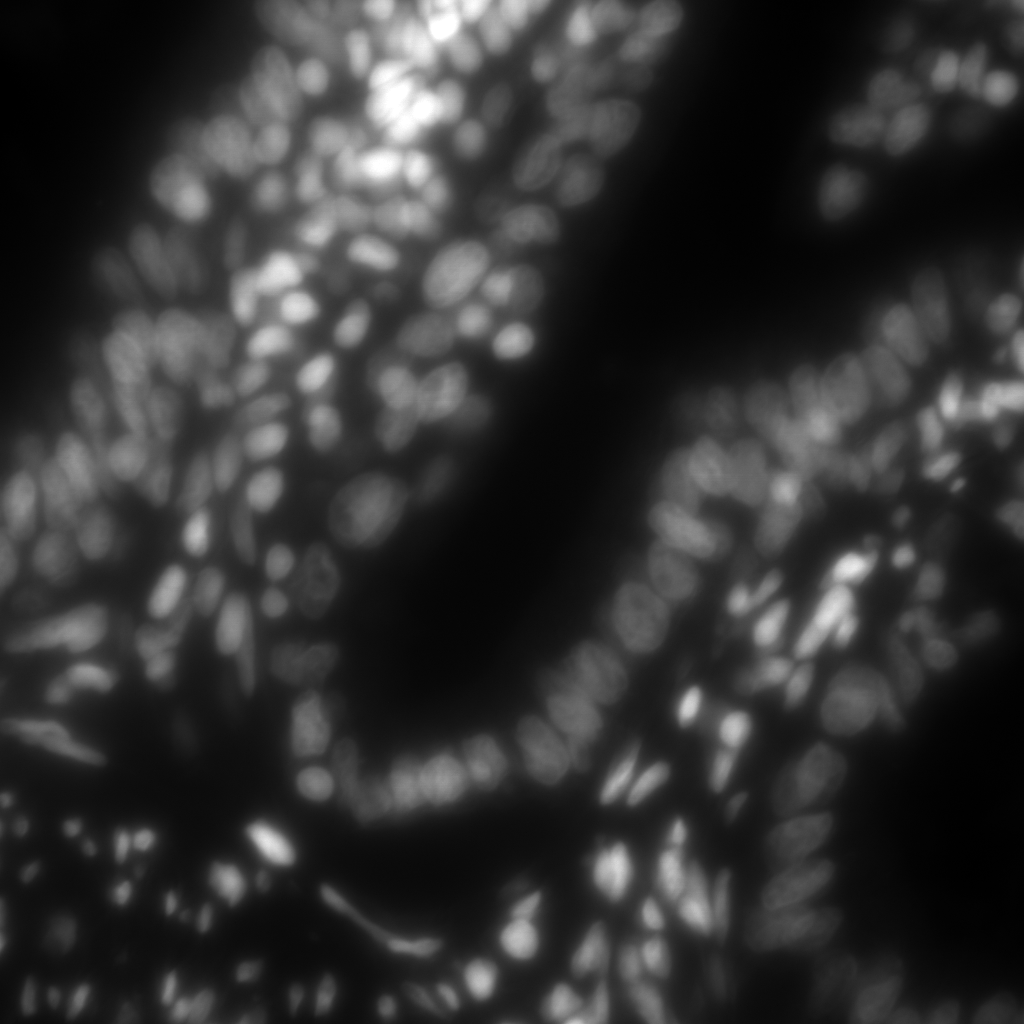

Supplement: Supplementary file 6 — Source data Fig. 4 [file 44318_2025_482_MOESM6_ESM.zip › Fig4 new 5/Fig 4A new 5A/PCNA intestine/tert -_- PCNA 60x intestine.tif]

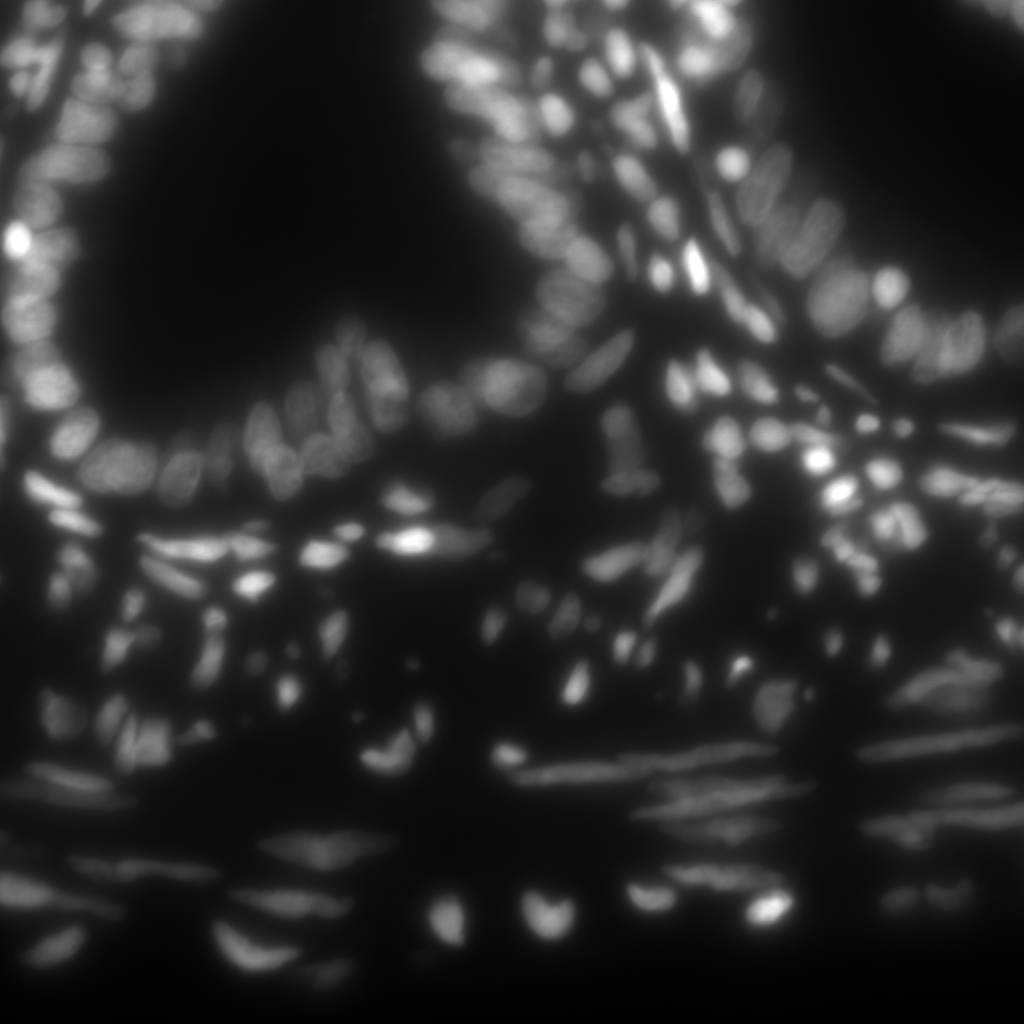

Supplement: Supplementary file 6 — Source data Fig. 4 [file 44318_2025_482_MOESM6_ESM.zip › Fig4 new 5/Fig 4A new 5A/PCNA intestine/tert -_- sting -_- 60x PCNA intestine.tif]

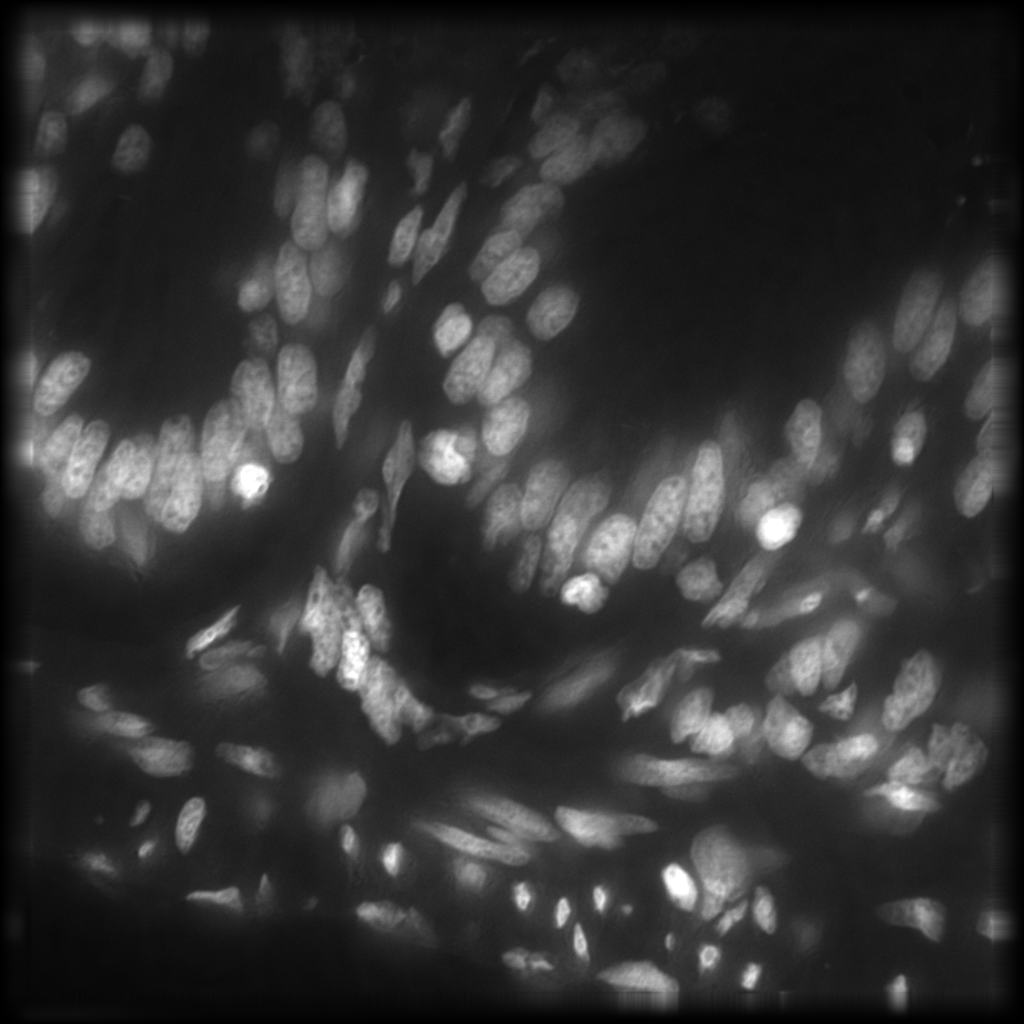

Supplement: Supplementary file 6 — Source data Fig. 4 [file 44318_2025_482_MOESM6_ESM.zip › Fig4 new 5/Fig 4A new 5A/PCNA intestine/wt -_- PCNA 60x intestine.tif]

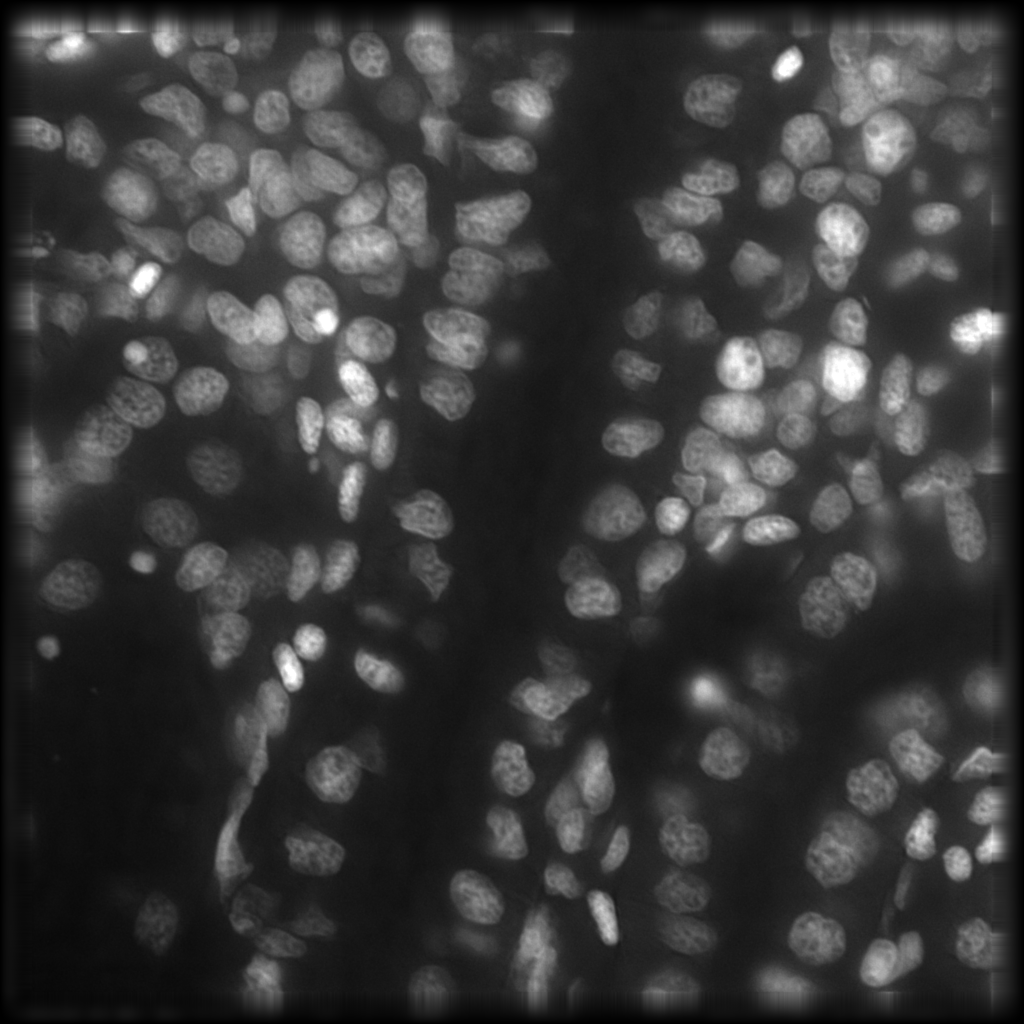

Supplement: Supplementary file 6 — Source data Fig. 4 [file 44318_2025_482_MOESM6_ESM.zip › Fig4 new 5/Fig 4A new 5A/PCNA KM/sting-_- 60x PCNA kidney marrow.tif]

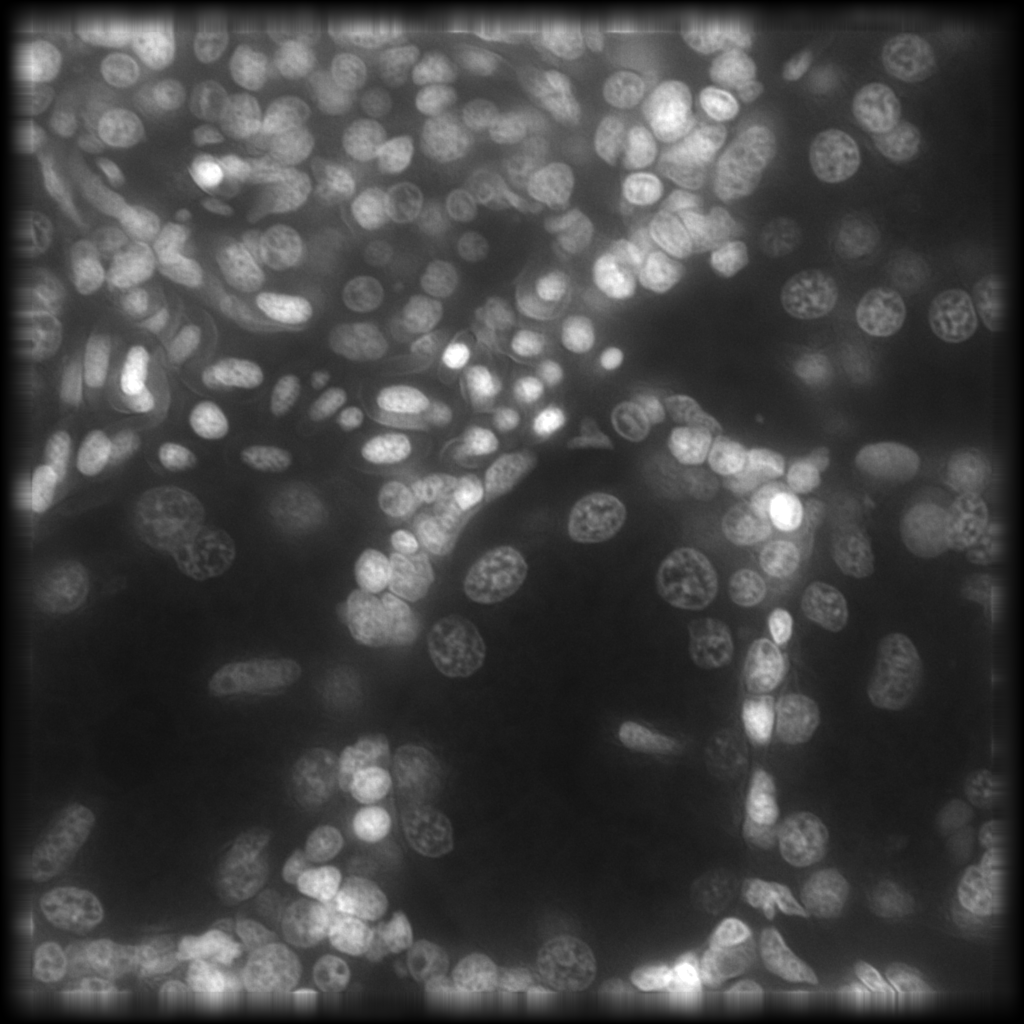

Supplement: Supplementary file 6 — Source data Fig. 4 [file 44318_2025_482_MOESM6_ESM.zip › Fig4 new 5/Fig 4A new 5A/PCNA KM/tert -_- 60x PCNA kidney marrow.tif]

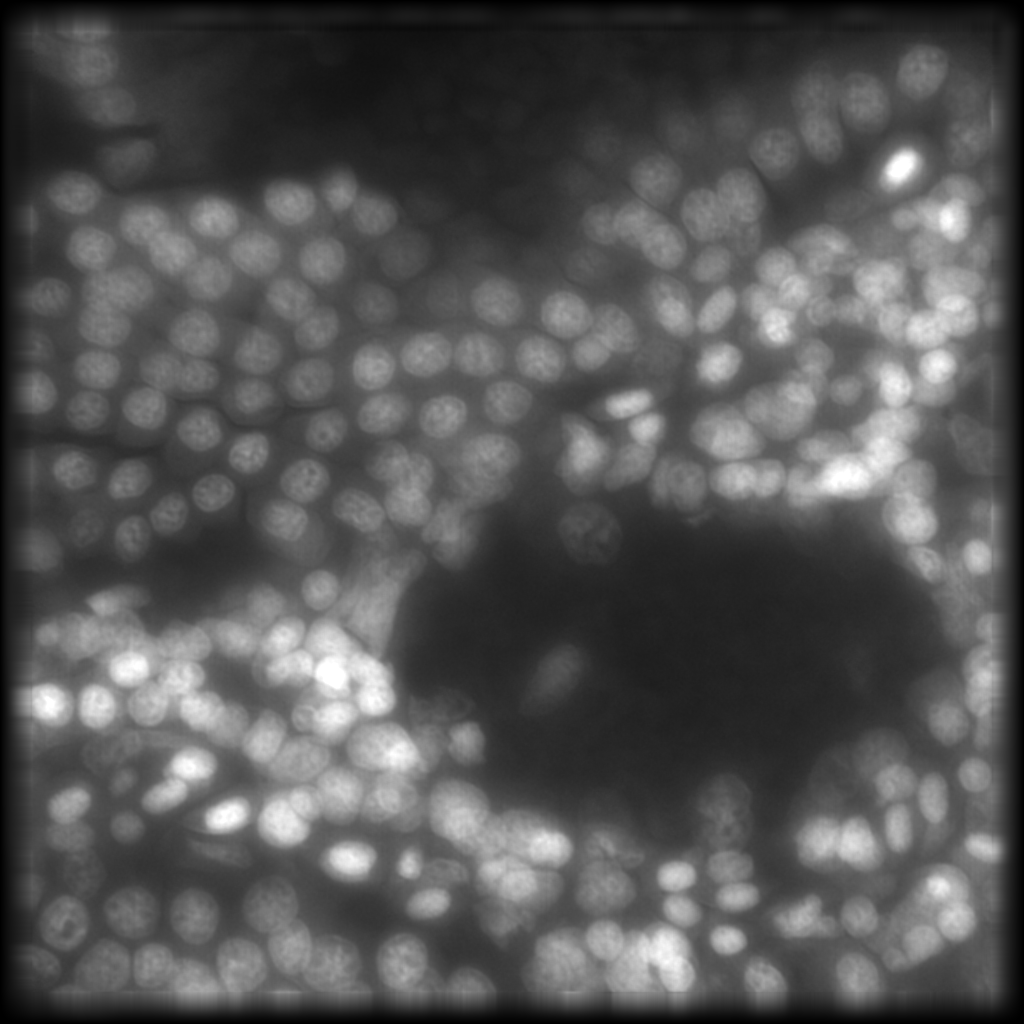

Supplement: Supplementary file 6 — Source data Fig. 4 [file 44318_2025_482_MOESM6_ESM.zip › Fig4 new 5/Fig 4A new 5A/PCNA KM/tert -_- sting -_- 60x PCNA head kidney.tif]

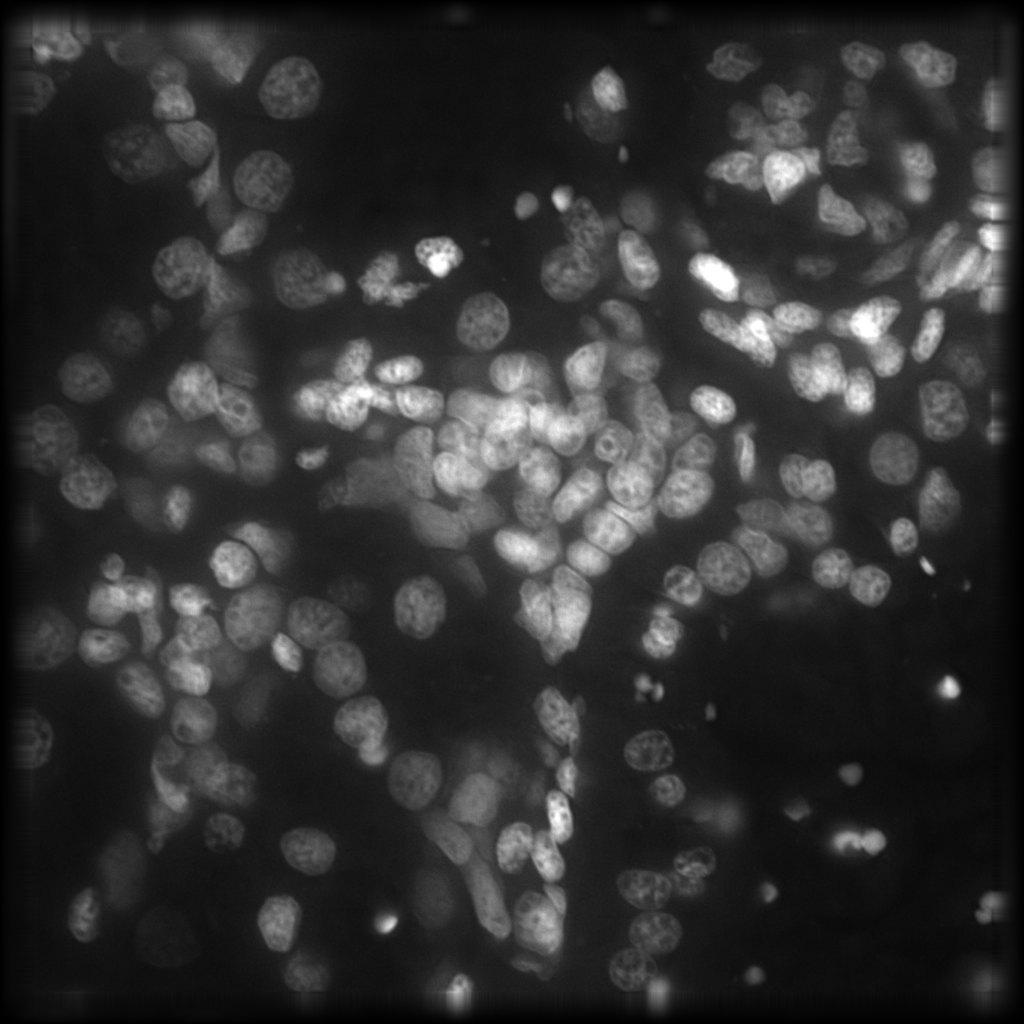

Supplement: Supplementary file 6 — Source data Fig. 4 [file 44318_2025_482_MOESM6_ESM.zip › Fig4 new 5/Fig 4A new 5A/PCNA KM/wt 60x PCNA Kidney Marrow.tif]

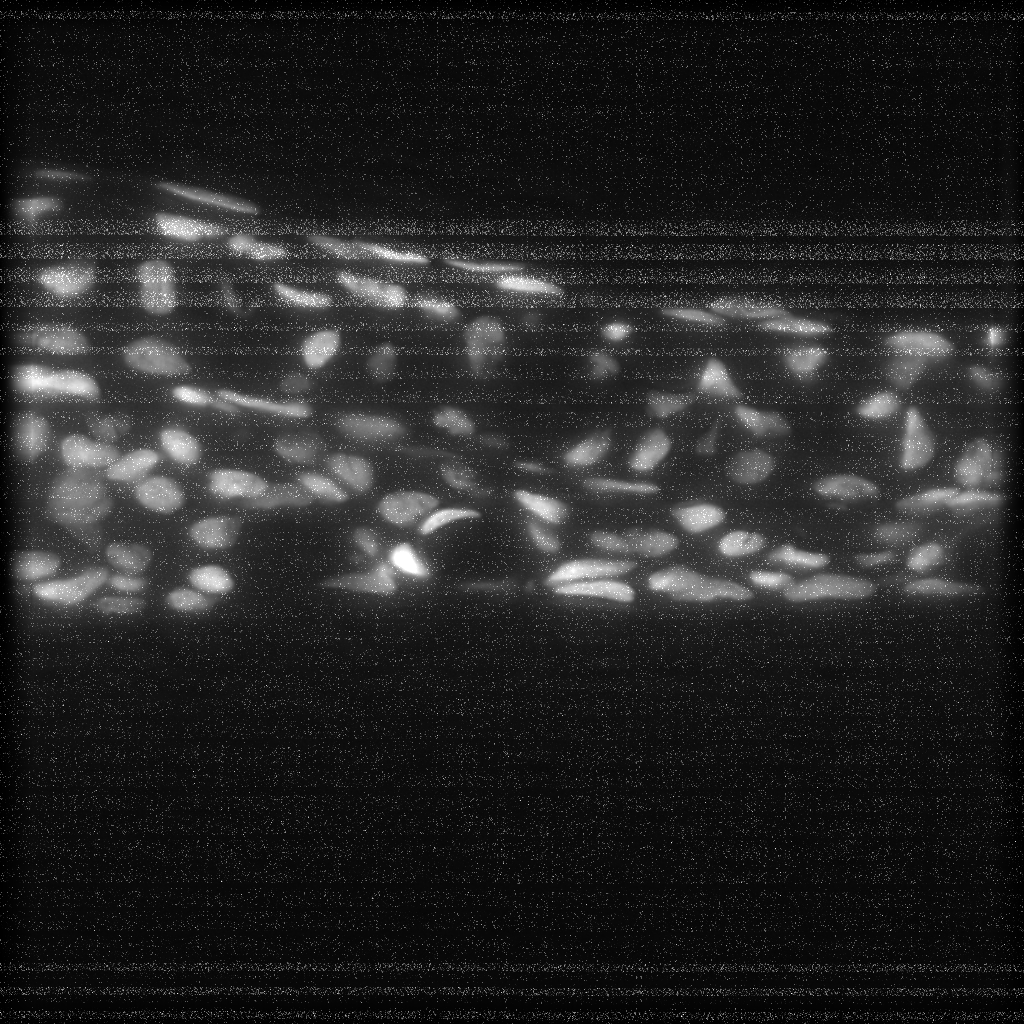

Supplement: Supplementary file 6 — Source data Fig. 4 [file 44318_2025_482_MOESM6_ESM.zip › Fig4 new 5/Fig 4A new 5A/PCNA skin/sting -_- 60x PCNA skin.tif]

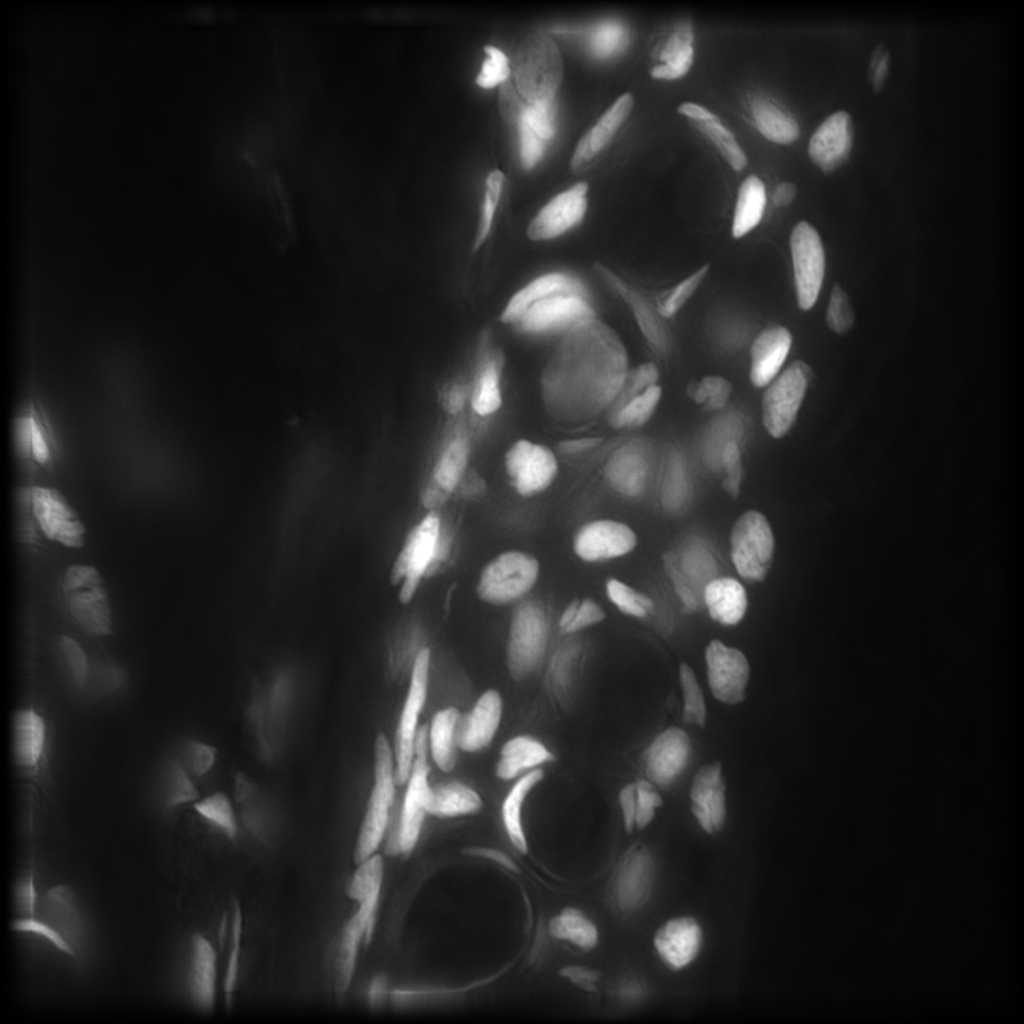

Supplement: Supplementary file 6 — Source data Fig. 4 [file 44318_2025_482_MOESM6_ESM.zip › Fig4 new 5/Fig 4A new 5A/PCNA skin/tert-_- 60x PCNA skin.tif]

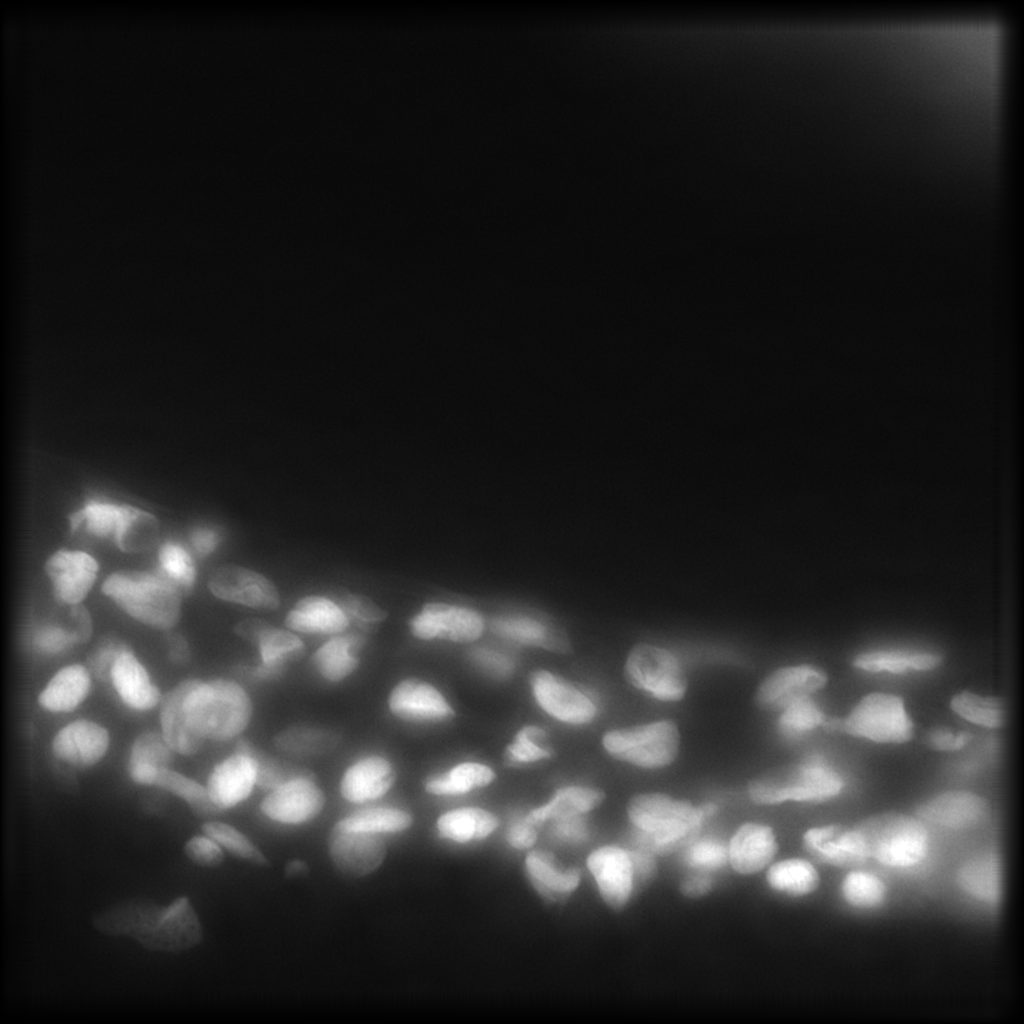

Supplement: Supplementary file 6 — Source data Fig. 4 [file 44318_2025_482_MOESM6_ESM.zip › Fig4 new 5/Fig 4A new 5A/PCNA skin/tert-_- sting-_- 60X PCNA skin.tif]

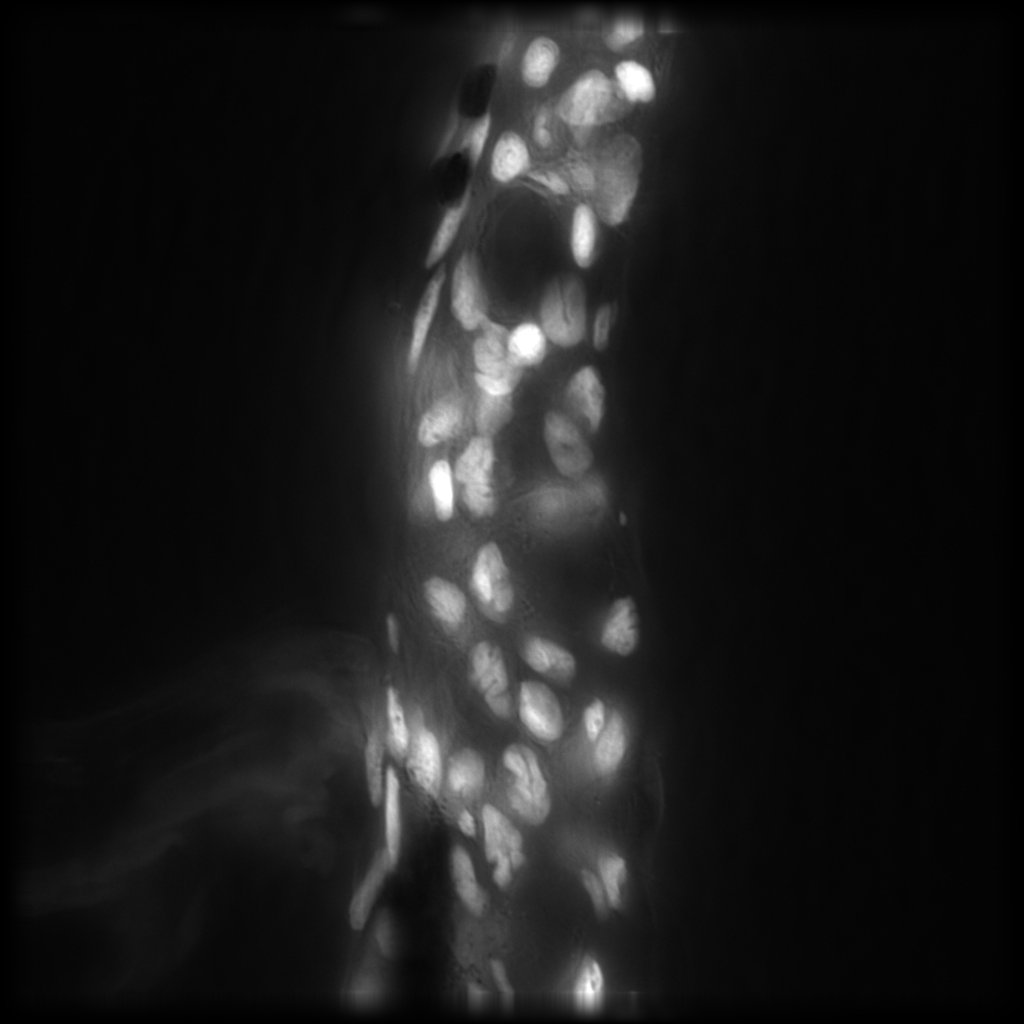

Supplement: Supplementary file 6 — Source data Fig. 4 [file 44318_2025_482_MOESM6_ESM.zip › Fig4 new 5/Fig 4A new 5A/PCNA skin/wt 60x PCNA skin.tif]

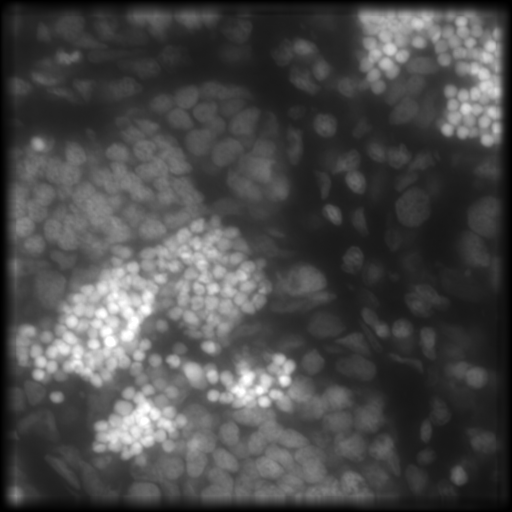

Supplement: Supplementary file 6 — Source data Fig. 4 [file 44318_2025_482_MOESM6_ESM.zip › Fig4 new 5/Fig 4A new 5A/PCNA testis/RAW sting -_- 60x PCNA testis.tif]

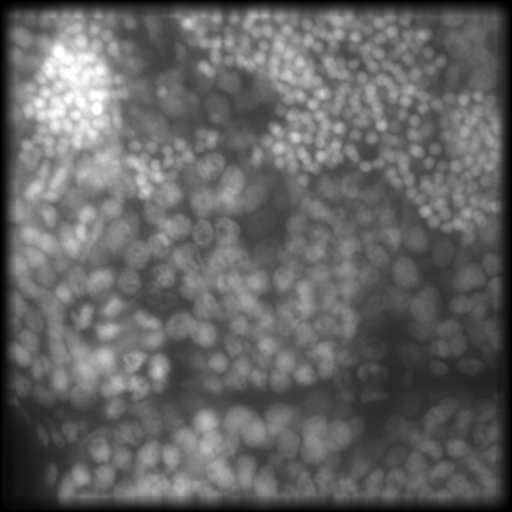

Supplement: Supplementary file 6 — Source data Fig. 4 [file 44318_2025_482_MOESM6_ESM.zip › Fig4 new 5/Fig 4A new 5A/PCNA testis/RAW tert -_- 60x PCNA testis.tif]

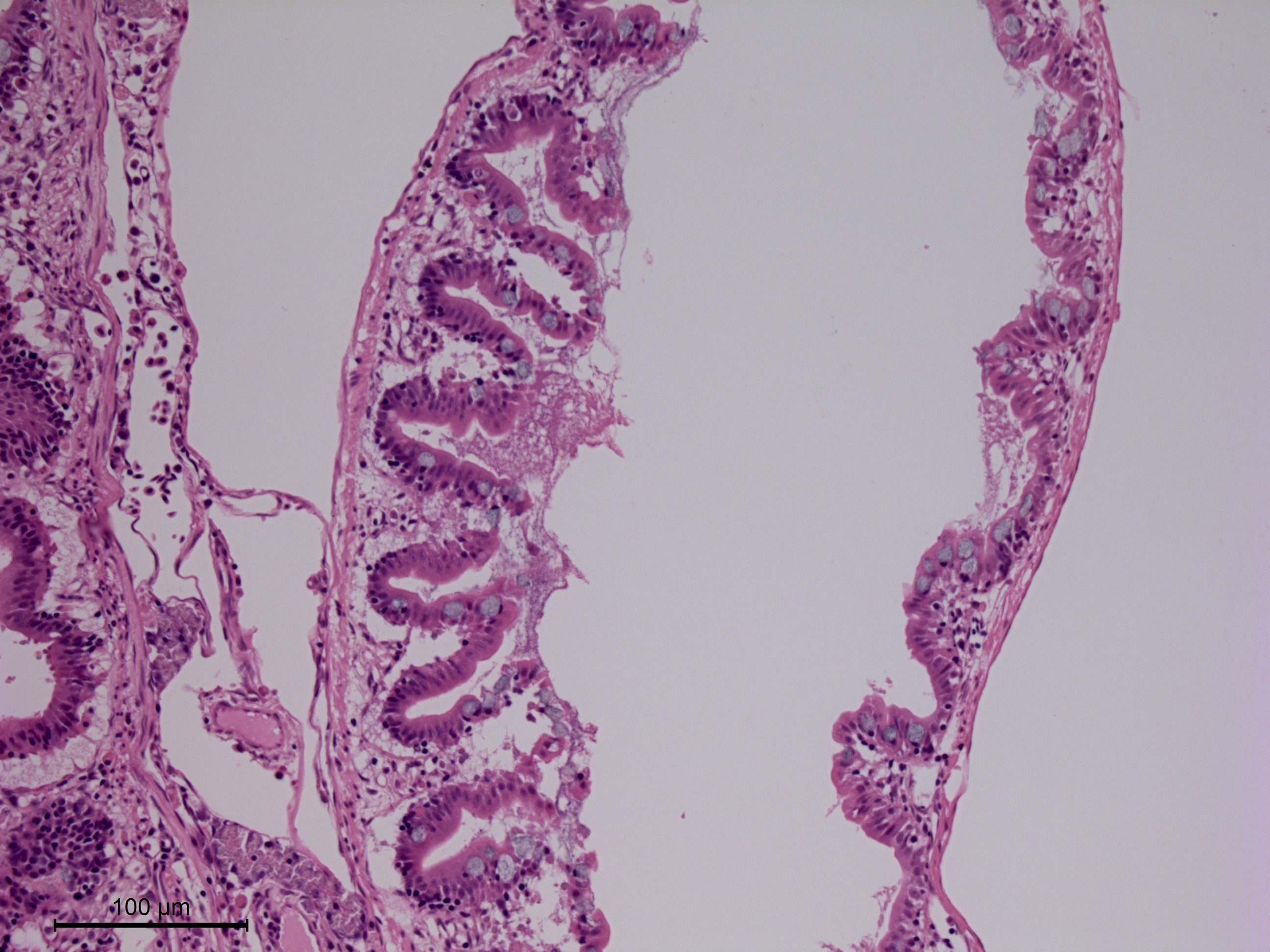

Supplement: Supplementary file 6 — Source data Fig. 4 [file 44318_2025_482_MOESM6_ESM.zip › Fig4 new 5/Fig 4F new 5F/20x sting intestine.tif]

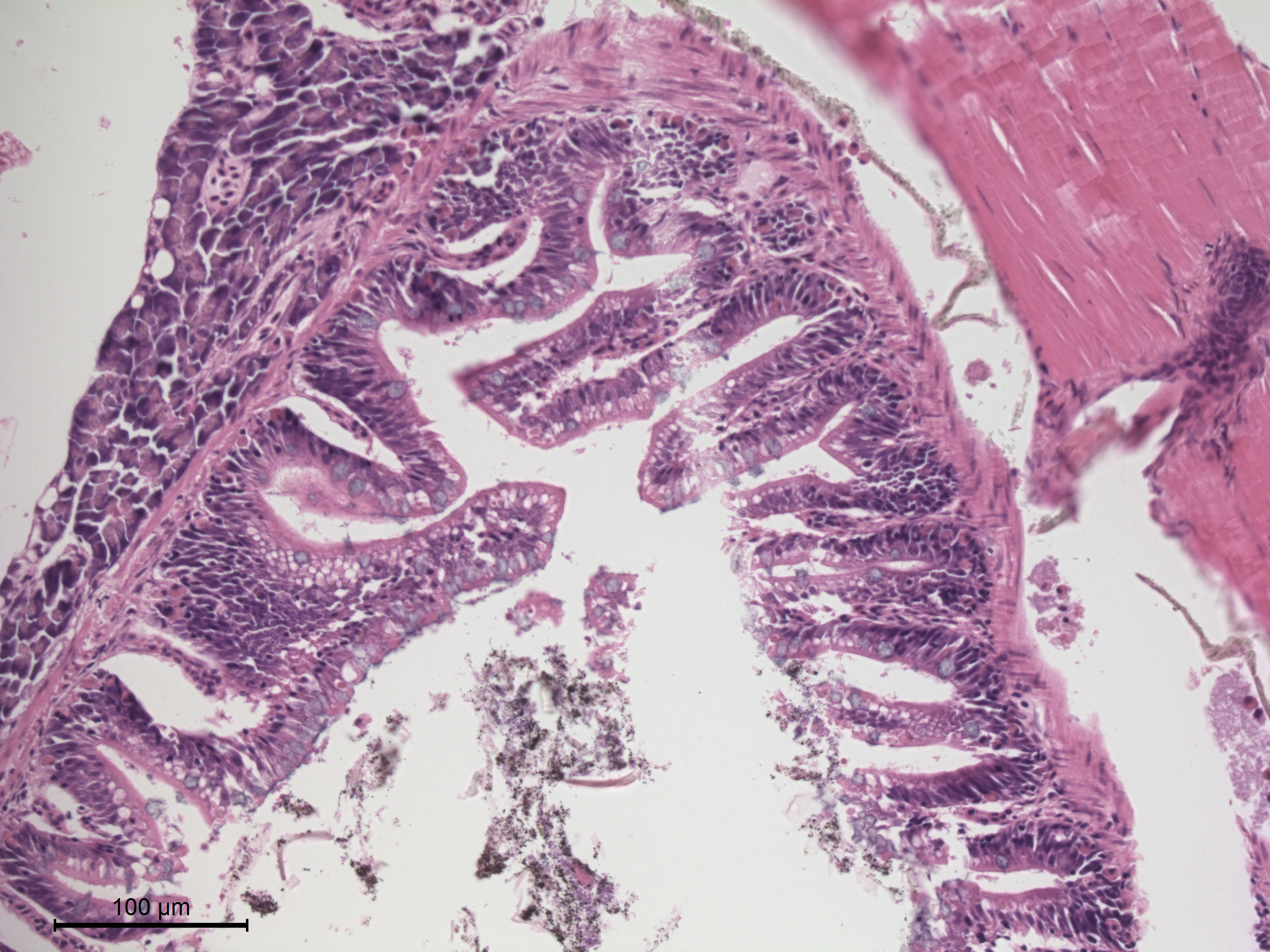

Supplement: Supplementary file 6 — Source data Fig. 4 [file 44318_2025_482_MOESM6_ESM.zip › Fig4 new 5/Fig 4F new 5F/20x sting tert intestine.tif]

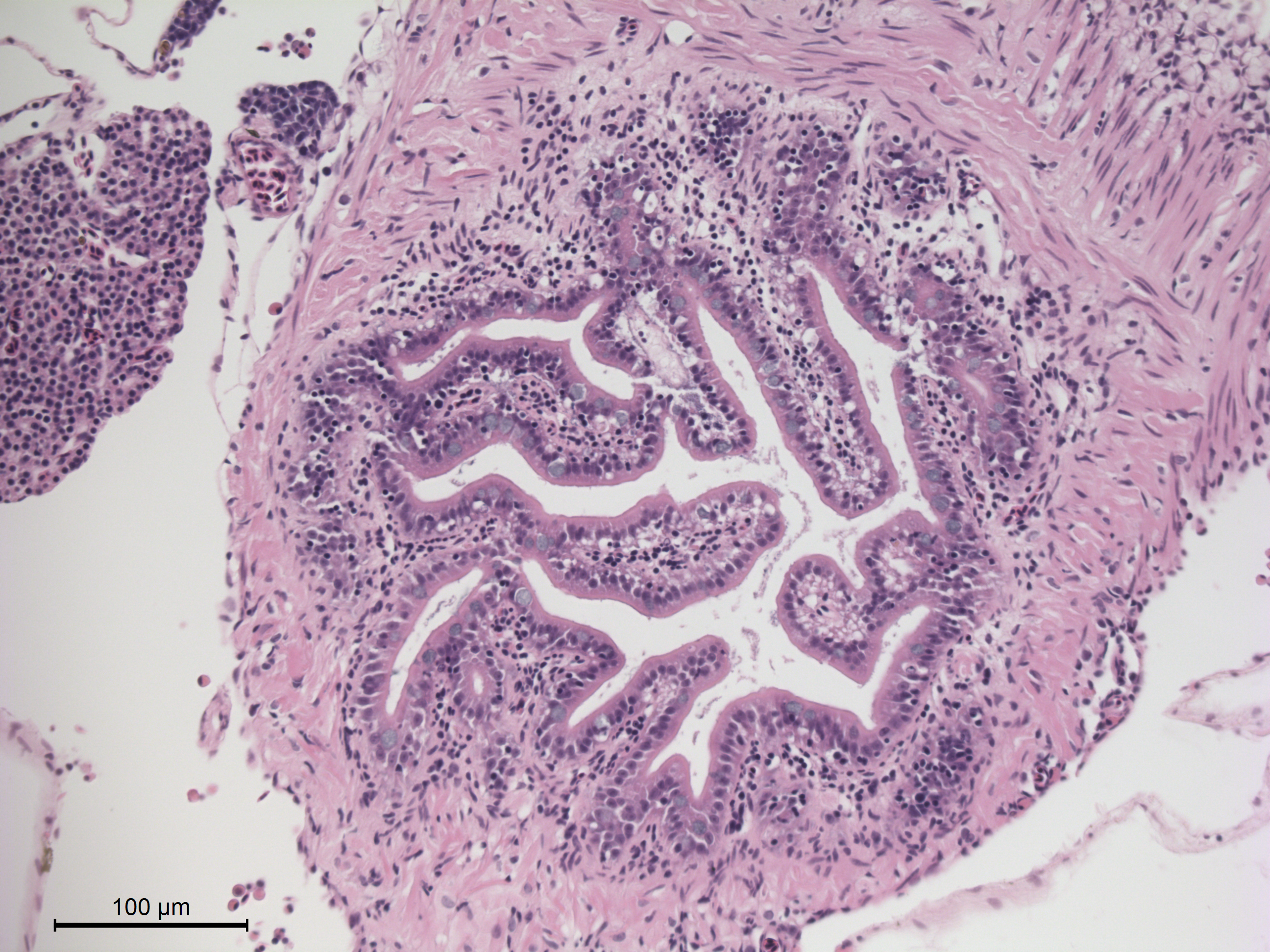

Supplement: Supplementary file 6 — Source data Fig. 4 [file 44318_2025_482_MOESM6_ESM.zip › Fig4 new 5/Fig 4F new 5F/20x tert intestine.tif]

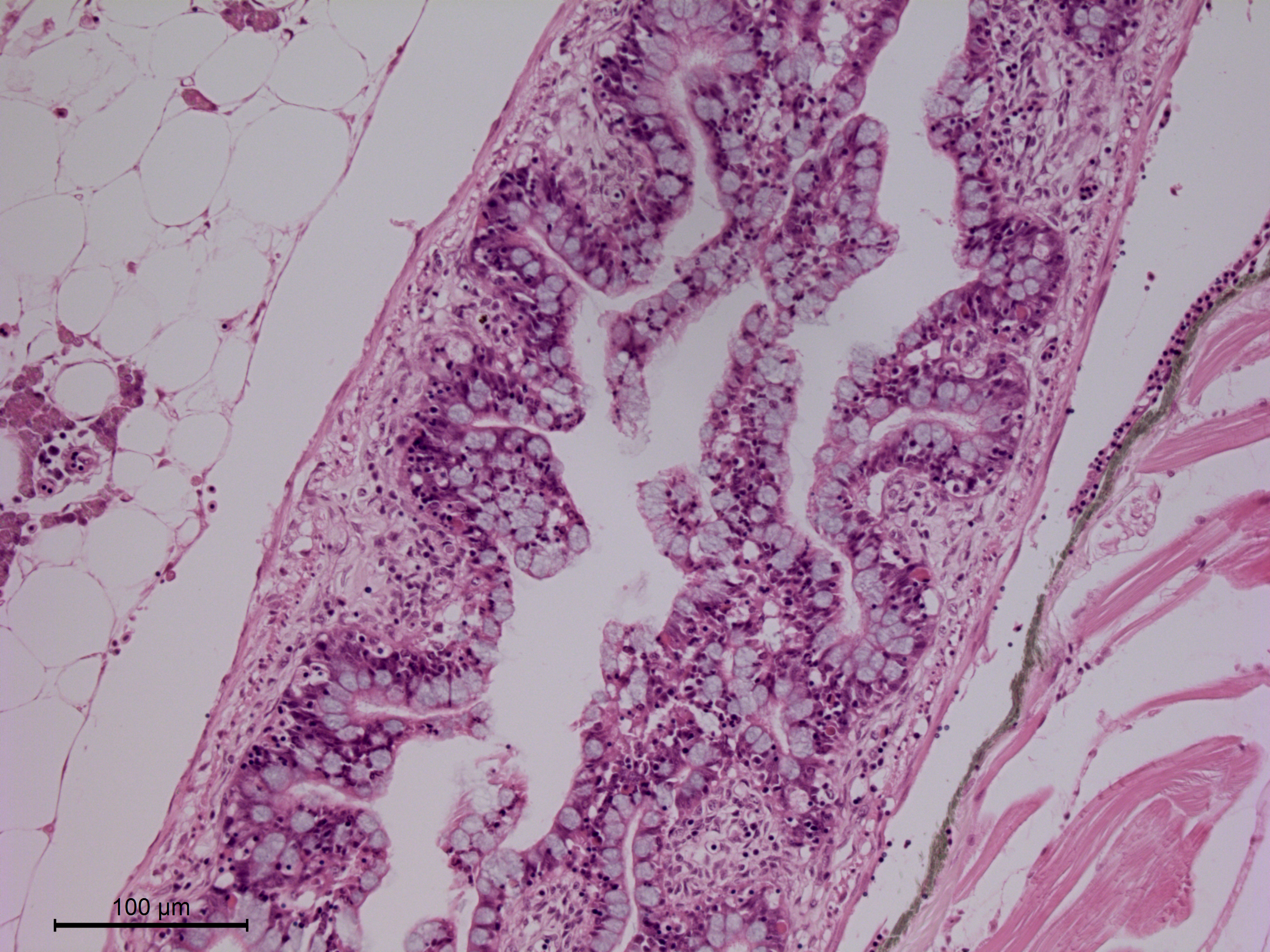

Supplement: Supplementary file 6 — Source data Fig. 4 [file 44318_2025_482_MOESM6_ESM.zip › Fig4 new 5/Fig 4F new 5F/20x wt intestine.tif]

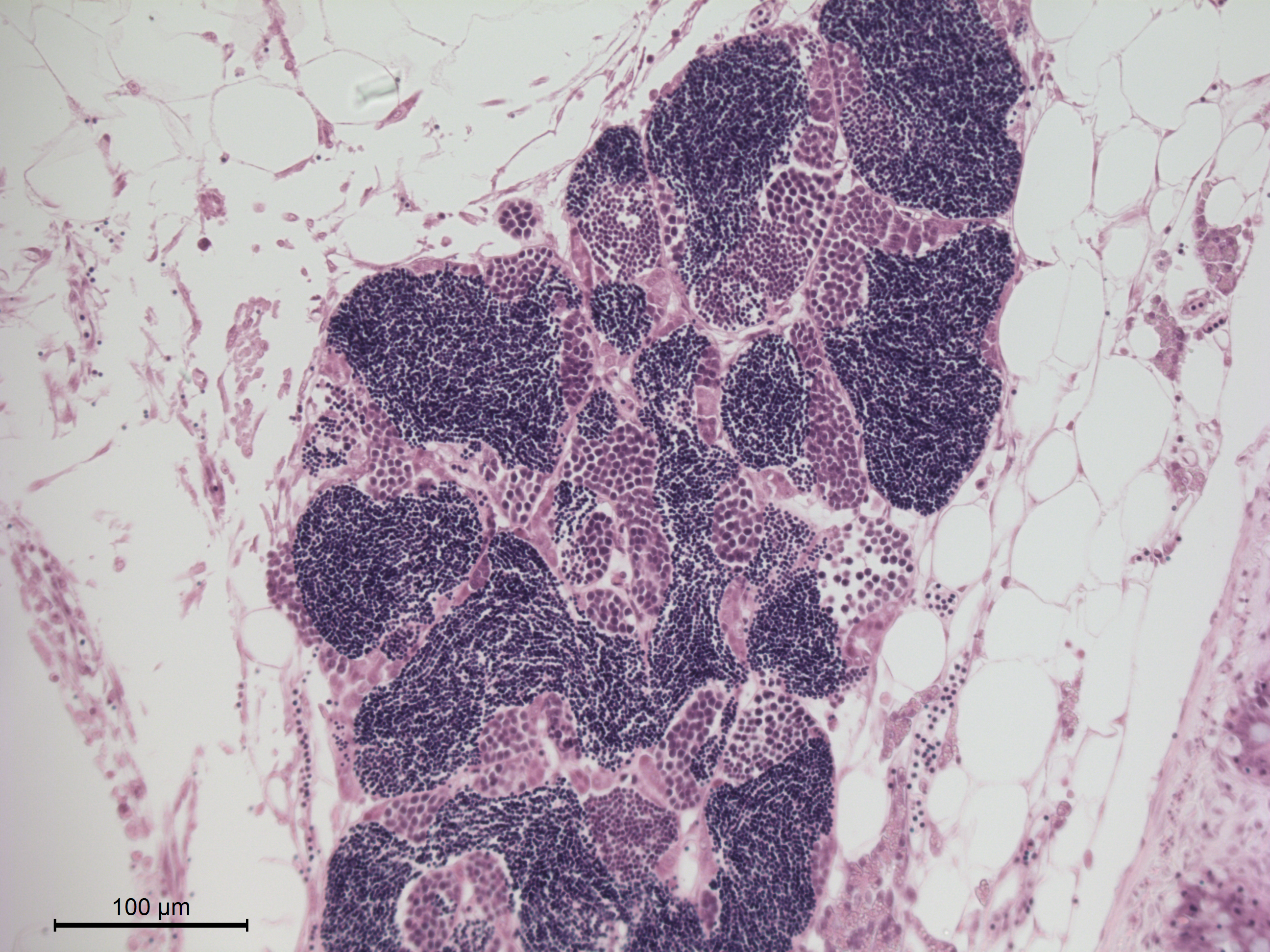

Supplement: Supplementary file 6 — Source data Fig. 4 [file 44318_2025_482_MOESM6_ESM.zip › Fig4 new 5/Fig 4H new 5H/20x sting tert testis.tif]

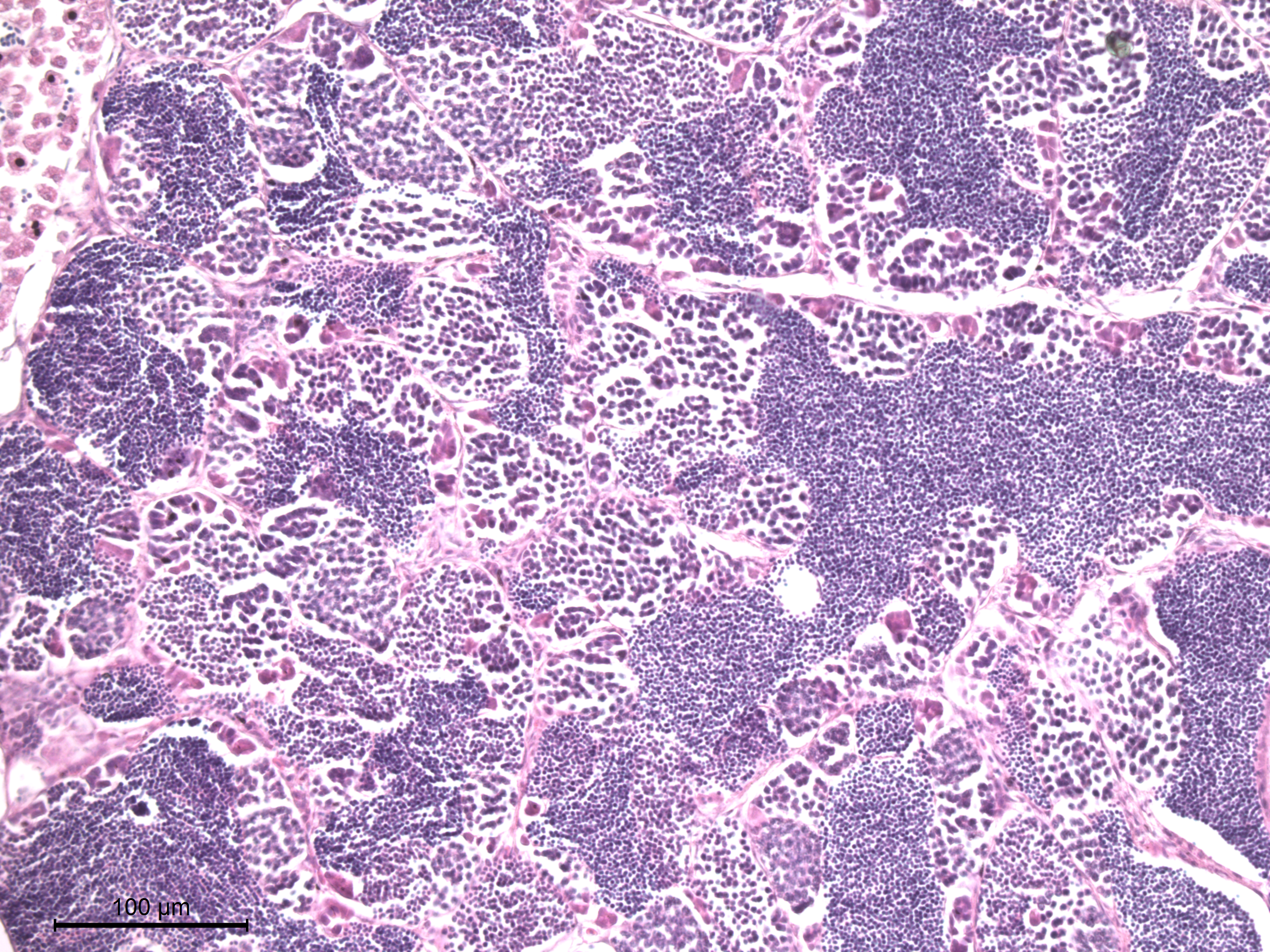

Supplement: Supplementary file 6 — Source data Fig. 4 [file 44318_2025_482_MOESM6_ESM.zip › Fig4 new 5/Fig 4H new 5H/20x sting testis.tif]

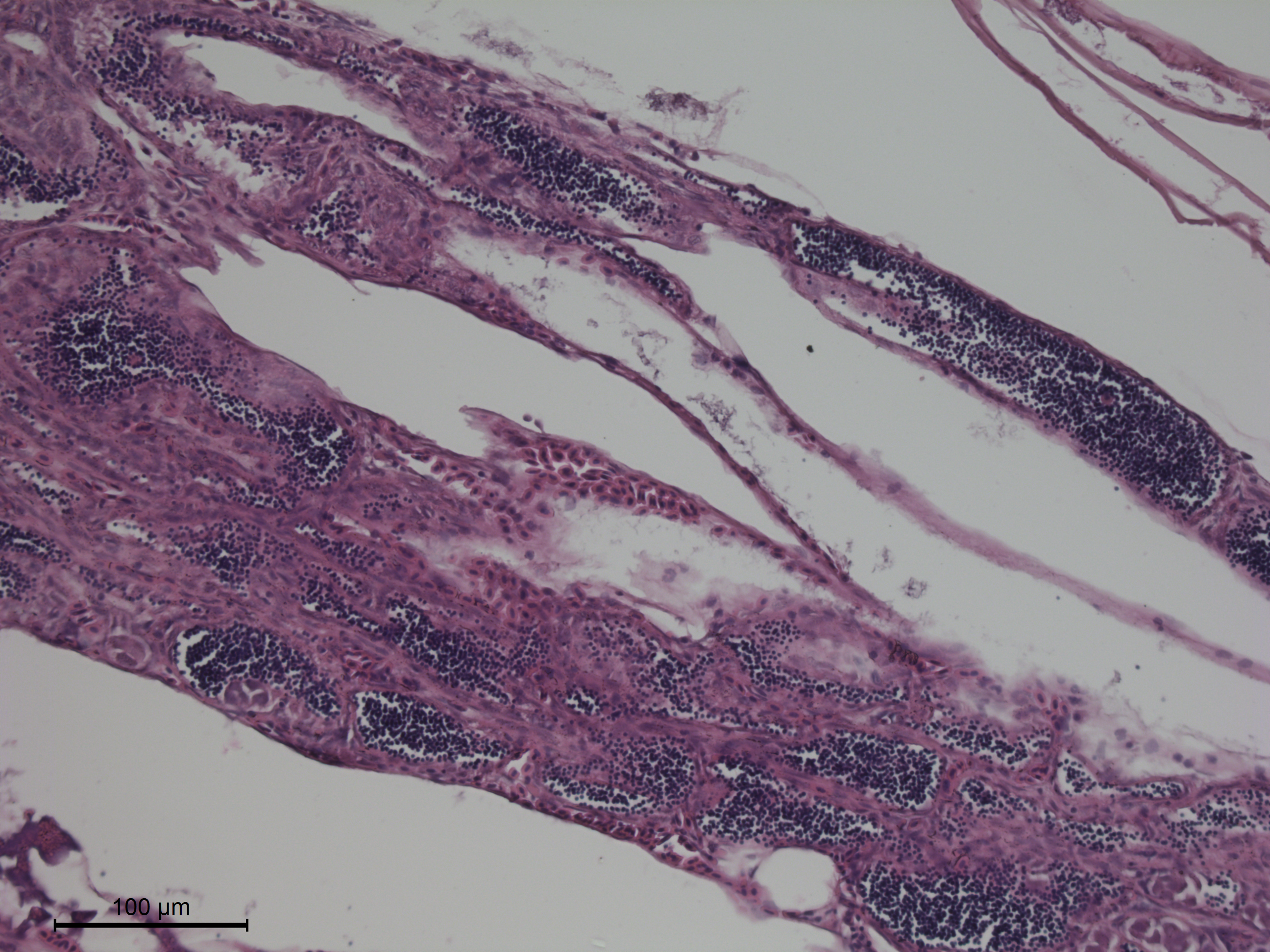

Supplement: Supplementary file 6 — Source data Fig. 4 [file 44318_2025_482_MOESM6_ESM.zip › Fig4 new 5/Fig 4H new 5H/20x tert testis.tif]

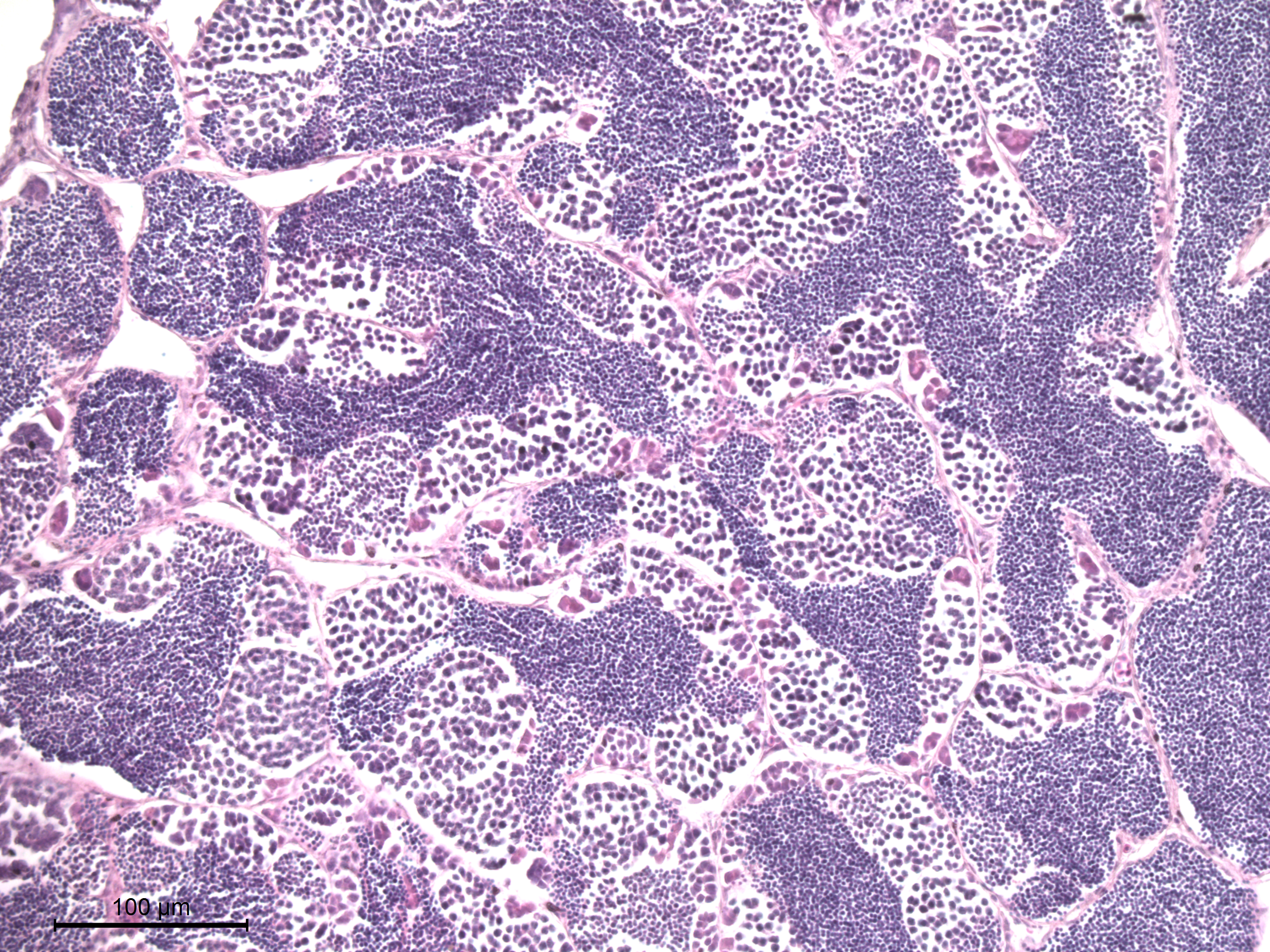

Supplement: Supplementary file 6 — Source data Fig. 4 [file 44318_2025_482_MOESM6_ESM.zip › Fig4 new 5/Fig 4H new 5H/20x wt testis.tif]
