# Supplementary material for: cGAS-STING are responsible for premature aging of telomerase-deficient zebrafish
Source: EMBO J. 2025 Jun 9;44(17):4666–80. doi: 10.1038/s44318-025-00482-5 (PMC12402478; doi:10.1038/s44318-025-00482-5)
Supplement: Supplementary file 4 — Source data Fig. 2 [file 44318_2025_482_MOESM4_ESM.zip › Fig2 new 3/Fig2F new 3F/p53 wb.pptx]

## Slide 1
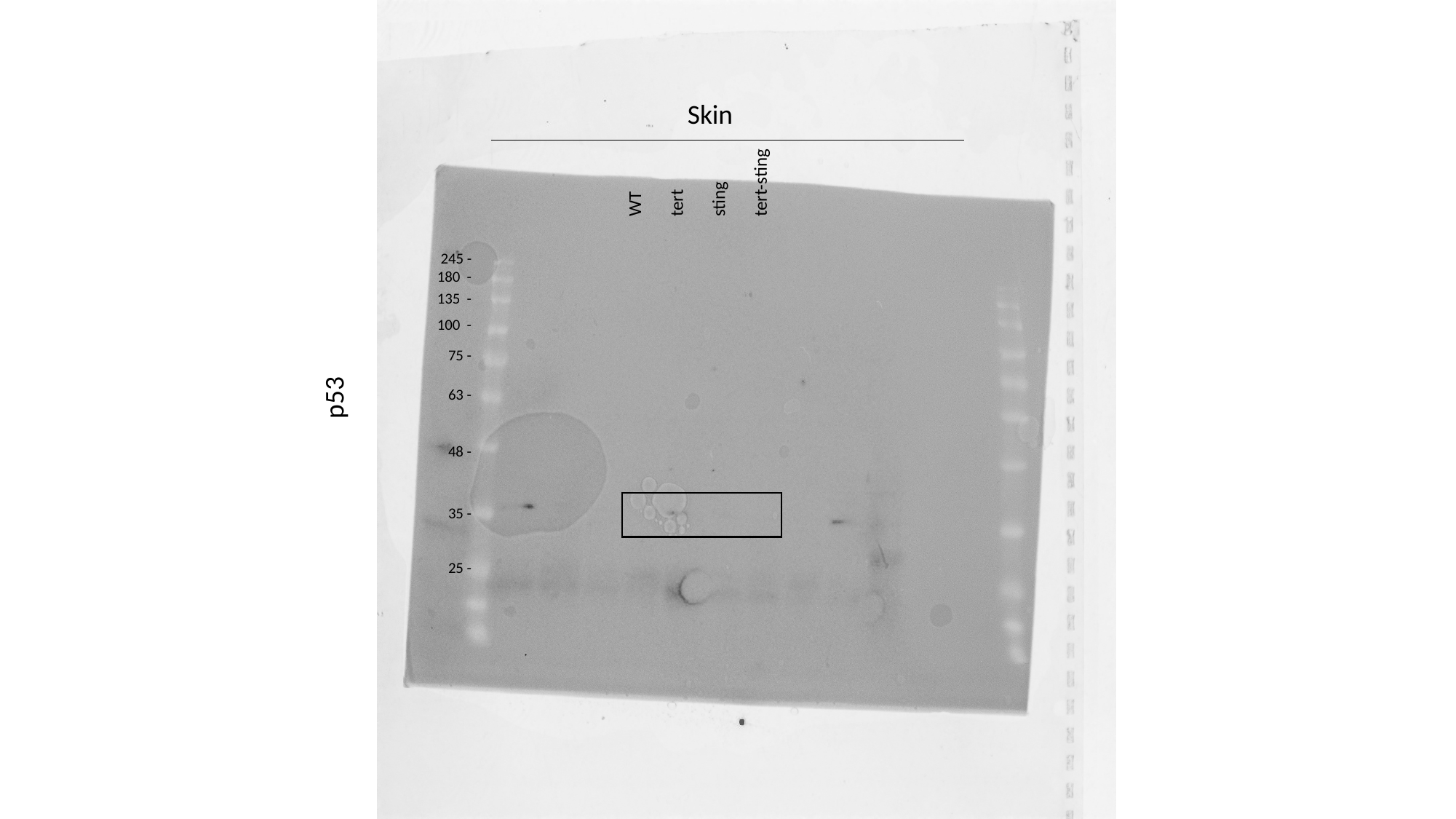

Skin
| WT | tert | sting | tert-sting |
| --- | --- | --- | --- |
245 -
180 -
135 -
100 -
75 -
p53
63 -
48 -
35 -
25 -

## Slide 2
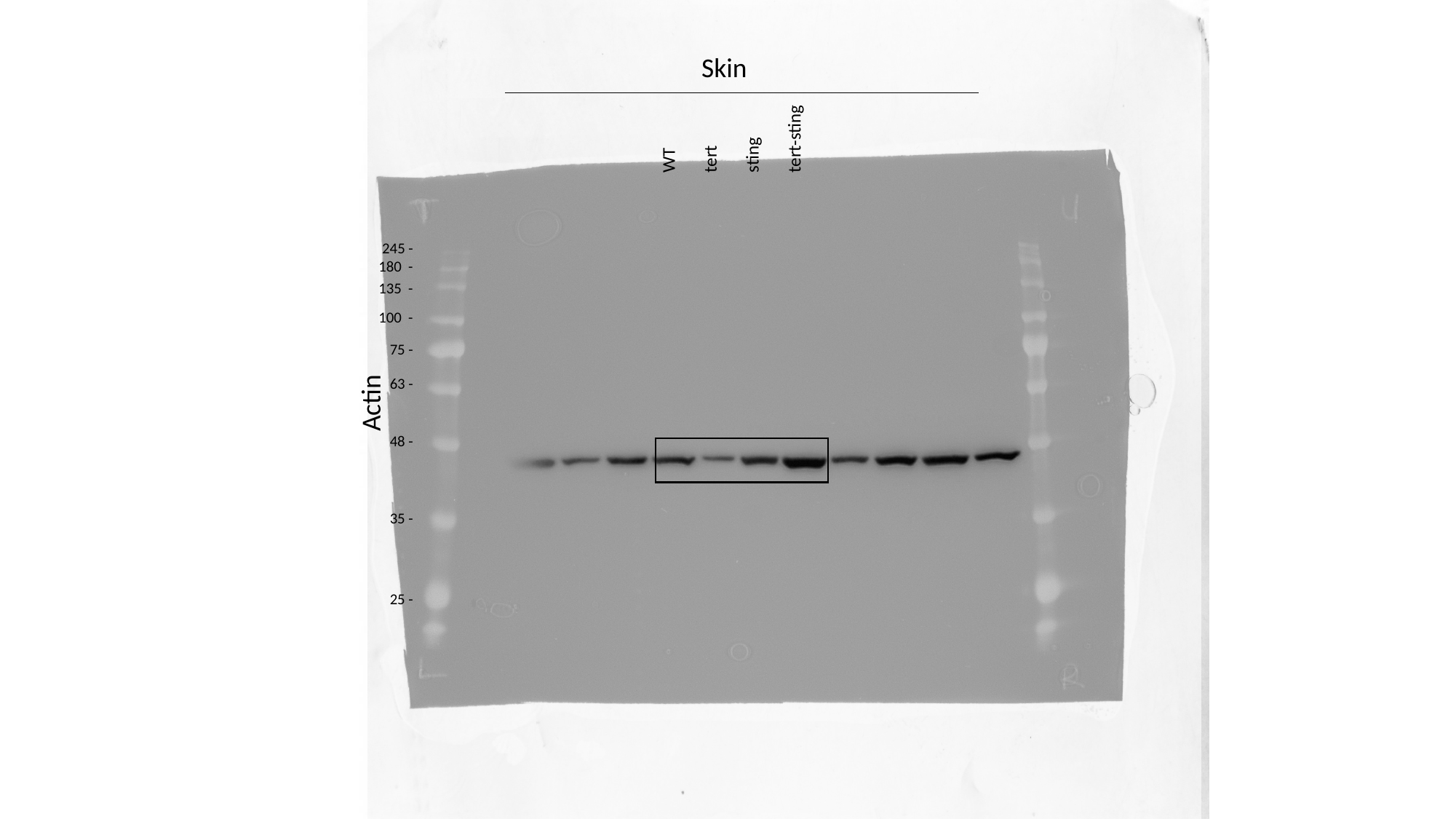

Skin
| WT | tert | sting | tert-sting |
| --- | --- | --- | --- |
245 -
180 -
135 -
100 -
75 -
63 -
Actin
48 -
35 -
25 -

## Slide 3
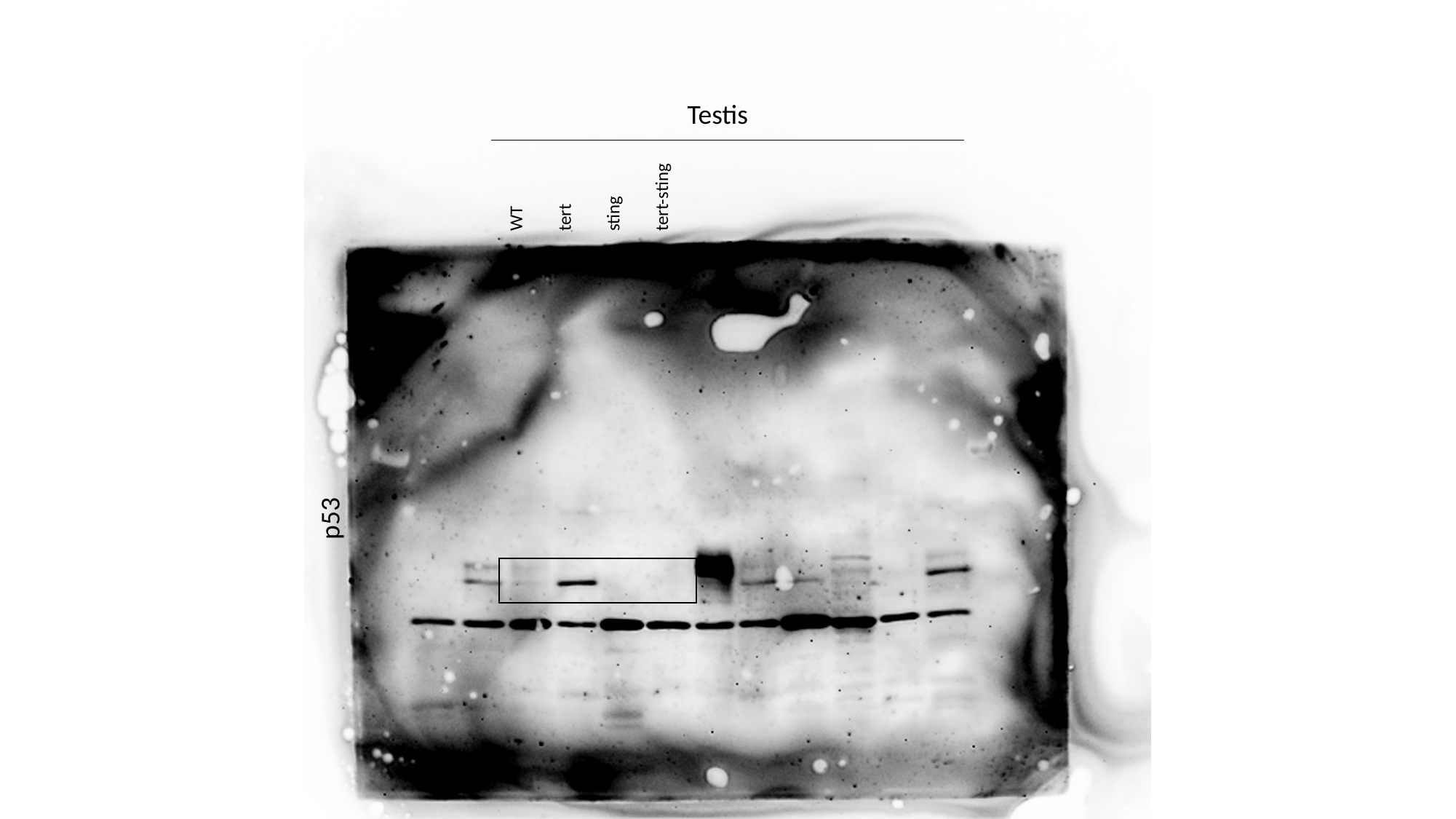

Testis
| WT | tert | sting | tert-sting |
| --- | --- | --- | --- |
p53

## Slide 4
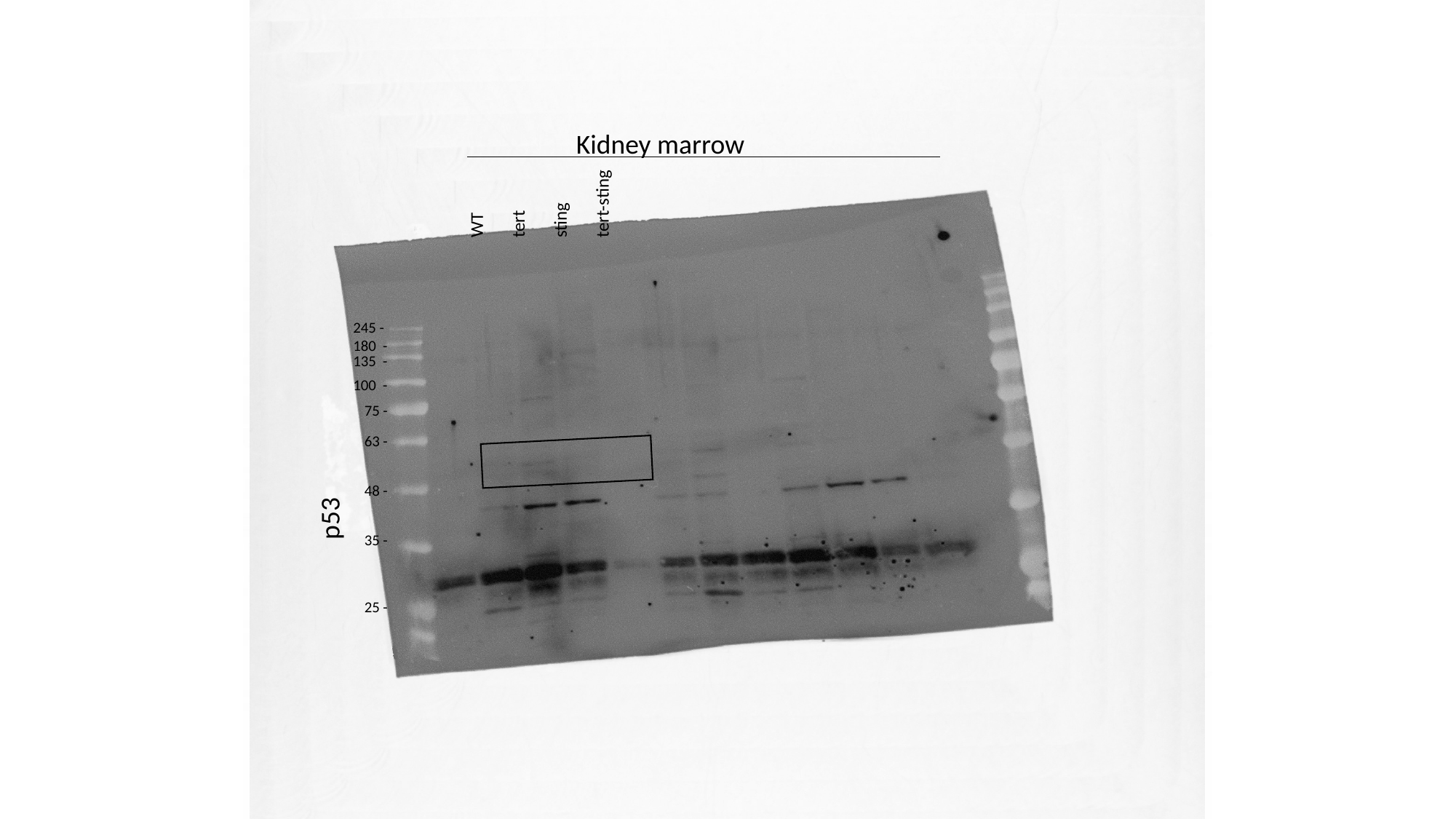

Kidney marrow
| WT | tert | sting | tert-sting |
| --- | --- | --- | --- |
245 -
180 -
135 -
100 -
75 -
63 -
48 -
p53
35 -
25 -

## Slide 5
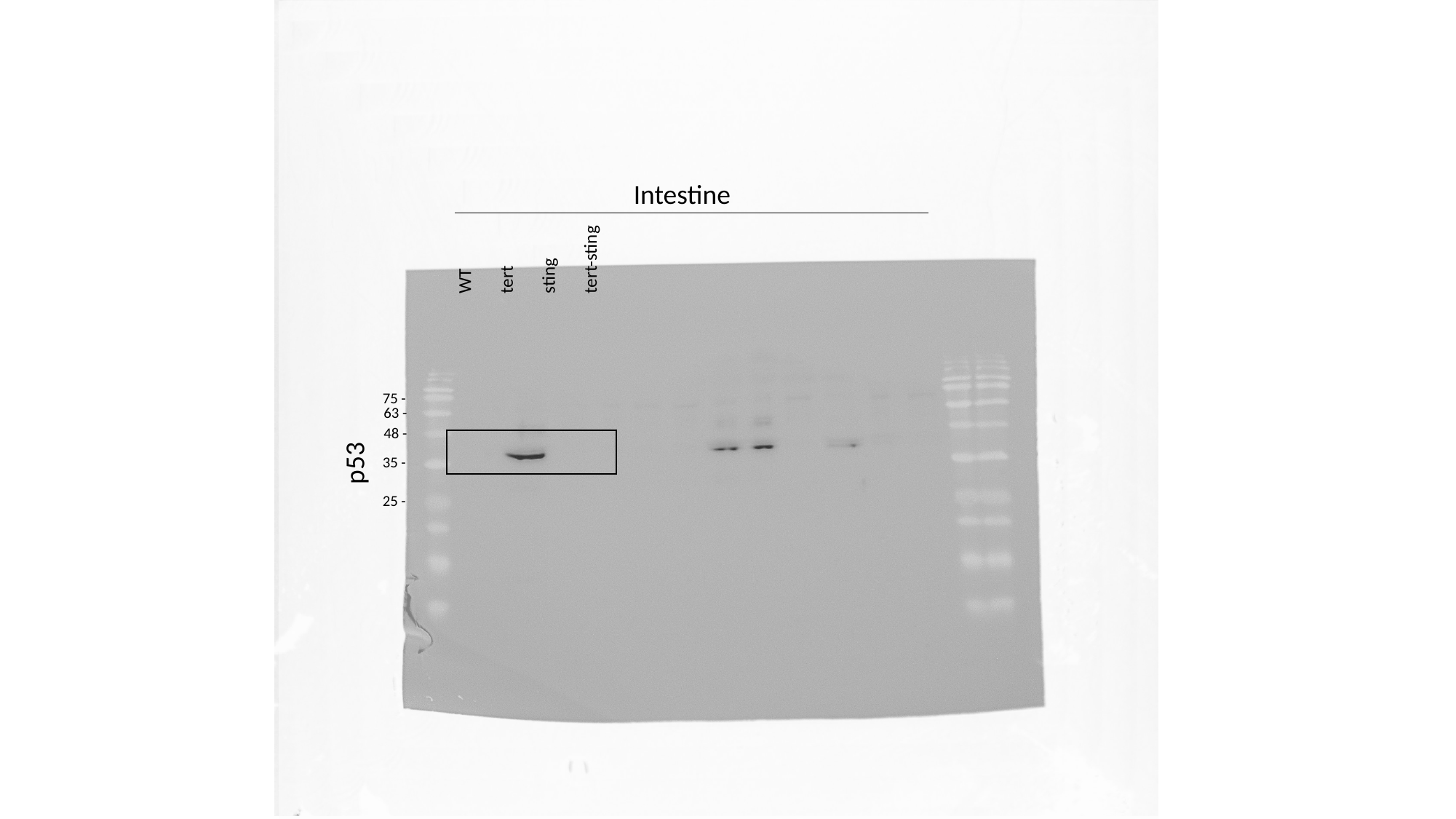

Intestine
| WT | tert | sting | tert-sting |
| --- | --- | --- | --- |
75 -
63 -
48 -
p53
35 -
25 -

## Slide 6
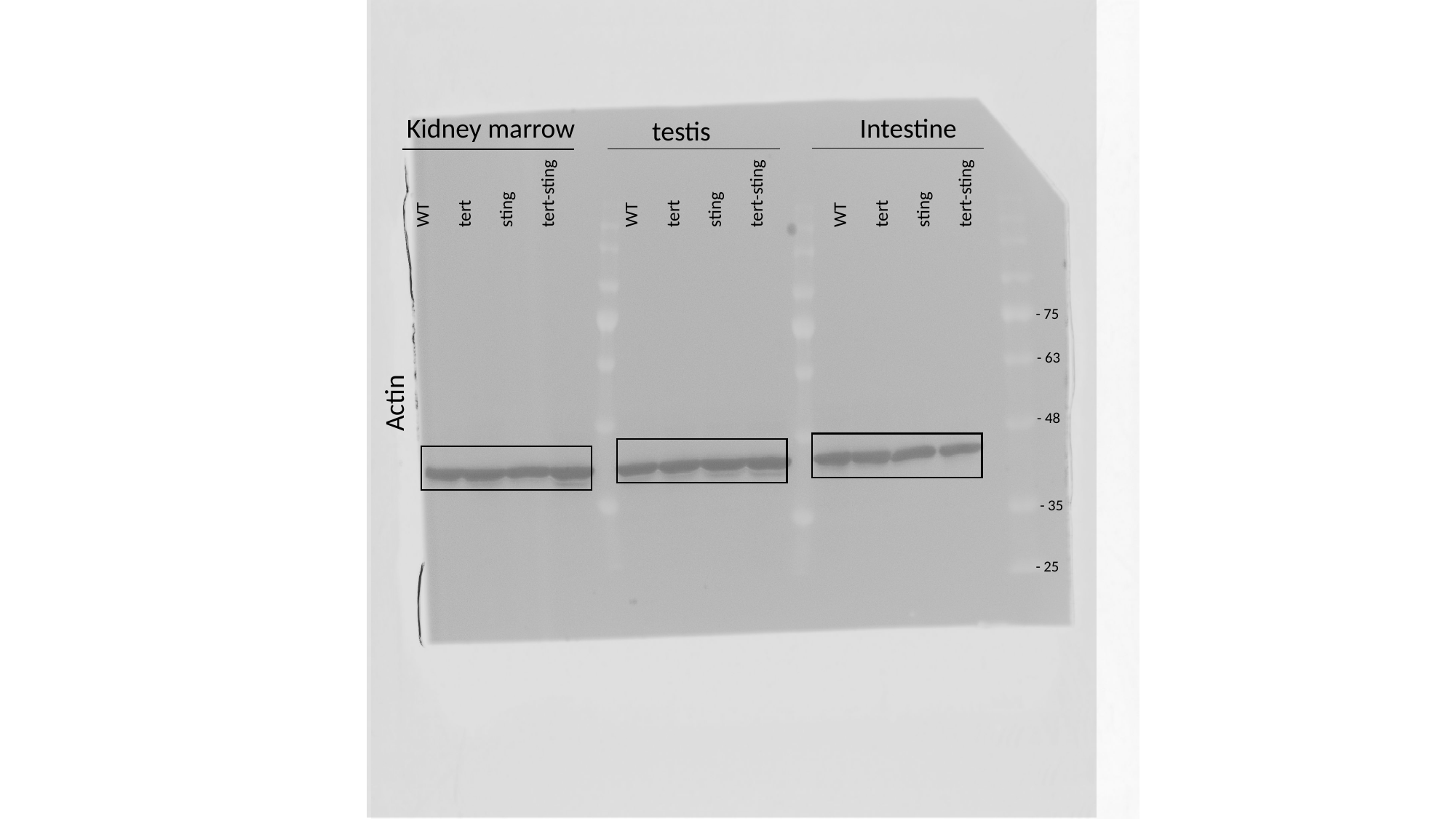

Kidney marrow
Intestine
testis
| WT | tert | sting | tert-sting | | WT | tert | sting | tert-sting | | WT | tert | sting | tert-sting |
| --- | --- | --- | --- | --- | --- | --- | --- | --- | --- | --- | --- | --- | --- |
- 75
- 63
Actin
- 48
- 35
- 25
